# Genetic examination of the Mood Disorder Questionnaire and its relationship with bipolar disorder

**DOI:** 10.1002/ajmg.b.32938

**Published:** 2023-05-13

**Authors:** Jessica Mundy, Christopher Hübel, Brett N. Adey, Helena L. Davies, Molly R. Davies, Jonathan R. I. Coleman, Matthew Hotopf, Gursharan Kalsi, Sang Hyuck Lee, Andrew M. McIntosh, Henry C. Rogers, Thalia C. Eley, Robin M. Murray, Evangelos Vassos, Gerome Breen

**Affiliations:** ^1^ Institute of Psychiatry, Psychology and Neuroscience, King's College London London UK; ^2^ UK National Institute for Health and Care Research (NIHR) Biomedical Research Centre South London and Maudsley Hospital London UK; ^3^ National Centre for Register‐based Research, Aarhus Business and Social Sciences Aarhus University Aarhus Denmark; ^4^ South London and Maudsley NHS Foundation Trust Bethlem Royal Hospital Kent UK; ^5^ Division of Psychiatry, Centre for Clinical Brain Sciences University of Edinburgh Edinburgh UK

**Keywords:** bipolar disorder, factor analysis, genetic correlation, genome‐wide association study, hypomania, mania

## Abstract

The Mood Disorder Questionnaire (MDQ) is a common screening tool for bipolar disorder that assesses manic symptoms. Its utility for genetic studies of mania or bipolar traits has not been fully examined. We psychometrically compared the MDQ to self‐reported bipolar disorder in participants from the United Kingdom National Institute of Health and Care Research Mental Health BioResource. We conducted genome‐wide association studies of manic symptom quantitative traits and symptom subgroups, derived from the MDQ items (*N* = 11,568–19,859). We calculated genetic correlations with bipolar disorder and other psychiatric and behavioral traits. The MDQ screener showed low positive predictive value (0.29) for self‐reported bipolar disorder. Neither concurrent nor lifetime manic symptoms were genetically correlated with bipolar disorder. Lifetime manic symptoms had a highest genetic correlation (*r*_g_ = 1.0) with posttraumatic stress disorder although this was not confirmed by within‐cohort phenotypic correlations (*r*_p_ = 0.41). Other significant genetic correlations included attention deficit hyperactivity disorder (*r*_g_ = 0.69), insomnia (*r*_g_ = 0.55), and major depressive disorder (*r*_g_ = 0.42). Our study adds to existing literature questioning the MDQ's validity and suggests it may capture symptoms of general distress or psychopathology, rather than hypomania/mania specifically, in at‐risk populations.

## 1 INTRODUCTION

Mania involves periods of elevated, expansive, or irritable mood. Symptoms may include feeling energetic or hyperactive, having unusually inflated self‐confidence, requiring little or no sleep, or engaging in behaviors that some might consider impulsive or risky. Hypomania involves these symptoms to a milder degree (American Psychiatric Association, 2013). Mania and hypomania generally alternate with episodes of depressed mood in bipolar disorder type I and type II, respectively. The lifetime prevalences of these psychiatric disorders are 0.6% and 0.4%, respectively (Merikangas et al., 2011).

Individuals with bipolar disorder usually present with other psychiatric symptoms in the first instance, particularly depression (Musliner & Østergaard, 2018) and the time between initial presentation to services and receiving the correct diagnosis can be over ten years (Hirschfeld, Lewis, & Vornik, 2003; Lish et al., 1994). Therefore, some individuals presenting with psychiatric problems, especially depression, anxiety, and substance use disorders, may later develop hypomania or mania (Baryshnikov et al., 2020; Kessing et al., 2017; Zimmerman et al., 2009). Identifying “hidden” bipolar disorder patients can aid clinicians in earlier diagnosis and prescribing of correct medication (e.g., mood stabilizers) which reduces risk of antidepressant‐induced mania, rapid cycling, and the costs associated with delayed treatment (Hirschfeld, 2010; Zimmerman, 2012).

Several screening tools have been developed to assess possible hypomania/mania in at‐risk individuals. One of the most widely used is the Mood Disorder Questionnaire (MDQ) which involves questions about 13 aspects of mania, symptom duration, functional impairment, and family history of bipolar disorder (Hirschfeld, 2010). The MDQ has been translated into 16 languages and is used globally (Hirschfeld, 2010; Zimmerman et al., 2009). Bipolar disorder is heritable (Mullins et al., 2021; Stahl et al., 2019), and the MDQ has been applied in genetic studies of mania with mixed results. A twin study showed that the heritable basis of MDQ‐assessed hypomania was moderately correlated with the heritable basis for bipolar disorder (*r*_g_ = 0.40) in a non‐clinical youth sample, which mirrored their phenotypic correlation (*r*_ph_ = 0.39). However, hypomania did not show a significant correlation with bipolar disorder polygenic risk scores (PRSs) based on common genetic variants (Hosang et al., 2022). Thus, the genetic basis of the symptoms assessed by the MDQ, and their relationship with bipolar disorder, warrant further examination.

The Genetic Links to Anxiety and Depression (GLAD) Study, a nationwide resource of participants with a lifetime occurrence of depression and anxiety disorders, used the MDQ to assess lifetime presence of hypomanic/manic symptoms. Participants were also asked about whether they had received a diagnosis of bipolar disorder by a professional. This large cohort of participants with mental health disorders and genetic data available offers an opportunity to examine the validity of the MDQ among individuals who are at increased risk of developing bipolar disorder.

Here, we investigated the validity of the MDQ. First, we assessed the psychometric properties of the MDQ as a screener in our sample, based on self‐reported diagnoses of bipolar disorder. Since self‐reported diagnoses of mental health disorders may be inaccurate (Davies et al., 2022), we performed an extra step to validate the MDQ using genomic methods. We calculated the genetic correlation between the number of manic symptoms that participants reported in the MDQ and the largest available GWAS of bipolar disorder from the Psychiatric Genomics Consortium (PGC) (Mullins et al., 2021). All cases included in the PGC GWAS (*N* = 41,917) met DSM‐IV or ICD‐9/10 criteria for bipolar disorder. These diagnoses were obtained from diagnostic interviews, clinician‐administered checklists, or medical records. Thus, genetic correlations with this GWAS can be used to assess the external validity of the MDQ as a tool for assessing hypomania/mania.

We assessed the MDQ items as a quantitative score in two ways: (a) concurrent symptoms during one time period and (b) cumulative symptoms across the lifetime (not specifying co‐occurrence). Since nine of the MDQ items equate to the diagnostic criteria for hypomania/mania in the DSM‐5, we hypothesized that both quantitative measures would show significant positive genetic correlations, a measure of the relationship between two polygenic phenotypes (van Rheenen et al., 2019), with bipolar disorder. We expected the genetic correlation to be greater with the measure of concurrent symptoms because experiencing multiple symptoms within one week or four days is a requirement for a bipolar disorder type I and type II diagnosis respectively (American Psychiatric Association, 2013). Furthermore, a positive screen in the MDQ is made on the basis of concurrent symptoms (Hirschfeld et al., 2000). In addition to bipolar disorder, we calculated genetic correlations with 34 other psychiatric and behavioral traits. We expected MDQ‐assessed manic symptoms to have a higher genetic correlation with bipolar disorder compared to the other traits tested.

Since symptoms that collectively underlie a quantitative trait may vary in terms of their biology (Nagel et al., 2018; Thorp et al., 2020), we hypothesized that genetic risk for symptom subgroups of the MDQ, identified from factor analyses, would show genetic heterogeneity. We made no *a priori* predictions about the direction or strength of the overlap.

## 2 METHODS

All code is available on GitHub (https://github.com/tnggroup/genetics_MDQ). This study was pre‐registered on the Open Science Framework.

### 2.1 Study design

Data were examined from participants in the mental health arm of the National Institute of Health and Care Research (NIHR) BioResource in the United Kingdom. The largest group of participants were recruited via the Genetic Links to Anxiety and Depression (GLAD) Study (https://gladstudy.org.uk/), an online research platform for individuals with lifetime anxiety and/or major depressive disorder (MDD) (Davies et al., 2019). Recruitment into GLAD began in September 2018 and was conducted via social media campaigns and NHS sites. Other participants were from the COVID‐19 Psychiatry and Neurological Genetics (COPING) Study (https://gladstudy.org.uk/all-projects/current-projects/coping-study/). These individuals were initially recruited into the NIHR BioResource from various cohorts via several means (listed in Tables S18a and S18b). They were secondarily invited into the COPING Study (henceforth referred to as “COPING NBR participants”).

Individuals were eligible to participate if they were aged 16+ and lived in the United Kingdom. GLAD participants were additionally required to have experienced MDD or an anxiety disorder in their lifetime. All participants provided demographic information, mental health histories, and some provided a saliva or blood sample. The COPING baseline survey comprised many of the same questionnaires from the GLAD sign‐up survey which allowed for parallel assessments. All questionnaires were acquired using Qualtrics survey software (Qualtrics, Provo, UT). We analyzed data from participants who completed the GLAD Study sign‐up survey or COPING baseline survey between 17th September 2018 and 3rd September 2021.

### 2.2 Study sample

We analyzed data from individuals with experience of MDD and/or anxiety. COPING NBR participants who met symptom‐based diagnostic criteria for MDD and/or any anxiety disorder were combined with GLAD participants to create a cohort who had been affected by these common mental health disorders (Figure S28). To compare the distribution of manic symptoms between those affected and unaffected by MDD and/or anxiety, we also measured lifetime MDQ items in participants with no history of these disorders. COPING NBR participants who did not meet criteria for MDD or any anxiety disorder were categorized as “unaffected participants.” COPING NBR participants without the data required to determine MDD or anxiety diagnosis were excluded (details on symptom‐based diagnostic criteria in Supplementary Methods).

### 2.3 Ethics

Full informed consent was obtained from all participants. Ethical approval for the GLAD Study was granted by the London‐Fulham Research Ethics Committee (REC reference: 18/LO/1218) and for COPING, by the NHS Health Research Authority, South West—Central Bristol Research Ethics Committee (20/SW/0078).

### 2.4 Measures

#### 2.4.1 The Mood Disorder Questionnaire

Lifetime experience of 13 hypomanic/manic symptoms were assessed via the MDQ (Table 1) (henceforth “lifetime manic symptoms”). Participants who endorsed more than one of the lifetime manic symptoms were then asked whether these symptoms happened during the same period. The participants who answered “*Yes*” were subsequently presented with a list of their previously endorsed symptoms and were asked to “*select all that occurred during the same period of time*” (henceforth “concurrent manic symptoms”).

**TABLE 1:** Hypomanic/manic symptoms assessed by the Mood Disorder Questionnaire (MDQ).

Question in MDQ	Abbreviated name	Endorsement (concurrent in affected participants)	Endorsement (lifetime in affected participants)	Endorsement (lifetime in unaffected participants)
		*N* = 30,342	*N* = 47,787	*N* = 6308
… you felt so good or so hyper that other people thought you were not your normal self or you were so hyper that you got into trouble?	Hyperactivity	32.3%	36.2%	2.4%
… you were so irritable that you shouted at people or started fights or arguments?	Irritability	61.8%	71.3%	17.5%
… you felt much more self‐confident than usual?	More self‐confidence	34.3%	38.0%	8.4%
… you got much less sleep than usual and found you did not really miss it?	Decreased sleep	53.9%	40.7%	11.9%
… you were much more talkative or spoke much faster than usual?	More talkative	42.1%	43.2%	5.0%
… thoughts raced through your head or you could not slow your mind down?	Racing thoughts	77.6%	73.9%	15%
… you were so easily distracted by things around you that you had trouble concentrating or staying on track?	Concentration difficulties	70.8%	71.8%	14.3%
… you had much more energy than usual?	More energy	32.6%	35.7%	8.3%
… you were much more active or did many more things than usual?	More active	30.0%	37.3%	11.7%
… you were much more social or outgoing than usual, for example, you telephoned friends in the middle of the night?	More sociable	20.7%	21.7%	2.0%
… you were much more interested in sex than usual?	Higher libido	28.7%	33.9%	7.4%
… you did things that were unusual for you or that other people might have thought were excessive, foolish, or risky?	Risky behavior	36.7%	36.8%	3.2%
… spending money got you or your family into trouble?	Reckless spending	28.3%	29.3%	1.9%

*Note*: “Affected” and “unaffected” refers to participants affected and unaffected by major depressive disorder (MDD) and/or an anxiety disorder. Each lifetime item is preceded with the question “Has there ever been a period of time when you were not your usual self and ….” Endorsement refers to the % of participants in the three analytical groups who endorsed the item. Participants who endorsed more than one  lifetime MDQ item were then presented with the question “You ticked ‘yes’ to more than one of the previous symptoms—have several of these ever happened during the same period of time?” The participants who answered “Yes” were subsequently presented with a list of their previously endorsed MDQ items and were asked to “select all that occurred during the same period of time.” Note the difference in *N* between concurrent and lifetime groups (participants were excluded from the concurrent item analysis if they reported that fewer than two MDQ items occurred in the same time period). The reduction in overall *N* for this analytical group means that some of the concurrent symptoms appear to have a higher endorsement than the lifetime symptoms.

For analyses of lifetime manic symptoms, GLAD and COPING NBR participants with complete data on all MDQ items were retained for analyses. For analyses of concurrent manic symptoms, only GLAD participants who reported more than one concurrent symptom and had complete data on all items were included in analyses. Data on concurrent manic symptoms were not available in COPING NBR.

See the Supplementary Methods for more information on how the MDQ screener was constructed.

#### 2.4.2 Quantitative manic symptom phenotypes

The number of concurrent manic symptoms endorsed by participants affected by MDD and/or an anxiety disorder were summed. This quantitative phenotype represents the total number of MDQ items that a participant self‐reported having experienced during one time period. Additionally, the number of endorsed lifetime manic symptoms were summed. This quantitative phenotype represents the total number of MDQ items that a participant self‐reported having experienced during their lifetime.

#### 2.4.3 Manic symptom subgroups

To identify symptom subgroups in the MDQ, we performed factor analyses of the concurrent and lifetime MDQ items (details in Supplementary Methods).

We performed exploratory factor analysis (EFA) on 70% of each sample and selected the model with the best fit statistics. We then performed confirmatory factor analysis (CFA) on the remaining 30%. The CFA model was predefined to that identified by EFA which provided a more stringent test of model fit compared to EFA. Factor scores were computed for each factor in the best‐fitting model in the whole sample. Factor scores were transformed using a rank‐based inverse normal transformation and then standardized (mean = 0, standard deviation [*SD*] = 1). All factor analyses were performed in R (details in Supplementary Methods). We also performed factor analysis of lifetime MDQ items in COPING NBR participants who were unaffected by MDD and/or an anxiety disorder (Supplementary Material).

### 2.5 Validation of the Mood Disorder Questionnaire

#### 2.5.1 Phenotypic validation

First, to assess the validity of the MDQ in our study sample, we calculated its sensitivity, specificity, positive predictive value (PPV) and negative predictive value (NPV) based on the participants' self‐reported diagnoses of bipolar disorder by a professional. Note that these could only be calculated in GLAD participants. Second, based on the number of reported manic symptoms, we compared the mean reported items between participants who self‐reported a diagnosis of bipolar disorder and those who self‐reported no diagnosis. See Supplementary Methods for details on self‐reported bipolar disorder diagnosis and the MDQ as a screener.

#### 2.5.2 Genetic validation

We calculated the genetic correlation between the quantitative manic symptom phenotypes assessed via the MDQ with the largest GWAS of bipolar disorder from the Psychiatric Genomics Consortium (PGC) (*N*_cases_ = 41,917, *N*_controls_ = 371,549) (Mullins et al., 2021). In addition to bipolar disorder, we calculated genetic correlations with 34 other psychiatric and behavioral traits (Table S11). To do this, we first had to perform genome‐wide association studies (GWASs) of the quantitative manic symptom phenotypes and the symptom subgroups.

##### Genotyping, imputation, and quality control

All data from GLAD and COPING NBR were genotyped by ThermoFisher on the Affymetrix UK Biobank Axiom Array v1 and v2 across numerous genotyping batches. Genetic data for GLAD and COPING NBR cohorts were separately subjected to quality control (QC) using the same pipeline (Supplementary Methods). For our specific analyses, additional QC was carried out. This included removing genotyped SNPs if missingness >5%, minor allele frequency (MAF) <0.01, or Hardy Weinberg Equilibrium *p* < 10^−10^. SNPs imputed with low confidence (INFO <0.3) were also excluded. Individuals with missingness >5%, a mismatch between their self‐reported assigned sex at birth and genetic sex, or whose genetic sex could not be determined were excluded. Last, one participant of each pair of duplicated participants between the GLAD and COPING NBR cohorts was excluded.

##### Genome‐wide association studies (GWASs)

GWASs were conducted with a mixed linear model using REGENIE, which controls for between‐subject relatedness using whole‐genome regression (Mbatchou et al., 2021). We included the first ten ancestry principal components and genotyping batch as covariates (principal component analysis plots in Figure S18). We performed GWASs of the quantitative manic symptom phenotypes in participants who were affected by MDD and/or an anxiety disorder. First, we performed GWASs of the total number of concurrent manic symptoms and of the factor scores for each subgroup identified by the factor analysis in participants of European ancestries. Second, we performed GWASs of lifetime manic symptoms and of the factor scores for each subgroup identified by the factor analyses in participants of European ancestries.

##### SNP‐based heritability and genetic correlations

Linkage Disequilibrium Score Regression (LDSC) (Bulik‐Sullivan et al., 2015) was used to estimate the SNP‐based heritability ($h_{\mathrm{SNP}}^{2}$) of each manic symptom phenotype. The SNP‐based heritability estimates were statistically significant if their *p*‐value surpassed the Bonferroni‐corrected alpha of 0.006 ($\alpha =\frac{0.05}{8}$) which adjusted for the eight heritability estimates. LDSC was then used to calculate genetic correlations (*r*_g_) between each of the manic symptom phenotypes and the GWAS summary statistics of psychiatric and behavioral traits (Table S11) using the extended 1000 Genomes linkage disequilibrium LD scores. These traits were selected from our internal GWAS summary statistics database. We only included traits that were sufficiently powered (a heritability *z*‐score >4 and a mean chi‐square >1.02). To reduce the multiple testing burden, we selected the most well‐powered GWASs when more than one trait, or similar traits, were available. Summary statistics were munged using LDSC and a list of SNPs from the extended 1000 Genomes phase three reference panel (1000 Genomes Project Consortium, 2015).

Eight sets of 37 genetic correlations were computed for each manic symptom phenotype in total (bipolar disorder overall, type I, type II, and 34 additional traits). Within each set, the alpha value was adjusted to correct for multiple testing using the Bonferroni method, giving an alpha value of 0.001 ($\alpha =\frac{0.05}{37}$).

We also calculated inter‐genetic correlations between the manic symptom phenotypes. Genetic correlations were significant if the *p*‐value surpassed the Bonferroni‐adjusted alpha of 0.008 ($\alpha =\frac{0.05}{6}$) to correct for six sets of inter‐genetic correlations.

##### Differences between genetic correlations

We tested whether the genetics of the symptom subgroups were differentially associated with the genetics of other traits. For traits that were significantly genetically correlated with more than one subgroup and the overall sum score, we used a block‐jackknife to calculate the standard error of the difference between pairs of genetic correlations. In a pairwise fashion, we first compared each trait's genetic correlation with the overall sum score to that same trait's genetic correlation with each of the subgroups. Second, we compared each trait's genetic correlation with a particular subgroup to that same trait's genetic correlation with another subgroup. Genetic correlations were significantly different to each other if the block‐jackknife *p*‐value surpassed the Bonferroni‐adjusted alpha ($\alpha =\frac{0.05}{15}$). The block‐jackknife method applied to genetic correlations has been described elsewhere (Mundy et al., 2021).

## 3 RESULTS

### 3.1 Study sample

A total of 52,108 GLAD and COPING NBR participants met criteria for MDD or any anxiety disorder. A total of 6308 COPING NBR participants did not meet criteria for MDD and any anxiety disorder and 13,195 were excluded from analyses for not having complete data needed to determine lifetime MDD and anxiety disorder diagnoses.

### 3.2 Manic symptoms

Quantitative phenotypes were derived from answers to questions in the MDQ about the symptoms alone. A total of 47,787 participants with mood or anxiety disorders (*N* excluded = 4,321) and 6,119 unaffected participants (*N* excluded = 189) had complete data for all 13 lifetime MDQ items. A total of 30,342 GLAD participants had complete data for all 13 concurrent MDQ items and endorsed more than one (flow‐chart in Figure S28). After inspecting a correlation matrix, the item “more active” had a correlation >0.8 with the item “more energy” (Figures S1–S3). The item “more active” was removed due to the problems associated with including highly collinear items in factor analysis (Flora et al., 2012). After the removal of this item, the total number of concurrent manic symptoms ranged 2–12 and the total number of lifetime manic symptoms ranged 0–12. The *N* included in the concurrent items analysis dropped to 29,899 after we removed anyone whose sum score was equal to one after the removal of “more active.”

**FIGURE 1:**
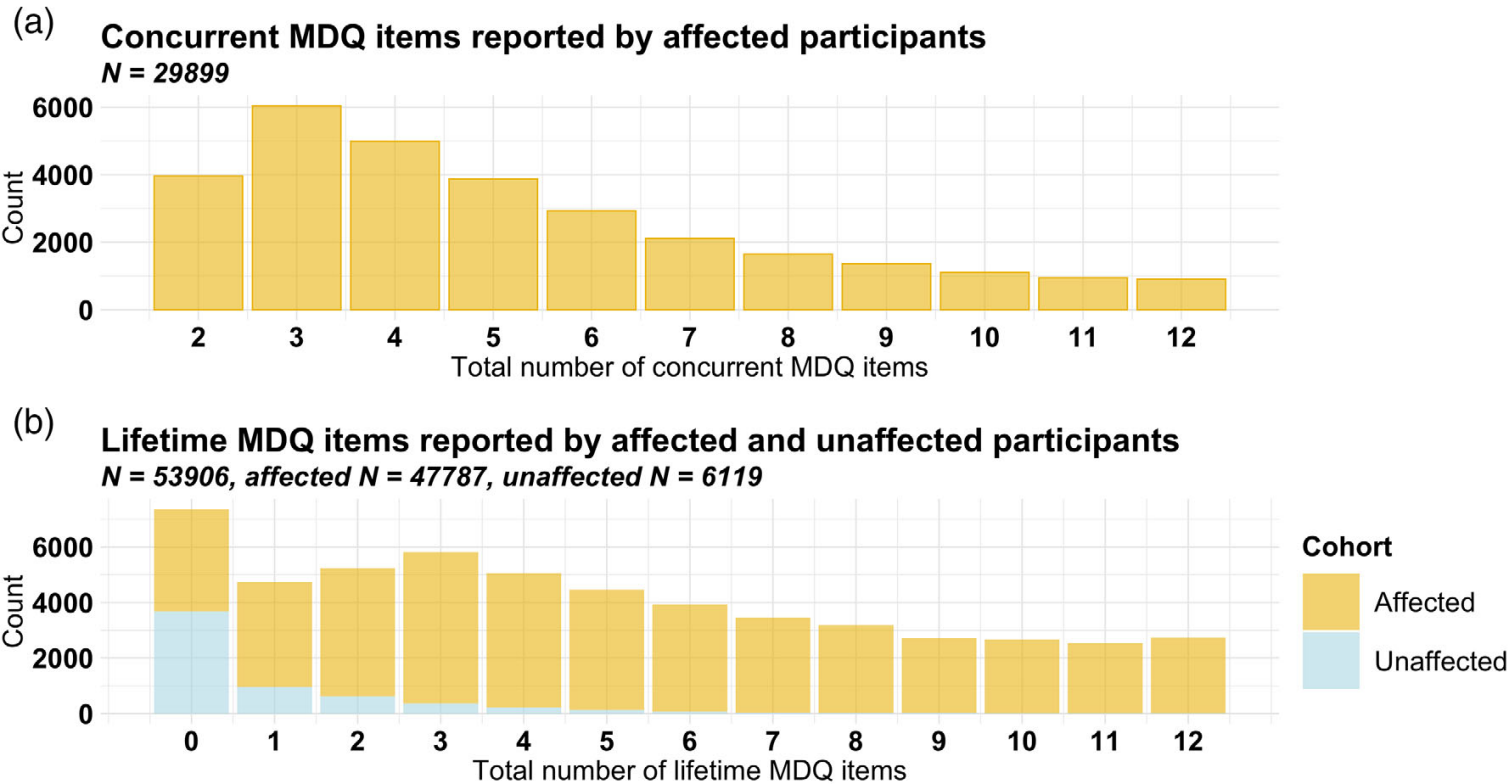
Distribution of Mood Disorder Questionnaire scores. (a) Distribution of the total number of concurrent manic symptoms reported by participants affected by major depressive disorder (MDD) and/or and anxiety disorder in the Mood Disorder Questionnaire (MDQ). (b) Distribution of the total number of lifetime manic symptoms reported by participants affected (yellow) and unaffected (blue) by major depressive disorder (MDD) and/or and anxiety disorder in the MDQ. Binary MDQ items (1 = “Yes,” 0 = “No”) were summed to create a quantitative sum score. The data originally included 13 items but one of a pair of highly correlated items was removed. Therefore, concurrent items ranged 2–12 and the lifetime items ranged 0–12.

The mean number of concurrent manic symptoms in participants affected by MDD and/or an anxiety disorder was 5.21 (*SD* = 2.70). The mean number of lifetime manic symptoms was 5.30 (*SD* = 3.50) in participants affected by MDD and/or an anxiety disorder and 0.97 (*SD* = 1.63) in unaffected participants. Demographic information for the three study samples for the quantitative manic symptom phenotypes and factor analysis are presented in Table 2, and the distributions of the number of endorsed MDQ items are presented in Figure 1.

**TABLE 2:** Demographic information for participants included in the three sets of analyses of the Mood Disorder Questionnaire (MDQ).

	Concurrent MDQ items in affected participants	Lifetime MDQ items in affected participants	Lifetime MDQ items in unaffected participants
Variable	*N* = 29,899^a^	*N* = 47,787^a^	*N* = 6119^a^
MDQ sum score	5.21 (2.70)	5.3 (3.5)	0.97 (1.63)
Age [years]	37 (14)	39 (15)	58 (13)
Sex			
Male	6117 (20%)	10,143 (21%)	3049 (50%)
Female	23,782 (80%)	37,644 (79%)	3070 (50%)
Ethnicity			
White	28,106 (94%)	44,900 (95%)	5773 (99%)
Mixed	826 (2.8%)	1119 (2.4%)	16 (0.3%)
Asian or Asian British	427 (1.4%)	668 (1.4%)	45 (0.8%)
Black or Black British	156 (0.5%)	240 (0.5%)	14 (0.2%)
Arab	24 (<0.1%)	43 (<0.1%)	
Other	289 (1.0%)	429 (0.9%)	
Missing	71	388	271
Education			
No university degree	15,036 (51%)	22,049 (47%)	2817 (47%)
University degree	14,479 (49%)	25,046 (53%)	3160 (53%)
Missing	384	692	142

*Note*: From left to right these are: (left), concurrent assessed manic symptoms in participants affected by major depressive disorder (MDD) and/or an anxiety disorder [range 2–12]; (middle), lifetime manic symptoms in participants affected by MDD and/or an anxiety disorder [range 0–12]; and (right), lifetime manic symptoms in participants unaffected by MDD and/or an anxiety disorder [range 0–12].

^a^ Mean (*SD*); *n* (%).

#### 3.2.1 Manic symptoms and self‐reported bipolar disorder diagnosis

Among 27,751 participants with complete data on concurrent MDQ items (range 2–12) and data on bipolar disorder diagnosis status, 2,464 (9%) self‐reported a diagnosis. The mean number of concurrent symptoms reported by participants with a diagnosis was 8.35 (*SD* = 2.86). The mean number reported by participants without a diagnosis was 4.82 (*SD* = 2.44) (*t* = −59.155, *p* < 2.2 × 10^−16^).

Among 34,653 participants with complete data on lifetime MDQ items (range 0–12) and data on bipolar disorder diagnosis status, 2,614 (8%) self‐reported a diagnosis. The mean number of lifetime symptoms reported by participants with a diagnosis was 10.62 (*SD* = 2.83). The mean number reported by participants without a diagnosis was 6.35 (*SD* = 3.28) (*t* = −73.101, *p*‐value<2.2 × 10^−16^). Descriptive statistics for the quantitative MDQ phenotypes in participants with and without a self‐reported diagnosis of bipolar disorder are presented in Table S1.

### 3.3 Manic symptom subgroups

Manic symptom subgroups were identified with factor analyses. See Figure 2 for a simplified diagram of the best fitting models. The item loadings for all models are presented in Figures S4‐S15, and fit statistics are presented in the Supplementary Results.

#### 3.3.1 Concurrent manic symptom subgroups

**FIGURE 2:**
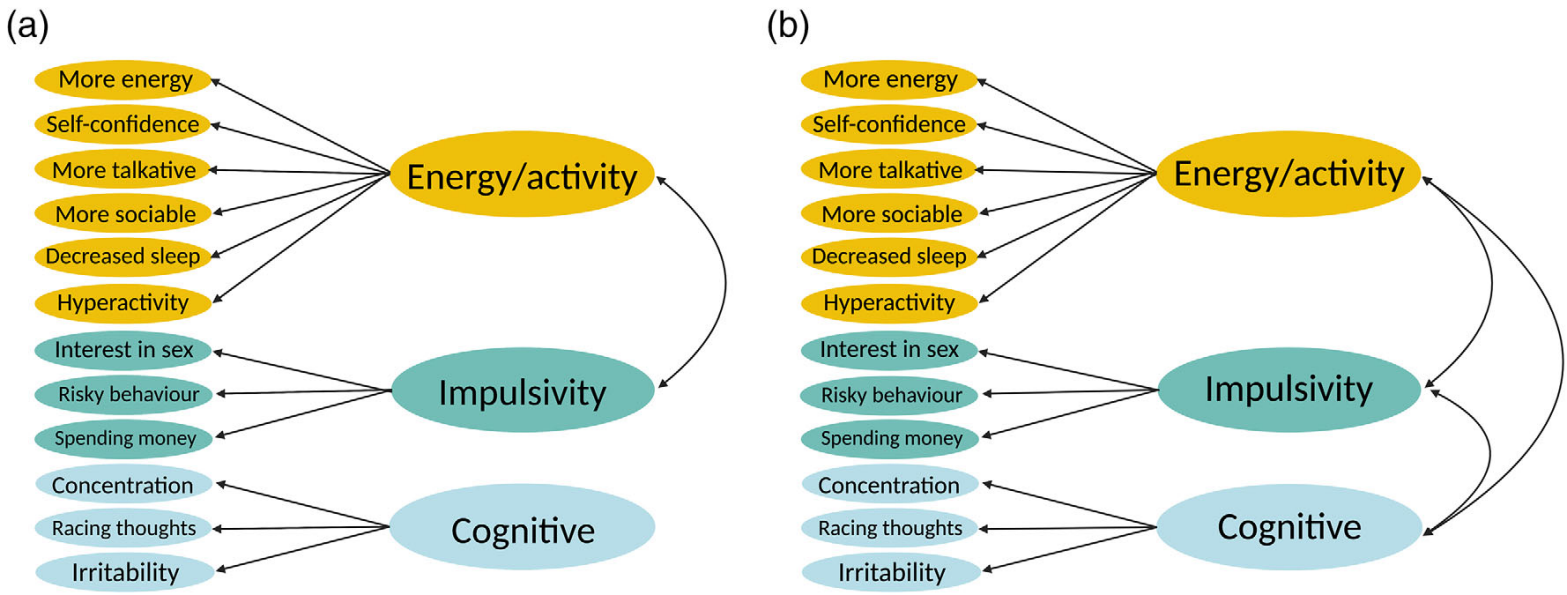
Factor analysis models of the Mood Disorder Questionnaire. (a) Simplified diagram of the best‐fitting model identified by the exploratory factor analysis (EFA) of 12 concurrent manic symptoms reported by participants by major depressive disorder (MDD) and/or an anxiety disorder in the Mood Disorder Questionnaire (MDQ). The model was confirmed with confirmatory factor analysis (CFA). EFA *N* = 20,929, CFA *N* = 8970. (b) Best‐fitting model identified by EFA of 12 lifetime manic symptoms reported by participants affected by MDD and/or an anxiety disorder in the MDQ. Model was confirmed with CFA. EFA *N* = 33,450, CFA *N* = 14,337. Item loadings are presented in Figures S2 and S5 and the correlations between the factors are presented in Tables S4b and S7b.

A total of 29,899 participants affected by MDD and/or an anxiety disorder were included in the factor analysis of concurrent MDQ items. Despite the scale's Cronbach's alpha being sufficient (Table S2), results showed considerable evidence that the MDQ items, when measured concurrently, lacked internal consistency. Notably, there was a distinct pattern in the item‐level correlations indicating that the items “concentration difficulties,” “racing thoughts,” and “irritability” did not correlate with other items (Figure S2) (details in Supplementary Results). The 12 concurrent symptoms loaded onto three factors: *energy/activity*, *impulsivity*, and *cognitive*. The *energy/activity* and *impulsivity* factors correlated (*r* = 0.54), but neither correlated with the *cognitive* factor (Figure 2; Table S4b). The model was confirmed in CFA on the remaining 30% of the sample (*N* = 8970) and showed good fit statistics (Table S5) (details in Supplementary Results).

#### 3.3.2 Lifetime manic symptom subgroups

A total of 47,787 participants affected by MDD and/or an anxiety disorder were included in the factor analysis of lifetime MDQ items. After performing EFA in 70% of the sample (*N* = 33,450) on 12 items, a three‐factor solution was selected as the final model because it had good fit statistics while retaining at least three items per factor (Table S7).

These three lifetime factors perfectly mirrored those identified in the concurrent analysis and were named accordingly (*energy/activity, cognitive*, and *impulsivity*). However, unlike the concurrent symptoms, the three subgroups correlated with each other (*r ≥* 0.55) (Figure 2; Figure S10; Table S7b). The model was confirmed in CFA on the remaining 30% of the sample (*N* = 14,337) and showed good fit statistics (Table S8) (details in Supplementary Results).

These results confirmed that the removal of “more active” from each analysis was justified. From inspecting the correlation matrices, it is clear that “more active” would have loaded onto the *energy/activity*, the largest factor, if it has been included. Therefore, even with this item removed, energy and activity levels are well assessed by the MDQ.

### 3.4 Validation of the Mood Disorder Questionnaire

#### 3.4.1 Phenotypic validation

A total of 34,479 GLAD participants had complete data on their bipolar disorder diagnosis status and the MDQ screener. Using a cut‐off at ≥7 concurrent manic symptoms, the sensitivity of the MDQ screener was 0.58, the specificity was 0.89, the PPV was 0.29, and the NPV was 0.96.

#### 3.4.2 Genetic validation

For the GWASs of concurrent MDQ‐assessed manic symptoms, a total of 11,568 participants of European ancestries had genetic data available which passed the genotype and imputed data QC. The SNP‐based heritability estimates from LDSC for the four concurrent manic symptom phenotypes ranged 3.8%–6.8% but none were significantly different to zero (Table S16).

For the GWASs of lifetime MDQ‐assessed manic symptoms, a total of 19,859 participants of European ancestries had available genetic data that passed the genotype and imputed data QC. The SNP‐based heritability estimates for the four lifetime manic symptom phenotypes ranged 5.1%–7.6% and all were significantly different to zero (*p* < 0.006) (Table S16). Manhattan plots and quantile–quantile (QQ) plots, produced by the functional annotation and mapping software FUMA can be found in Figures S19–S26 (Watanabe et al., 2017). We found that no SNPs reached genome‐wide significance (*p* < 5 × 10^−8^) in any of the GWASs.

##### Genetic correlations with bipolar disorder

Against our hypothesis, we found weak genetic correlations between both of the quantitative manic symptom phenotypes (concurrent or lifetime) and bipolar disorder overall, type I, and type II. None of these genetic correlations were significantly different to zero (Tables S14 and S15).

##### Genetic correlations with other traits

As expected by the SNP‐based heritability estimates (Table S16), there was far stronger evidence of genetic influences on the lifetime symptoms compared to the concurrent symptoms. The concurrent manic symptom sum score and its three symptom subgroups were not genetically correlated with any of the psychiatric or behavioral traits (Table S14).

**FIGURE 3:**
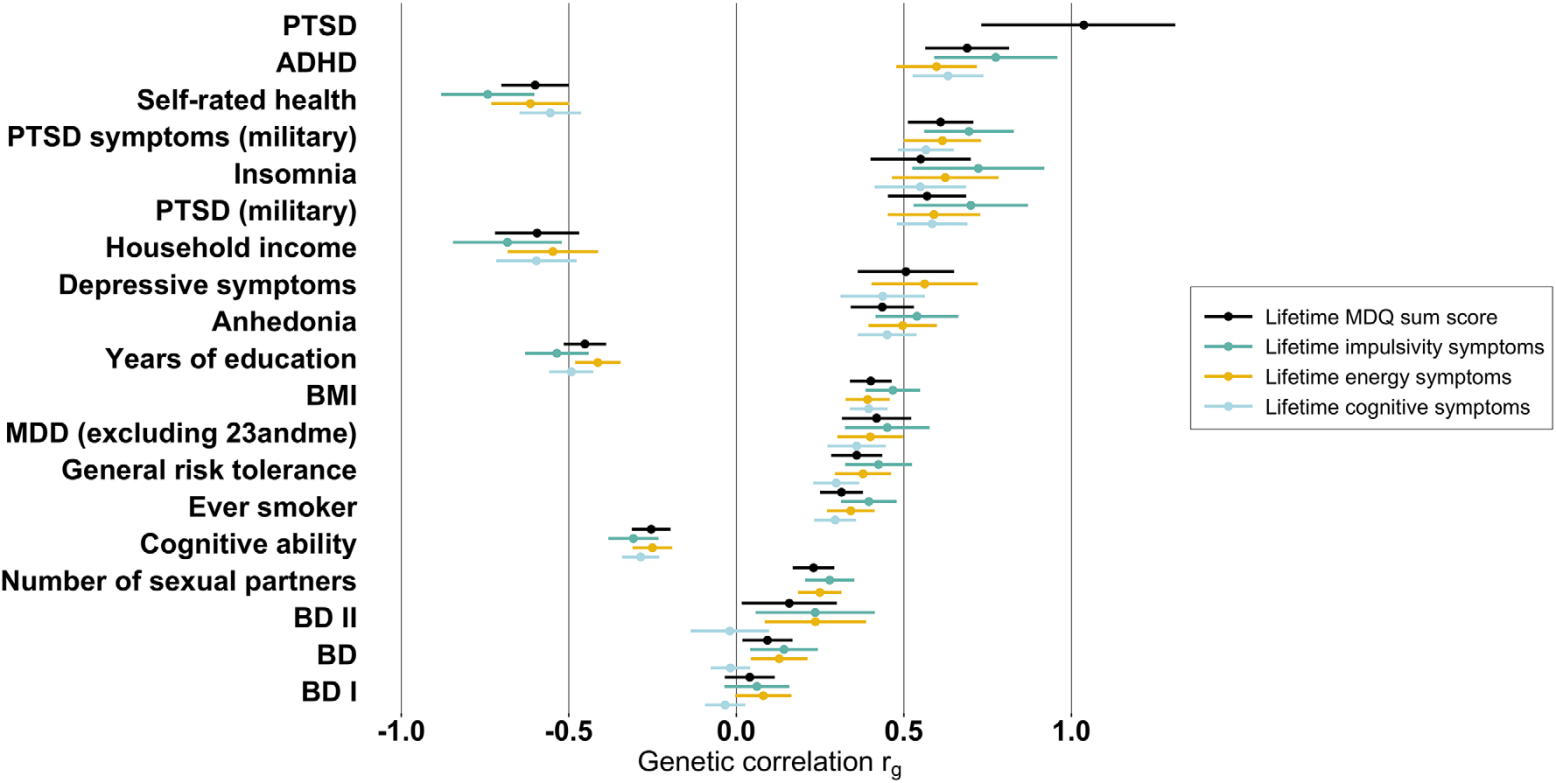
Significant genetic correlations. Genetic correlations were computed by Linkage Disequilibrium Score Regression (LDSC; see methods). Genetic correlations, indicated by dots with standard errors indicated by the lines either side of each estimate, were calculated between genome‐wide association study (GWAS) summary statistics of lifetime Mood Disorder Questionnaire (MDQ) phenotypes and GWAS summary statistics of psychiatric and behavioral traits. All genetic correlations presented here, apart from bipolar disorder overall, type I, and type II, were significant after correcting for multiple testing (*p* < 0.001) (bipolar disorder is included for comparison only). PTSD (military) and PTSD symptoms (military) refer to two GWASs of United States military subjects from the Million Veteran Program (MVP). PTSD refers to a GWAS of PTSD from the Psychiatric Genomics Consortium; PGC2). MDD refers to a GWAS of MDD from the PGC; PGC2 excluding 23andMe. Information about all the summary statistics used in our analysis, including the original publication and *N*, can be found in Table S11. Note that genetic correlations in LDSC are not bound to −1 or 1 due to sampling variation. ADHD, attention deficit hyperactivity disorder; BD I, bipolar disorder type 1; BD II, bipolar disorder type II; BD, bipolar disorder; BMI, body mass index; MDD, major depressive disorder; PTSD, posttraumatic stress disorder.

Contrastingly, we found significant genetic correlations between the lifetime manic symptom phenotypes and 16 psychiatric and behavioral traits (Figure 3). The highest genetic correlation was between the overall sum score and PTSD (*r*_g_ = 1.04, *p* = 0.0007). The symptom subgroups were not significantly correlated with PTSD (although their point estimates were similar to the sum score with *p*‐values just below significance; Table S14).

##### Differences between genetic correlations

Of the 16 traits that had a significant genetic correlation (*p* < 0.001) with at least one of the lifetime manic symptom phenotypes, 15 were significantly genetically correlated with more than one symptom subgroup as well as the overall sum score. These were PTSD (military), PTSD symptoms (military), self‐rated health, ADHD, insomnia, household income, depressive symptoms, ever smoker, years of education, anhedonia, MDD, BMI, general risk tolerance, number of sexual partners, and cognitive ability (Table S15). We carried forward these 15 traits to a block‐jackknife to test for significant differences (a) between each subgroup and the overall sum score and (b) between the subgroups themselves.

Genetic correlations were significantly different to each other if the block‐jackknife *p*‐value surpassed the Bonferroni‐adjusted alpha of 0.003 ($\alpha =\frac{0.05}{15}$). When compared to the traits' genetic correlations with the overall manic symptom sum score, none of the genetic correlations with the symptom subgroups differed significantly. Likewise, when compared to the traits' genetic correlations with other symptom subgroups, none of the genetic correlations differed significantly (Table S17).

##### Genetic correlations between manic symptom phenotypes

The concurrent manic symptom sum score was significantly genetically correlated with the *concurrent energy/activity factor* (*r*_g_ = 0.93, *p* = 4.20 × 10^−29^) and the *concurrent impulsivity factor* (*r*_g_ = 0.89, *p* = 2.47 × 10^−18^) but not with the *concurrent cognitive factor* (*r*_g_ = −0.60, *p* = 0.21).

Mirroring the phenotypic correlations between the symptom subgroups (Table S4b), the *concurrent energy/activity factor* and *concurrent impulsivity factor* were significantly genetically correlated (*r*_g_ = 0.89, *p* = 1.68 × 10^−18^), but the *concurrent cognitive factor* was not significantly genetically correlated with the *concurrent energy/activity factor* (*r*_g_ = −0.66, *p* = 0.04) or *concurrent impulsivity factor* (*r*_g_ = −0.40, *p* = 0.42) (Table S12).

The lifetime manic symptoms sum score was significantly genetically correlated with all three of its symptom subgroups (with *energy/activity r*_g_ = 1.00, *p* < 0.008; with *cognitive r*_g_ = 1.01, *p* < 0.008; with *impulsivity r*_g_ = 1.02, *p* < 0.008) (Table S13).

Reflected by the phenotypic correlations between the symptom subgroups (Figure S4), the lifetime symptom subgroups were all significantly genetically correlated with each other in a positive direction (*energy/activity* with *cognitive r*_g_ = 0.97, *p* = 7.64 × 10^−223^; *energy/activity* with *impulsivity r*_g_ = 1.02, *p* < 0.008; *cognitive* with *impulsivity r*_g_ = 1.03, *p* = 1.30 × 10^−175^) (Table S13).

## 4 DISCUSSION

We assessed the validity of the MDQ as a screening tool for bipolar disorder in a large sample of individuals affected by mental health problems, which is the population that the MDQ was designed for use in. Taking into account the number, co‐occurrence, severity, and duration of symptoms, the MDQ screener (using a cut‐off of ≥7 items, as suggested by the MDQ developers; Hirschfeld et al., 2000, 2003) showed mediocre sensitivity (0.58) and high specificity (0.89). The PPV was very poor (0.29). Our results showed that the MDQ may comprise three factors: *energy/activity*, *impulsivity*, and *cognitive*. When measured as concurrent symptoms, the MDQ items showed poor internal consistency with the *cognitive factor* not correlating with the *energy/activity* or the *impulsivity factor*. In our genetic analyses, the quantitative concurrent MDQ items were not significantly heritable. When examining lifetime experience of the MDQ items (i.e., not specifying concurrence), the quantitative score and the three factors showed weak but significant SNP‐based heritability. Lifetime MDQ items were genetically correlated with 16 other phenotypes, with the strongest correlation being with PTSD. An unexpected finding was the absence of significant genetic correlation with bipolar disorder overall, type I, and type II.

Very few studies have investigated the latent factor structure of the MDQ (Martino et al., 2020). One previous study found two latent factors: *energized‐activity* and *irritability‐racing thoughts* (Benazzi & Akiskal, 2003). They reported a dual factor structure to the MDQ (although they only included six of the items), whereas we found that both the concurrent and lifetime comprised three factors: *energy/activity, cognitive*, and *impulsivity*. In terms of similarities, the items that loaded onto their *irritability‐racing thoughts* factor were the same as the items that loaded onto our *cognitive* factor (“irritability,” “racing thoughts,” “concentration difficulties”). Likewise, their *energized‐activity* factor contained three items (“more active,” “more energy,” and “decreased sleep”) that also loaded onto our *energy/activity* factor. However, we found that four additional items loaded onto this factor (“more sociable,” “more self‐confidence,” “hyperactivity,” and “more talkative”).

The concurrent items showed poor internal consistency. An unexpected observation was that the items in the *concurrent cognitive* factor (“irritability,” “concentration difficulties,” and “racing thoughts”) did not correlate with the other two factors (Figure 2). This separation from the other items was also found when we performed a one‐factor EFA to check that all the items represented a unified latent construct. Here, the items in the *cognitive* factor did not load onto the single factor along with the other items (Table S3; Figure S4). This was also reflected in the genetic results. This is troubling given that these items constitute of a validated scale (Hirschfeld et al., 2000).

One explanation for this finding is that irritable mood, racing thoughts, and problems concentrating do not coincide with the other manic symptoms assessed in the MDQ. An alternative explanation, given that it is unusual to have items in a psychometric scale that are not correlated with each other, concerns recall and memory bias. Potentially, the participants were able to accurately recall that they have experienced these types of psychiatric problems at some point during their lifetime but failed to recognize that they occurred at the same time as the other symptoms. This would explain why these three concurrent MDQ items were not correlated with all remaining items and branched off as their own independent subgroup (Figure 2). The characteristics of the study sample may also play a role. The experiences asked about in the “irritability,” “racing thoughts,” and “concentration difficulties” items are common features of both anxiety and depression (Faravelli et al., 2012; Vidal‐Ribas et al., 2016). Of note is the fact that they were the most commonly endorsed symptoms (Table 1). It is likely that these symptoms were not specific to mania in this study sample. This may explain why they were generally not reported alongside other symptoms.

We found that the genetics of the items in the MDQ, measured continuously either as concurrent or cumulative lifetime symptoms, were not genetically correlated with bipolar disorder overall, type I, or type II. This was contrary to our hypothesis that all of the quantitative MDQ‐assessed manic symptom phenotypes would show significant, positive genetic correlations with bipolar disorder. We anticipated that the effect size would be larger for concurrent items since co‐occurrence of hypomanic or manic symptoms within the same four days or week is a requirement for DSM‐5 diagnosis (American Psychiatric Association, 2013) and a positive screen in the MDQ (Hirschfeld et al., 2000). There are a number of possible explanations for this unexpected finding.

First, the quantitative phenotypes were made by summing the number of MDQ items that a participant reported (answer options were “Yes” or “No”). Therefore, the composite scores simply reflect the number of manic symptoms a person has experienced and do not capture any information about the severity or duration of the symptoms (these are separate questions in the MDQ). Comparing quantitative scores between those who do and do not self‐report a diagnosis of bipolar (Table S1) does seem to suggest that the number of reported MDQ items is relevant for bipolar disorder. However, in the absence of information about duration and severity, it is possible that these quantitative phenotypes do not reflect hypomania/mania experienced in bipolar disorder. The bipolar disorder GWASs used for genetic correlations were from the most recent PGC analysis, with cases that had clinically‐diagnosed bipolar disorder. The DSM‐5 stipulates that hypomanic or manic symptoms must be present for one week or four days for a diagnosis of bipolar type I or type II, respectively, to be given. For bipolar disorder type I, a diagnosis can only be made when the “*mood disturbance is sufficiently severe to cause marked impairment in social or occupational functioning*” (American Psychiatric Association, 2013). Therefore, the phenotype of the PGC GWAS relates not just to the number of symptoms but also their duration and associated impairment. By contrast, the GWASs performed in our study only captured the *number* of symptoms a participant had experienced. These phenotypes therefore tell us nothing about whether or not the participant had experienced any clinically‐relevant symptoms, subthreshold symptoms, or full hypomania or mania.

In support of this conclusion is the genetic correlation of 0.38 with depressive symptoms in the most recent PGC bipolar disorder GWAS (Mullins et al., 2021). Since bipolar disorder involves *both* depressive and manic episodes, we expected bipolar disorder to show a similarly high genetic correlation with manic symptoms in our study. A crucial difference between our quantitative mania scores and the depressive symptom score was that information about severity and duration were included in the latter; depressive symptoms were assessed with two items from the nine item Patient Health Questionnaire (PHQ9) and the answer options were “Not at all,” “Several days,” “More than half the days,” “Nearly everyday” (Okbay et al., 2016). By contrast, the individual MDQ items can be answered with “Yes” or “No,” while two separate questions measure duration and severity. Therefore, it is not straightforward to construct a quantitative hypomania/mania phenotype with the MDQ without applying the same severity and duration to all endorsed items. As the GLAD study continues to recruit participants, an avenue for future investigation is to repeat the GWAS of concurrent symptoms within those individuals for which they caused a “moderate” or “severe” impact. Currently, only ~6000 participants who reported concurrent symptoms also reported that they caused moderate or severe problems. This sample is too small for a GWAS. However, as sample sizes grow, it may be possible to assess whether the genetics of manic symptoms in this subgroup are more closely related to bipolar disorder than in the sample overall.

The second possible explanation for our lack of genetic correlation with bipolar disorder relates to the type of genomic methodology that we applied to the MDQ. GWASs are only able to capture additive genetic risk from the SNPs in the genotyping or imputation panel. Similar to our results, the study of the MDQ by Hosang et al. (2022) found that MDQ‐assessed hypomania was not significantly genetically correlated with bipolar disorder PRS, but they did find a positive and significant twin‐based genetic correlation (Hosang et al., 2022). A study by Mistry et al. (2019) reported a similar result. Hypomania, assessed via the Hypomania Checklist 32, was not significantly associated with bipolar disorder PRS (Mistry et al., 2019). Taken together with the results of our study, it may be the case that common variant influences on hypomania/mania are not the same as those influencing bipolar disorder. Other sources of genetic variation, such as rare variants, could drive shared genetic influences between the two but these would not be captured by GWAS or PRS methods.

The final possible explanation for the lack of positive genetic correlation with bipolar disorder is that the MDQ is not a valid measure of hypomania/mania in our study sample. Most studies reporting high sensitivity and specificity of the MDQ screener include participants with well‐established mood disorder diagnoses, who are stabilized, or undergoing treatment. Consequently, their insight into the clinical utility of the MDQ for classifying bipolar disorder among outpatients, or those presenting with a variety of psychiatric complaints and unknown diagnoses, is limited (Zimmerman et al., 2009). The few studies that have investigated this report sensitivity values of 46%–64% and specificity values of 65%–83% (Gervasoni et al., 2009; Hardoy et al., 2005; Konuk et al., 2007; Zimmerman et al., 2009). Therefore, nearly half of individuals with bipolar disorder, in the population that the MDQ was designed for, could screen negatively, and a significant proportion who do not have bipolar disorder could screen positively. In our study, the sensitivity of the MDQ as a screener was similar to these previous studies at 0.58, and the specificity was good at 0.89. The PPV was low at 0.29. This suggests that, while the MDQ performed well at identifying participants without bipolar disorder, it falsely identified lots of participants as having bipolar disorder when they had not self‐reported a diagnosis by a professional.

The MDQ has poorer accuracy in identifying bipolar type II compared to type I (Gervasoni et al., 2009; Hardoy et al., 2005; Hirschfeld et al., 2000; Zimmerman et al., 2009). This, combined with the fact that the MDQ performs more poorly in community samples compared to clinical samples (Miller et al., 2009), suggests that symptom severity is an important factor dictating the psychometric properties of the MDQ. Due to the characteristics of our study sample, some of the MDQ items were very highly endorsed (Table 1). This could be because, among individuals with MDD and/or anxiety, they ask about relatively common experiences rather than symptoms of hypomania/mania. This is supported by the finding that MDQ overestimated the prevalence of bipolar disorder in our study sample (PPV of 0.29). Overall, it is possible that the items in the MDQ do not capture hypomanic/manic symptoms with much precision. This may also be a factor influencing our genetic correlation results; lack of specificity to hypomania/mania could have led to noise in our phenotypes which, as a result, may have diluted the MDQ's genetic sharing with bipolar disorder.

Viewing our genetic correlation results overall, there is no obvious pattern. It appears that the MDQ items index many traits. Significant genetic correlations were found with MDD, depressive symptoms, insomnia, anhedonia, and PTSD symptoms, as well as with risk‐taking and smoking. Although the genetic correlation between lifetime MDQ items with PTSD was high, notably this was not confirmed by its phenotypic correlation with PTSD symptoms measured in the same participants (Supplementary Material). In Table 1, there is a notable difference in endorsement of the MDQ items between participants affected and unaffected by MDD and anxiety. One explanation for this is that a proportion of the affected participants have undiagnosed bipolar disorder and therefore report more of the MDQ items. However, given the psychometric properties of the MDQ screener in our study, a more likely explanation is that the MDQ items capture non‐specific aspects of mental illness. Combined with the genetic correlation results, this suggests that, among individuals with MDD and/or anxiety, the MDQ captures symptoms of general distress or psychopathology rather than mania specifically.

A strength of our study was that we were able to measure manic symptoms quantitatively. Previous GWASs that have isolated mania for genetic analyses have dealt with the phenotype as a binary variable which often incurs a loss of statistical power (Greenwood et al., 2013; Lee et al., 2013). Another strength was the measurement of two different types of manic symptoms: concurrent and lifetime symptoms. Our findings suggest that the internal consistency of concurrent MDQ items is compromised when applied to at‐risk populations, possibly due to recall and memory biases. The process of screening via the MDQ involves participants self‐reporting their symptoms. Our findings suggest that this may be inadequate, especially for individuals who present with symptoms of anxiety and/or depression, due to poor recall of their experience of “irritability,” “racing thoughts,” and “concentration difficulties.” This may have implications for the application of the MDQ as a bipolar disorder screener to these individuals.

Our conclusions should be considered in light of several limitations. First is the relatively modest size of our study sample for genetic analyses compared to modern GWAS standards. This may have impacted statistical power, especially in the GWASs of concurrent symptoms which already had attenuated power due to the items' poor internal consistency. Second, the criteria for the MDQ screener were based upon symptoms causing “moderate” or “severe” functional impairment (Hirschfeld et al., 2000, 2003). Therefore, it is possible that some individuals with bipolar disorder type II may have been missed. However, even with the possible under‐recognition of type II by the MDQ screener, it showed an overestimation of the number of individuals with bipolar disorder in our study sample. Third, the GLAD Study is a cohort with generally severe symptomatology, and we cannot generalize to individuals with milder forms of MDD and anxiety (Davies et al., 2019). These sample characteristics also meant that the SNP‐based heritability estimates were difficult to interpret, as they depend on the population in which the phenotype is measured. Since we did not use a general population sample, it is difficult to gauge what the SNP‐based heritability represents in our study. Another way to measure hypomania/mania's SNP‐based heritability would be to perform GWAS of bipolar disorder type I/type II vs. MDD. However, some eventually progress to bipolar disorder from MDD (Angst et al., 2005; Baryshnikov et al., 2020) which means that we may inadvertently include “hidden” individuals with bipolar disorder in the MDD comparison group. This would dilute the genetic signal of mania. This point relates to a final limitation that our assessment of bipolar disorder was based on self‐reports. Calculations of sensitivity, specificity, PPV, and NPV should be made against a “gold‐standard” reference (e.g., a diagnosis from a clinical interview). Given that bipolar disorder can go undiagnosed for up to ten years (Drancourt et al., 2013; Hirschfeld, Lewis, & Vornik, 2003; Mantere et al., 2004) and unipolar depression is the most likely misdiagnosis (Hirschfeld, Lewis, & Vornik, 2003) the eligibility criteria of the GLAD Study means that it is highly probable that a proportion of the participants have undiagnosed bipolar disorder. This may have contributed to the low PPV (0.29).

Overall, our study adds to existing literature questioning the MDQ's validity by showing that, among individuals with MDD and/or anxiety, the items alone capture dimensions of general psychopathology rather than hypomania/mania. Furthermore, our results question the concurrent items' internal consistency. Researchers using the MDQ to measure bipolar disorder in epidemiological studies or biobanks should be cautious of its ability to accurately index symptoms of hypomania/mania and should consider ways to incorporate symptom severity and duration into their phenotyping method.

## AUTHOR CONTRIBUTIONS

*Data curation*: Molly R. Davies, Gursharan Kalsi, Henry C. Rogers, Brett N. Adey, Sang Hyuck Lee, Jonathan R. I. Coleman, Andrew M. McIntosh, Matthew Hotopf, Thalia C. Eley, Gerome Breen. *Data cleaning*: Jessica Mundy, Helena L. Davies, Christopher Hübel, Molly R. Davies, Jonathan R. I. Coleman. *Formal analysis*: Jessica Mundy, Christopher Hübel, Evangelos Vassos, Gerome Breen. *Funding acquisition*: Robin M. Murray, Gursharan Kalsi, Matthew Hotopf, Thalia C. Eley, Gerome Breen. *Investigation*: Jessica Mundy, Christopher Hübel, Jonathan R. I. Coleman, Evangelos Vassos, Gerome Breen. *Methodology*: Jessica Mundy, Christopher Hübel, Jonathan R. I. Coleman, Evangelos Vassos, Gerome Breen. *Project administration*: Gursharan Kalsi, Christopher Hübel, Henry C. Rogers, Molly R. Davies, Gerome Breen. *Resources*: Molly R. Davies, Gursharan Kalsi, Henry C. Rogers, Gerome Breen. *Software*: Christopher Hübel, Brett N. Adey, Jonathan R. I. Coleman, Sang Hyuck Lee. *Supervision*: Robin Murray, Evangelos Vassos, Gerome Breen. *Visualization*: Jessica Mundy. *Writing—original draft*: Jessica Mundy. *Writing—review & editing*: Jessica Mundy, Helena L. Davies, Molly R. Davies, Brett N. Adey, Christopher Hübel, Matthew Hotopf, Thalia C. Eley, Robin M. Murray, Evangelos Vassos, Gerome Breen. *Preparation*: Jessica Mundy, Robin Murray, Evangelos Vassos, Gerome Breen.

## FUNDING INFORMATION

This work was supported by the National Institute for Health and Care Research (NIHR) BioResource (RG94028, RG85445), NIHR Biomedical Research Centre (IS‐BRC‐1215‐20018), HSC R&D Division, Public Health Agency (COM/5516/18), MRC Mental Health Data Pathfinder Award (MC_PC_17217), and the National Centre for Mental Health funding through Health and Care Research Wales. Jessica Mundy acknowledges funding from the Lord Leverhulme Charitable Grant. Helena L. Davies acknowledges funding from the Economic and Social Research Council (ESRC) as part of a PhD studentship. Brett N. Adey acknowledges funding through a pre‐doctoral Fellowship from the NIHR (NIHR301067). Dr. Christopher Hübel acknowledges funding from Lundbeckfonden (R276‐2018‐4581).

## CONFLICT OF INTEREST STATEMENT

Gerome Breen has received honoraria, research or conference grants and consulting fees from Illumina, Otsuka, and COMPASS Pathfinder Ltd. The remaining authors have nothing to disclose.

## Supporting information


**Figure S1.** Correlations between concurrent Mood Disorder Questionnaire (MDQ) items in individuals affected by major depressive disorder (MDD) and/or an anxiety disorder.
**Figure S2.** Correlations between lifetime Mood Disorder Questionnaire (MDQ) items in individuals affected by major depressive disorder (MDD) and/or an anxiety disorder.
**Figure S3.** Correlations between lifetime Mood Disorder Questionnaire (MDQ) items in individuals unaffected by major depressive disorder (MDD) and/or an anxiety disorder.
**Figure S4.** Exploratory factor analysis (EFA): one factor solution of 12 concurrent Mood Disorder Questionnaire (MDQ) items in affected participants.
**Figure S5.** Exploratory factor analysis (EFA): two factor solution of 12 concurrent Mood Disorder Questionnaire (MDQ) items in affected participants.
**Figure S6.** Exploratory factor analysis (EFA): three factor solution of 12 concurrent Mood Disorder Questionnaire (MDQ) items in affected participants.
**Figure S7.** Exploratory factor analysis (EFA): four factor solution of 12 concurrent Mood Disorder Questionnaire (MDQ) items in affected participants.
**Figure S8.** Exploratory factor analysis (EFA): one factor solution of 12 lifetime Mood Disorder Questionnaire (MDQ) items in affected participants.
**Figure S9.** Exploratory factor analysis (EFA): two factor solution of 12 lifetime Mood Disorder Questionnaire (MDQ) items in affected participants.
**Figure S10.** Exploratory factor analysis (EFA): three factor solution of 12 lifetime Mood Disorder Questionnaire (MDQ) items in affected participants.
**Figure S11.** Exploratory factor analysis (EFA): four factor solution of 12 lifetime Mood Disorder Questionnaire (MDQ) items in affected participants.
**Figure S12.** Exploratory factor analysis (EFA): one factor solution of 12 lifetime Mood Disorder Questionnaire (MDQ) items in unaffected participants.
**Figure S13.** Exploratory factor analysis (EFA): two factor solution of 12 lifetime Mood Disorder Questionnaire (MDQ) items in unaffected participants.
**Figure S14.** Exploratory factor analysis (EFA): three factor solution of 12 lifetime Mood Disorder Questionnaire (MDQ) items in unaffected participants.
**Figure S15.** Exploratory factor analysis (EFA): four factor solution of 12 lifetime Mood Disorder Questionnaire (MDQ) items in unaffected participants.
**Figure S16.** Raw factor scores from factor analysis of 12 concurrent manic symptoms measured by the Mood Disorder Questionnaire (MDQ) in affected participants.
**Figure S17.** Raw factor scores from factor analysis of 12 lifetime manic symptoms measured by the Mood Disorder Questionnaire (MDQ) in affected participants.
**Figure S18.** Principal component analysis (PCA) plots.
**Figure S19.** Quantile–quantile (QQ) plot and Manhattan plot of genome‐wide association study (GWAS) results of the concurrent manic symptom sum score measured by the Mood Disorder Questionnaire (MDQ) in affected participants of European ancestry (*N* = 11,568).
**Figure S20.** Quantile–quantile (QQ) plot and Manhattan plot of genome‐wide association study (GWAS) results of concurrent energy/activity factor measured by the Mood Disorder Questionnaire (MDQ) in affected participants of European ancestry (*N* = 11,568). *GWAS was performed with REGENIE covarying for the first 10 ancestry principal components and genotyping batch. Manhattan and QQ plots were produced using FUMA*.
**Figure S21.** Quantile–quantile (QQ) plot and Manhattan plot of the genome‐wide association study (GWAS) results of concurrent cognitive factor measured by the Mood Disorder Questionnaire (MDQ) in affected participants of European ancestry (*N* = 11,568). *GWAS was performed with REGENIE covarying for the first 10 ancestry principal components and genotyping batch. Manhattan and QQ plots were produced using FUMA*.
**Figure S22.** Quantile–quantile (QQ) plot and Manhattan plot of genome‐wide association study (GWAS) results of the concurrent impulsivity factor measured by the Mood Disorder Questionnaire (MDQ) in affected participants of European ancestry (*N* = 11,568). *GWAS was performed with REGENIE covarying for the first 10 ancestry principal components and genotyping batch. Manhattan and QQ plots were produced using FUMA*.
**Figure S23.** Quantile–quantile (QQ) plot and Manhattan plot of genome‐wide association study (GWAS) results of the lifetime manic symptom sum score measured by the Mood Disorder Questionnaire (MDQ) in affected participants of European ancestry (*N* = 19,859). *GWAS was performed with REGENIE covarying for the first 10 ancestry principal components and genotyping batch. Manhattan and QQ plots were produced using FUMA*.
**Figure S24.** Quantile–quantile (QQ) plot and Manhattan plot of genome‐wide association study (GWAS) results of the lifetime energy/activity factor measured by the Mood Disorder Questionnaire (MDQ) in affected participants of European ancestry (*N* = 19,859). *GWAS was performed with REGENIE covarying for the first 10 ancestry principal components and genotyping batch. Manhattan and QQ plots were produced using FUMA*.
**Figure S25.** Quantile–quantile (QQ) plot and Manhattan plot of genome‐wide association study (GWAS) results of the lifetime cognitive factor measured by the Mood Disorder Questionnaire (MDQ) in affected participants of European ancestry (*N* = 19,859).
**Figure S26.** Quantile–quantile (QQ) plot and Manhattan plot of genome‐wide association study (GWAS) results of the lifetime impulsivity factor measured by the Mood Disorder Questionnaire (MDQ) in affected participants of European ancestry (*N* = 19,859).
**Figure S27.** Scatter plot of affected participants' lifetime manic symptoms measured by the Mood Disorder Questionnaire (MDQ) and current posttraumatic stress disorder (PTSD) symptoms measured by the 6 item PTSD Checklist (PCL‐6). Lifetime manic symptoms were scored 0–12 and current PTSD symptoms were scored 6–30.
**Figure S28.** Flow‐chart detailing how Genetic Links to Anxiety and Depression (GLAD) Study and COVID‐19 Psychiatry and Neurological Genetics Study participants from the NIHR Bioresource (COPING NBR) were categorized as either “affected” or “unaffected” by major depressive disorder (MDD) and/or any anxiety disorder. MDQ = Mood Disorder Questionnaire.

**Data S1.** Supporting Information.

**Table S1.** Descriptive statistics of the quantitative manic symptom phenotypes (sum scores) derived from the Mood Disorder Questionnaire (MDQ) in participants with and without a self‐reported diagnosis of bipolar disorder (BD) by a professional.
**Table S2.** Statistics from testing assumptions for factor analysis of the Mood Disorder Questionnaire (MDQ) items. “Affected” refers to participants affected by major depressive disorder (MDD) and/or an anxiety disorder.
**Table S3.** Item loadings for all factor solutions for the exploratory factor analysis (EFA) of 12 concurrent manic symptoms derived from the Mood Disorder Questionnaire (MDQ) in affected participants. “concurrent more active” was previously removed due to having a correlation of 0.87 with “concurrent more energy.” EFA was performed with the psych R package. Oblimin rotation method was used to allow the latent factors to correlate with each other and the factoring method was “minimum residuals.”

## Data Availability

GLAD and COPING study data is available via a data request application to the NIHR BioResource (https://bioresource.nihr.ac.uk/using‐our‐bioresource/academic‐and‐clinical‐researchers/apply‐for‐bioresource‐data/). The data are not publicly available due to restrictions outlined in the study protocol and specified to participants during the consent process. A specific data freeze is available including the variables for the analyses described in this paper; email gladstudy@kcl.ac.uk for details. The summary statistics from the GWASs performed in this study are available by contacting Professor Gerome Breen.
