# Supplementary material for: Genetic examination of the Mood Disorder Questionnaire and its relationship with bipolar disorder
Source: Am J Med Genet B Neuropsychiatr Genet. 2023 May 13;192(7-8):147–60. doi: 10.1002/ajmg.b.32938 (PMC10952822; doi:10.1002/ajmg.b.32938)

**Genetic examination of the Mood Disorder Questionnaire and its relationship with bipolar disorder**

**Supplementary figures**

**Figure S1. Correlations between concurrent Mood Disorder Questionnaire (MDQ) items in individuals affected by major depressive disorder (MDD) and/or an anxiety disorder.**

*Tetrachoric correlation matrix of concurrent manic symptoms from answers to the Mood Disorder Questionnaire (MDQ) in participants affected by major depressive disorder (MDD) and/or an anxiety disorder (N=31,427). Participants can answer with “Yes” or “No” which were coded as 1 or 0 respectively. Correlation matrix was computed using the hetcor R package. Participants can answer with “Yes” or “No” which were coded as 1 or 0 respectively. Correlations are therefore tetrachoric (which is a special case of polychoric). “More active” was removed from analysis following inspection of the correlation matrix due to its correlation of 0.87 with “more energy”. Then, 83 participants were excluded from further analyses because they previously endorsed two concurrent items and now, after the removal of the item “more active”, only endorsed one concurrent item. This left a final N of 29,889.*

**
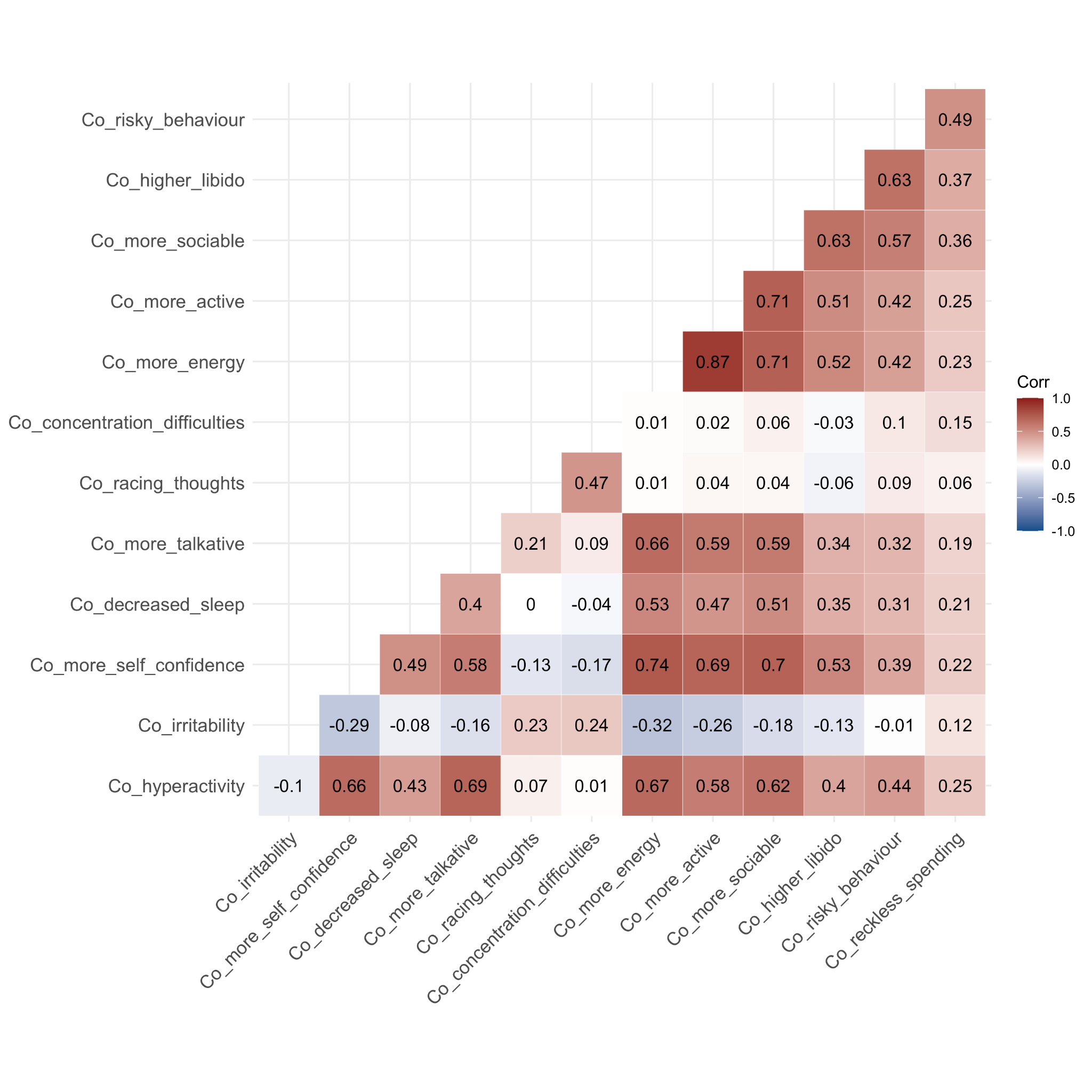
**

**Figure S2. Correlations between lifetime Mood Disorder Questionnaire (MDQ) items in individuals affected by major depressive disorder (MDD) and/or an anxiety disorder.**

*Tetrachoric correlation matrix of lifetime manic symptoms from answers to the Mood Disorder Questionnaire (MDQ) in participants affected by major depressive disorder (MDD) and/or an anxiety disorder (N=47,787). Participants can answer with “Yes” or “No” which were coded as 1 or 0 respectively. Correlation matrix was computed using the hetcor R package. Participants can answer with “Yes” or “No” which were coded as 1 or 0 respectively. Correlations are therefore tetrachoric (which is a special case of polychoric). “More active” was removed from analysis following inspection of the correlation matrix due to its correlation of 0.90 with “more energy”.*

***
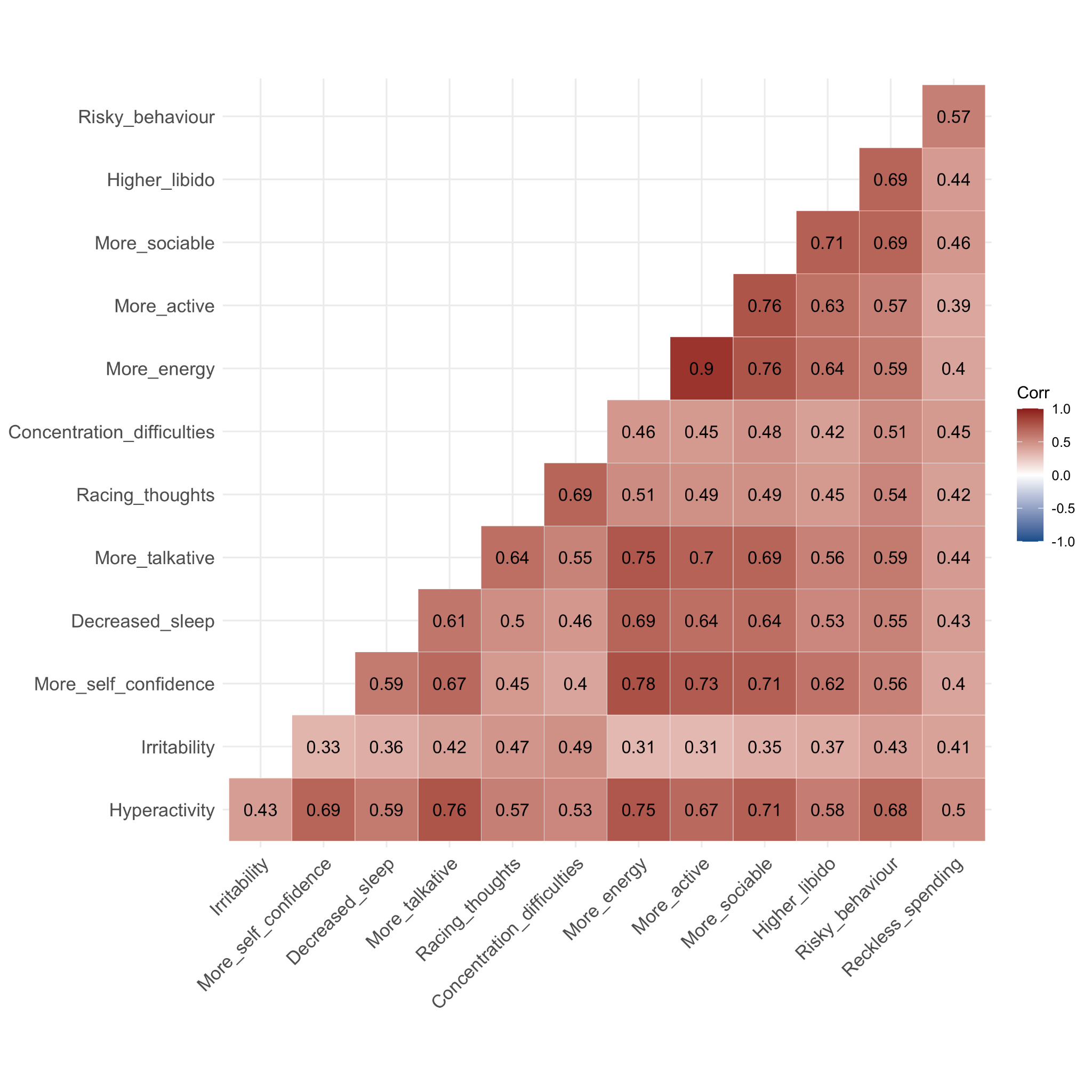
***

**Figure S3. Correlations between lifetime Mood Disorder Questionnaire (MDQ) items in individuals unaffected by major depressive disorder (MDD) and/or an anxiety disorder.**

*Tetrachoric correlation matrix of lifetime manic symptoms from answers to the Mood Disorder Questionnaire (MDQ) in participants unaffected by major depressive disorder (MDD) and/or an anxiety disorder (N=6,119). Correlation matrix was computed using the hetcor R package. Participants can answer with “Yes” or “No” which were coded as 1 or 0 respectively. Correlations are therefore tetrachoric (which is a special case of polychoric). “More active” was removed from analysis following inspection of the correlation matrix due to its correlation of 0.90 with “more energy”.*


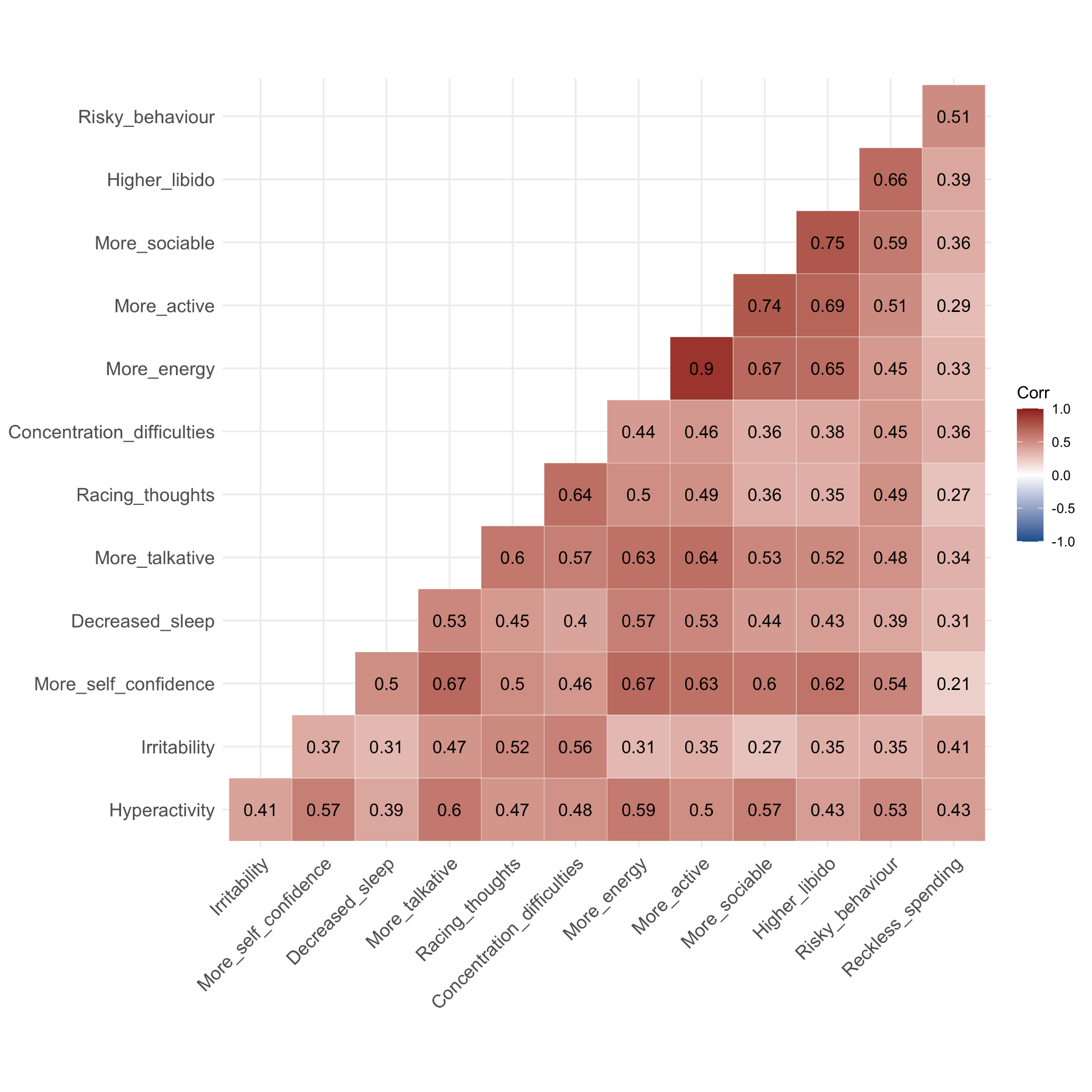


**Figure S4. Exploratory factor analysis (EFA): one factor solution of 12 concurrent Mood Disorder Questionnaire (MDQ) items in affected participants.**

*EFA was performed with the psych R package. Oblimin rotation method was used to allow the latent factors to correlate with each other and the factoring method was “minimum residuals”.*

**
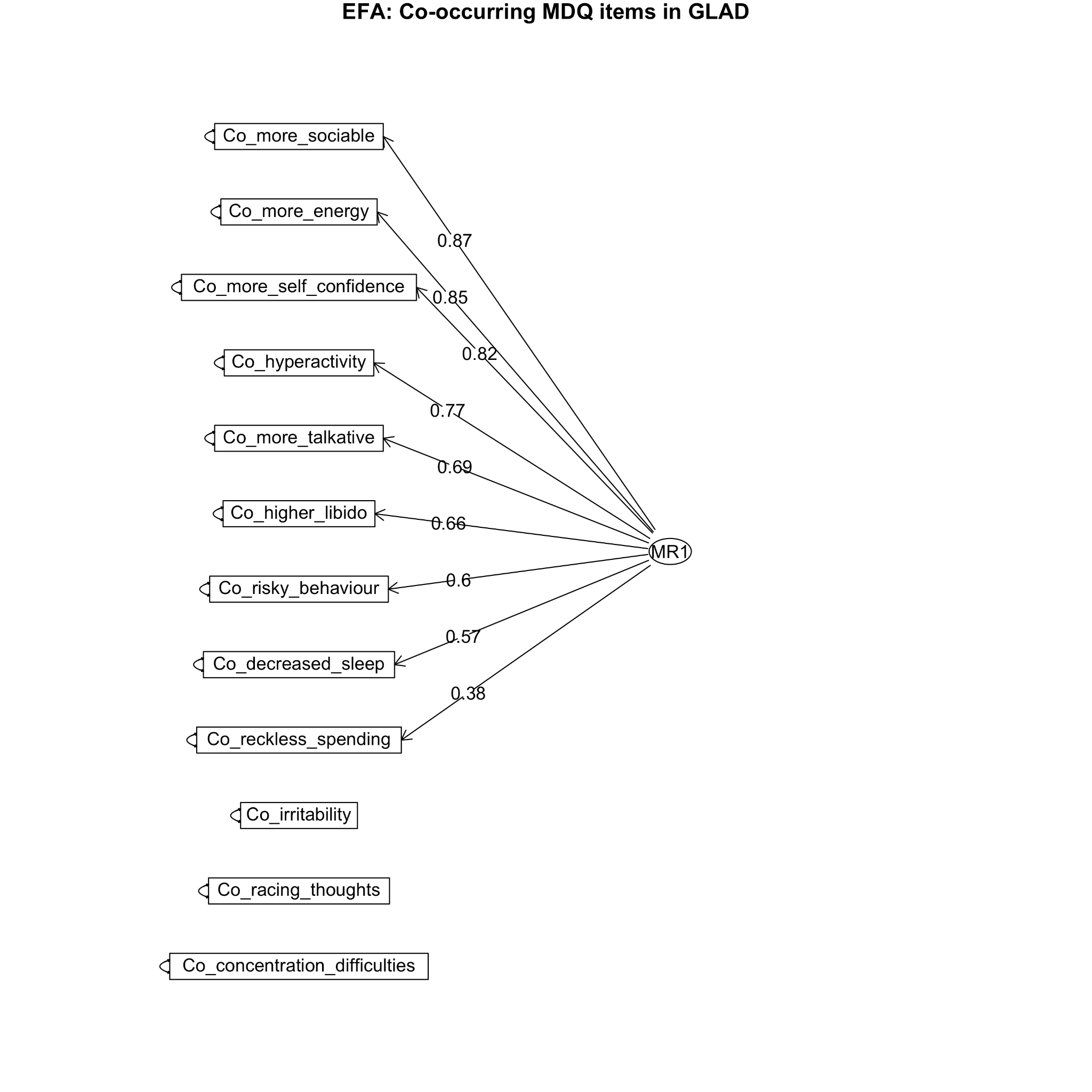
**

**Figure S5. Exploratory factor analysis (EFA): two factor solution of 12 concurrent Mood Disorder Questionnaire (MDQ) items in affected participants.**

*EFA was performed with the psych R package. Oblimin rotation method was used to allow the latent factors to correlate with each other and the factoring method was “minimum residuals”.*

**
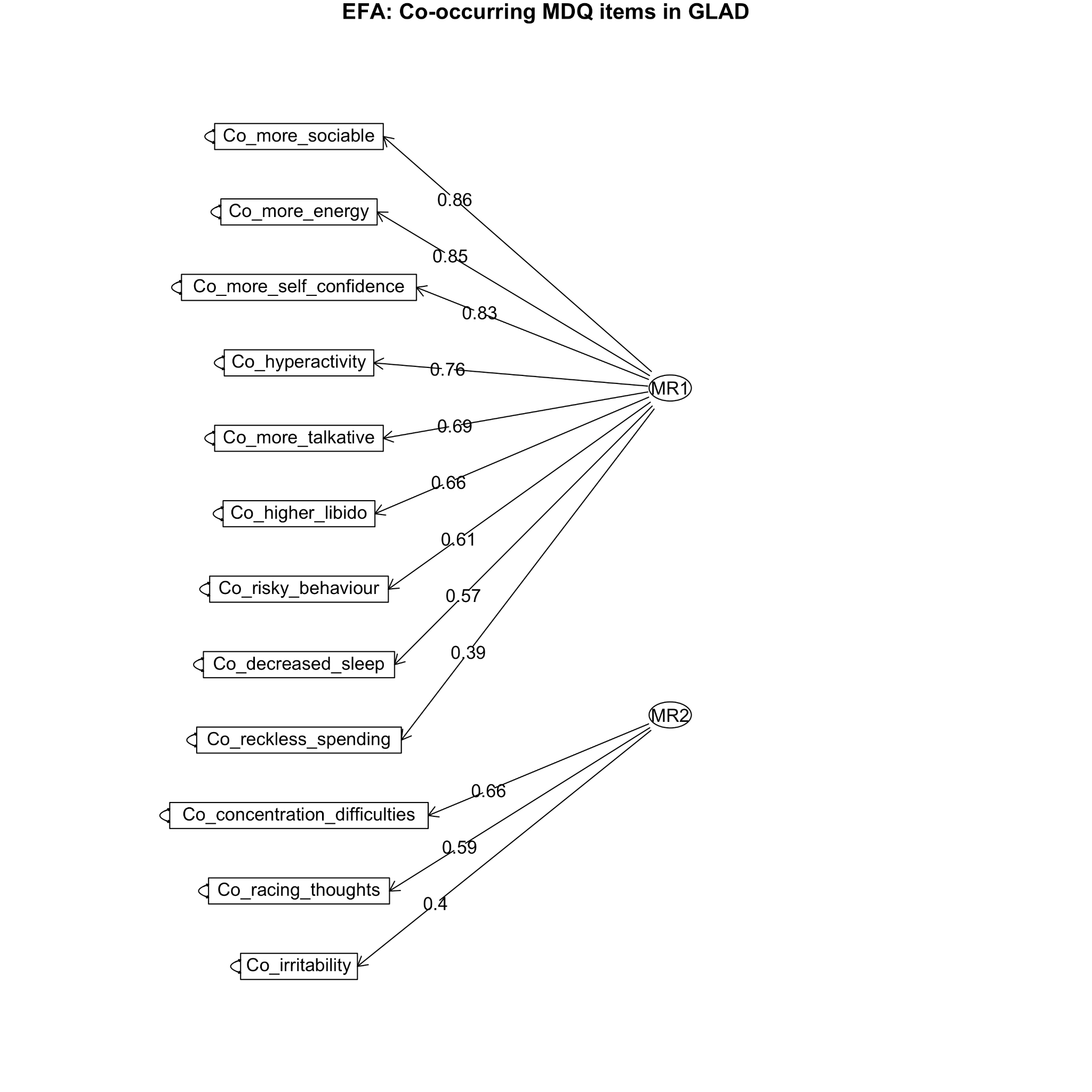
**

**Figure S6. Exploratory factor analysis (EFA): three factor solution of 12 concurrent Mood Disorder Questionnaire (MDQ) items in affected participants.**

*EFA was performed with the psych R package. Oblimin rotation method was used to allow the latent factors to correlate with each other and the factoring method was “minimum residuals”.*

**
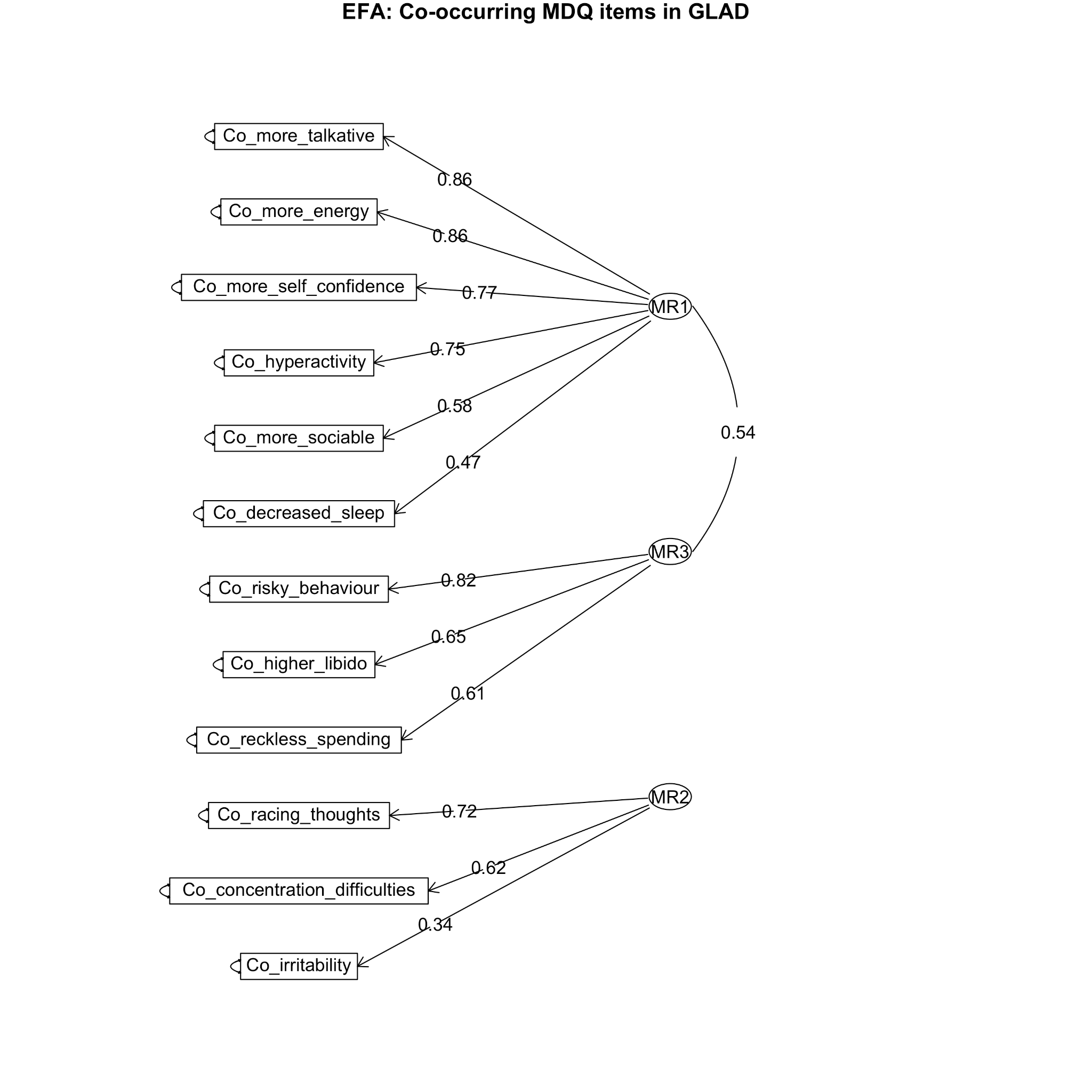
**

**Figure S7. Exploratory factor analysis (EFA): four factor solution of 12 concurrent Mood Disorder Questionnaire (MDQ) items in affected participants.**

*EFA was performed with the psych R package. Oblimin rotation method was used to allow the latent factors to correlate with each other and the factoring method was “minimum residuals”.*

**
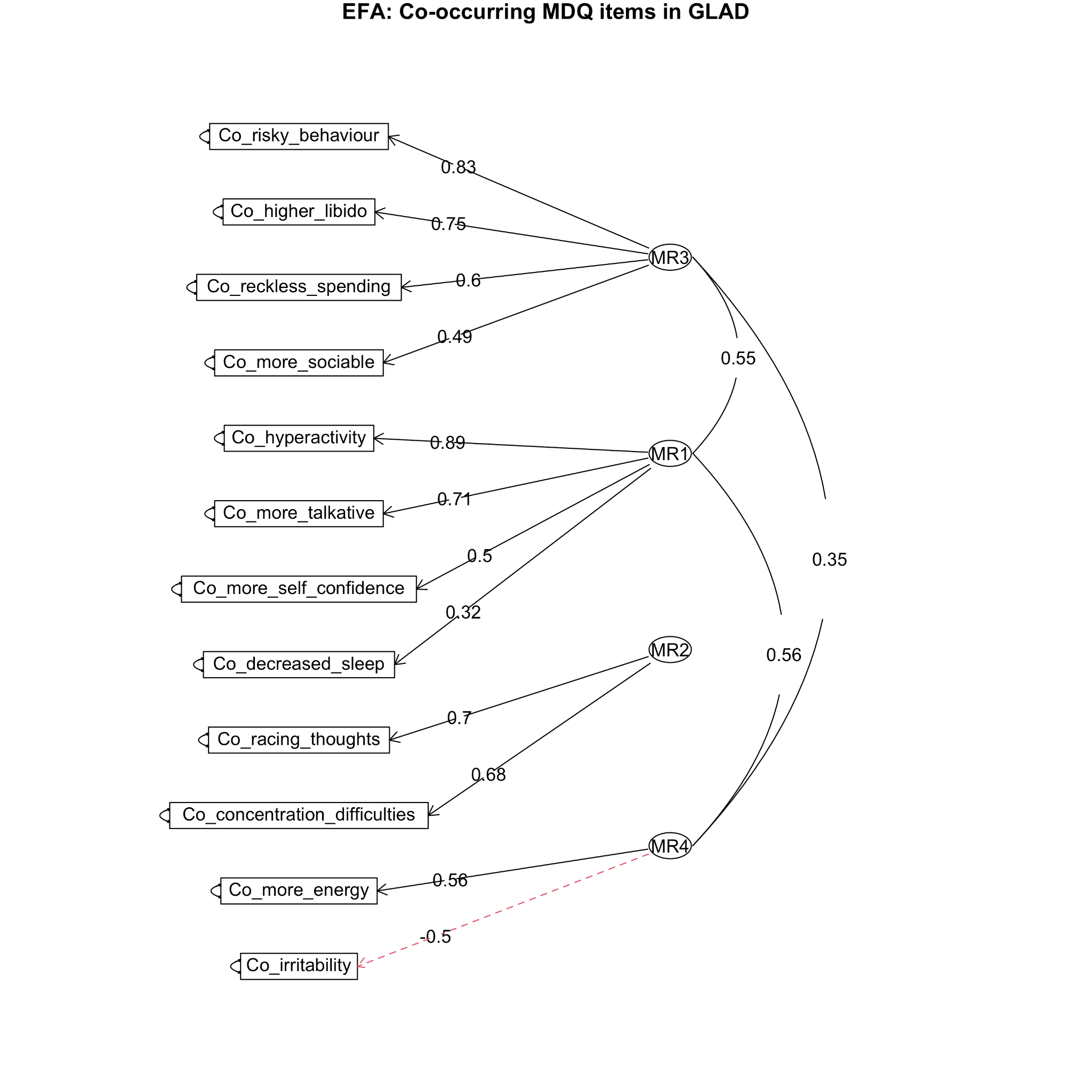
**

**Figure S8. Exploratory factor analysis (EFA): one factor solution of 12 lifetime Mood Disorder Questionnaire (MDQ) items in affected participants.**

*EFA was performed with the psych R package. Oblimin rotation method was used to allow the latent factors to correlate with each other and the factoring method was “minimum residuals”.*

**
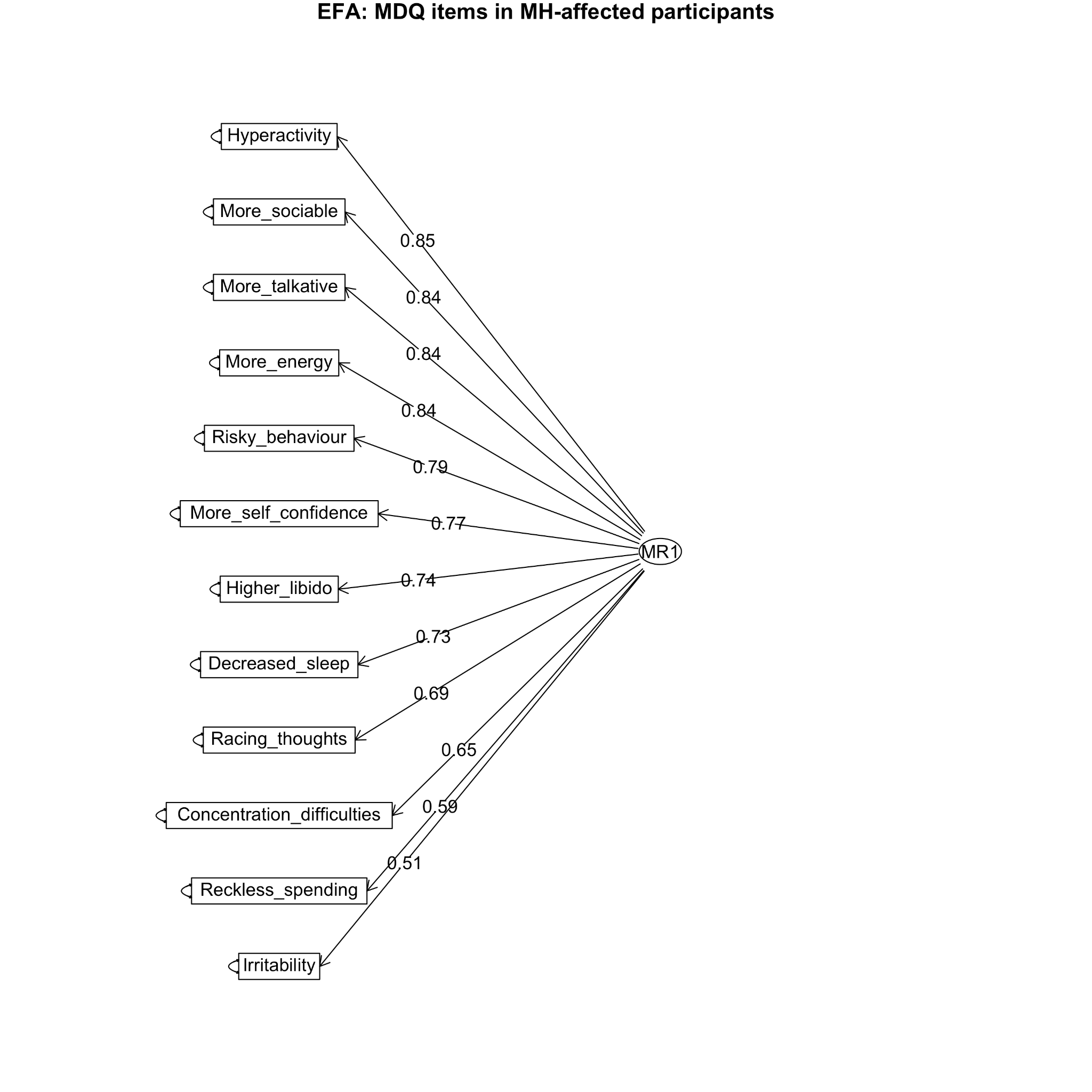
**

**Figure S9. Exploratory factor analysis (EFA): two factor solution of 12 lifetime Mood Disorder Questionnaire (MDQ) items in affected participants.**

*EFA was performed with the psych R package. Oblimin rotation method was used to allow the latent factors to correlate with each other and the factoring method was “minimum residuals”.*

**
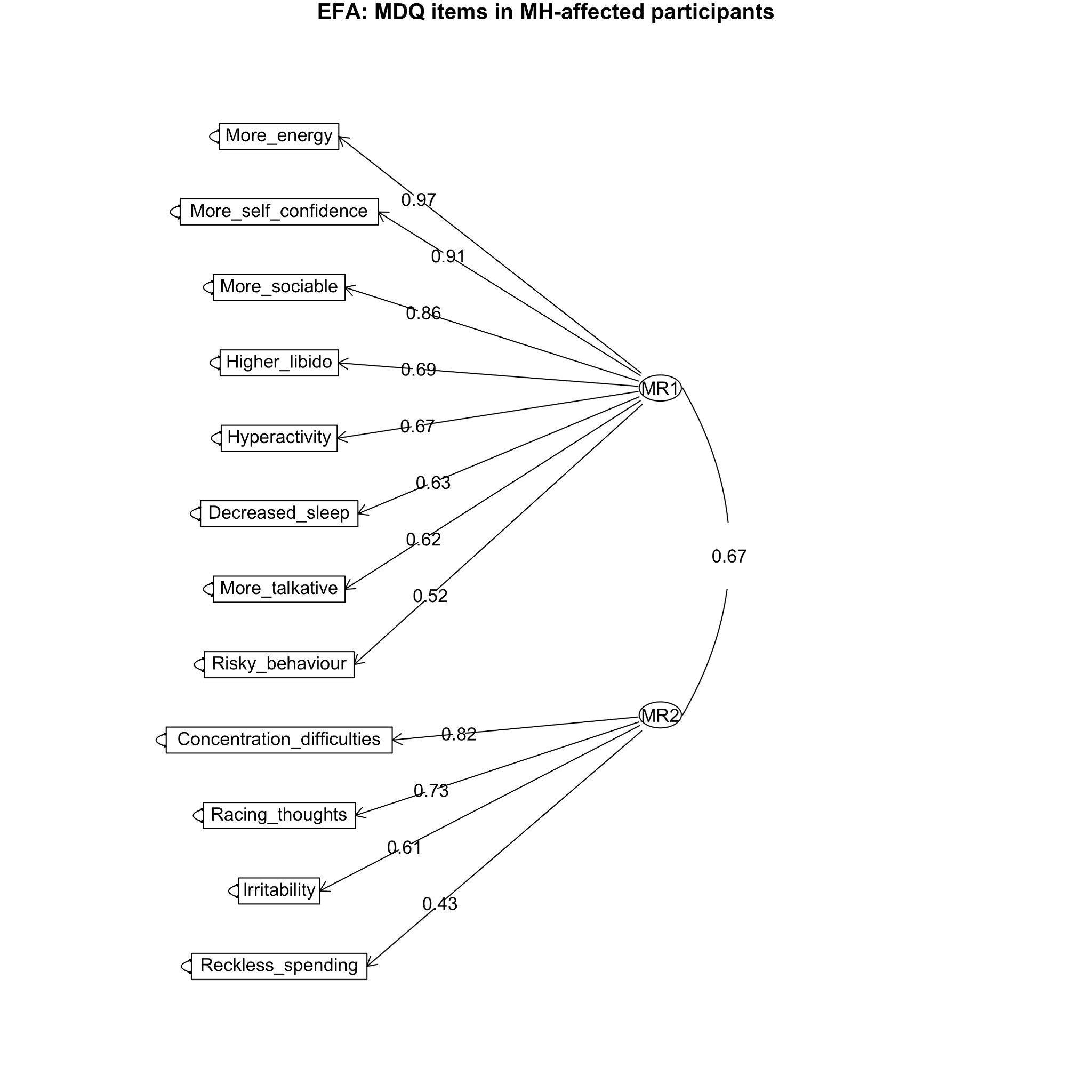
**

**Figure S10. Exploratory factor analysis (EFA): three factor solution of 12 lifetime Mood Disorder Questionnaire (MDQ) items in affected participants.**

*EFA was performed with the psych R package. Oblimin rotation method was used to allow the latent factors to correlate with each other and the factoring method was “minimum residuals”.*

**
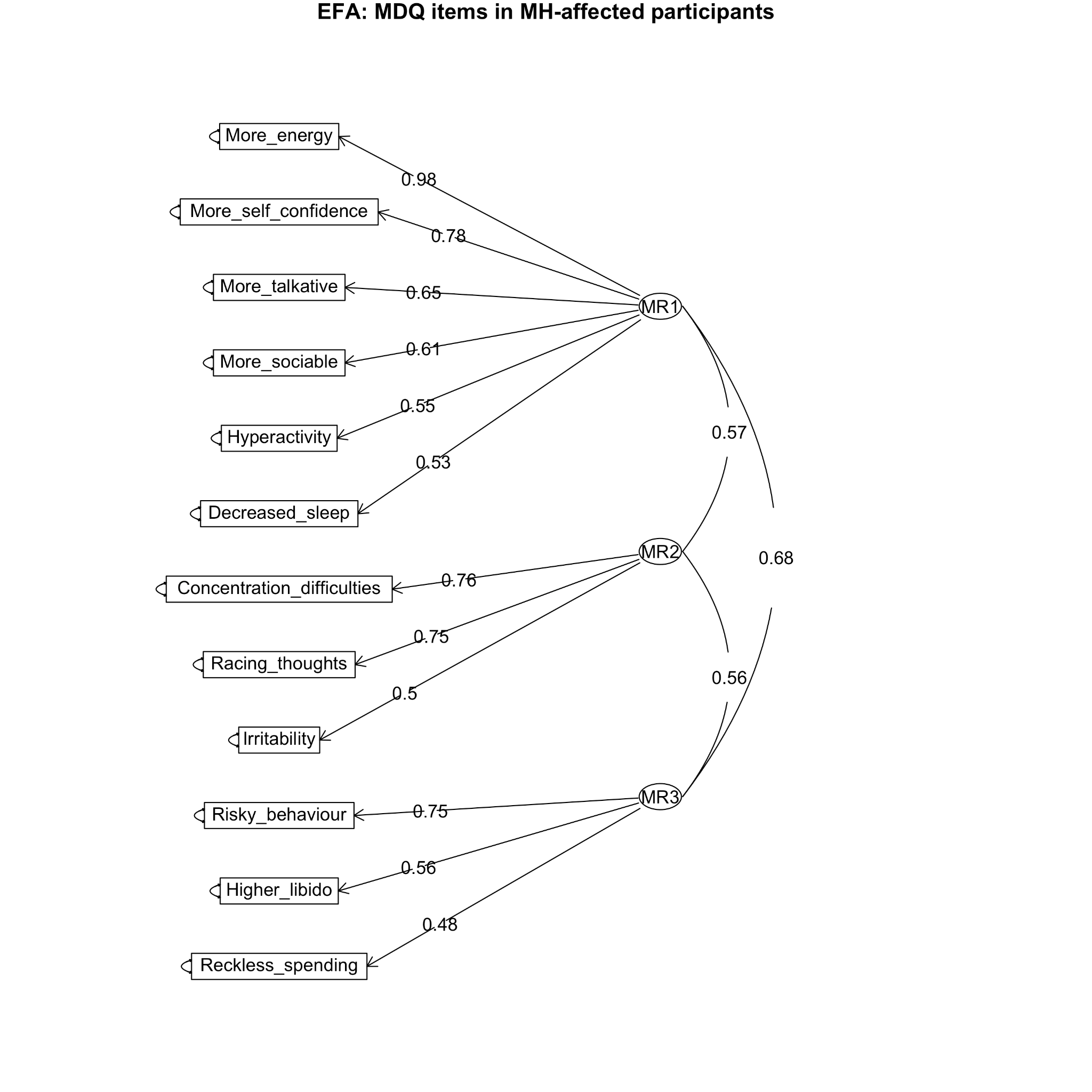
**

**Figure S11. Exploratory factor analysis (EFA): four factor solution of 12 lifetime Mood Disorder Questionnaire (MDQ) items in affected participants.**

*EFA was performed with the psych R package. Oblimin rotation method was used to allow the latent factors to correlate with each other and the factoring method was “minimum residuals”.*

**
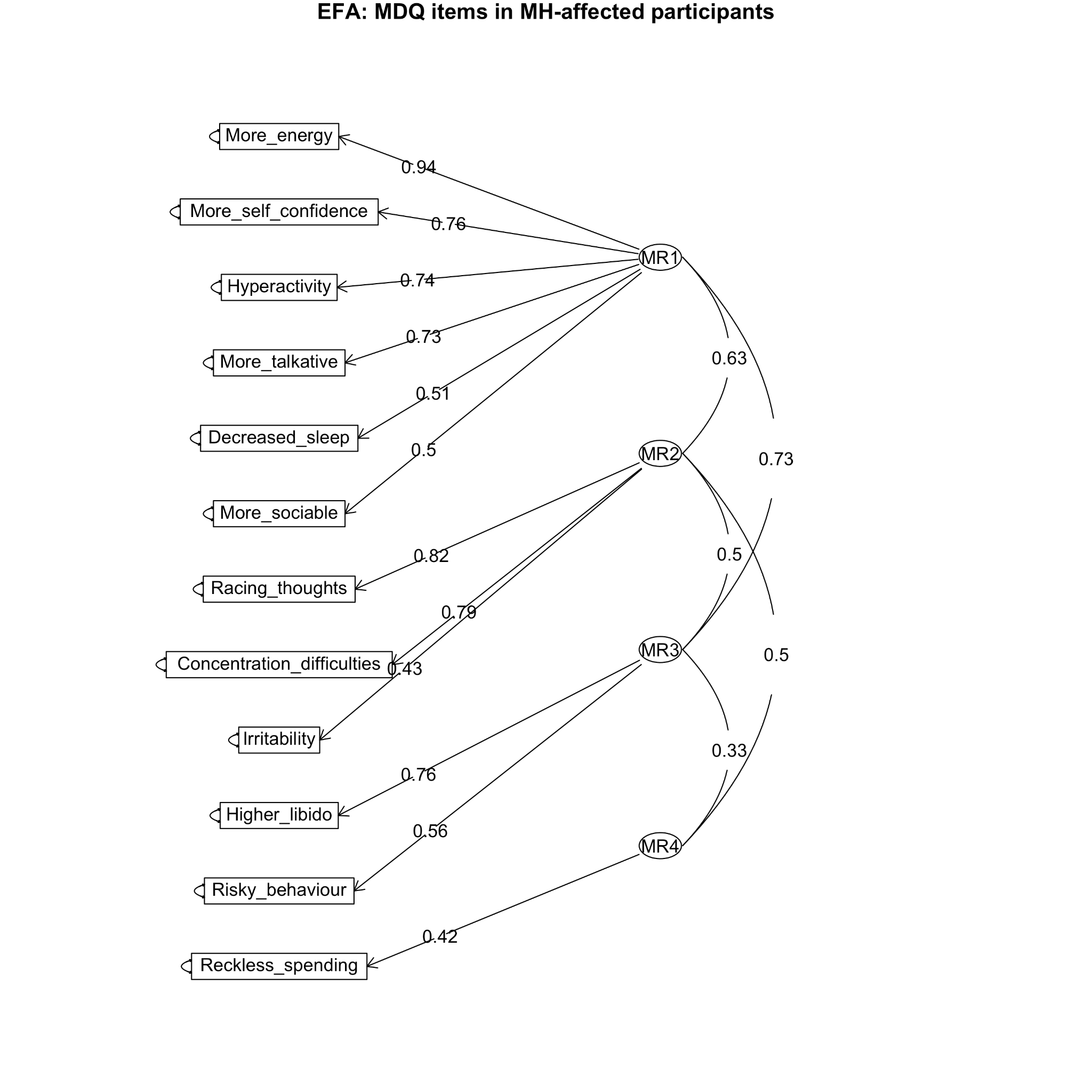
**

**Figure S12. Exploratory factor analysis (EFA): one factor solution of 12 lifetime Mood Disorder Questionnaire (MDQ) items in unaffected participants.**

*EFA was performed with the psych R package. Oblimin rotation method was used to allow the latent factors to correlate with each other and the factoring method was “minimum residuals”.*

**
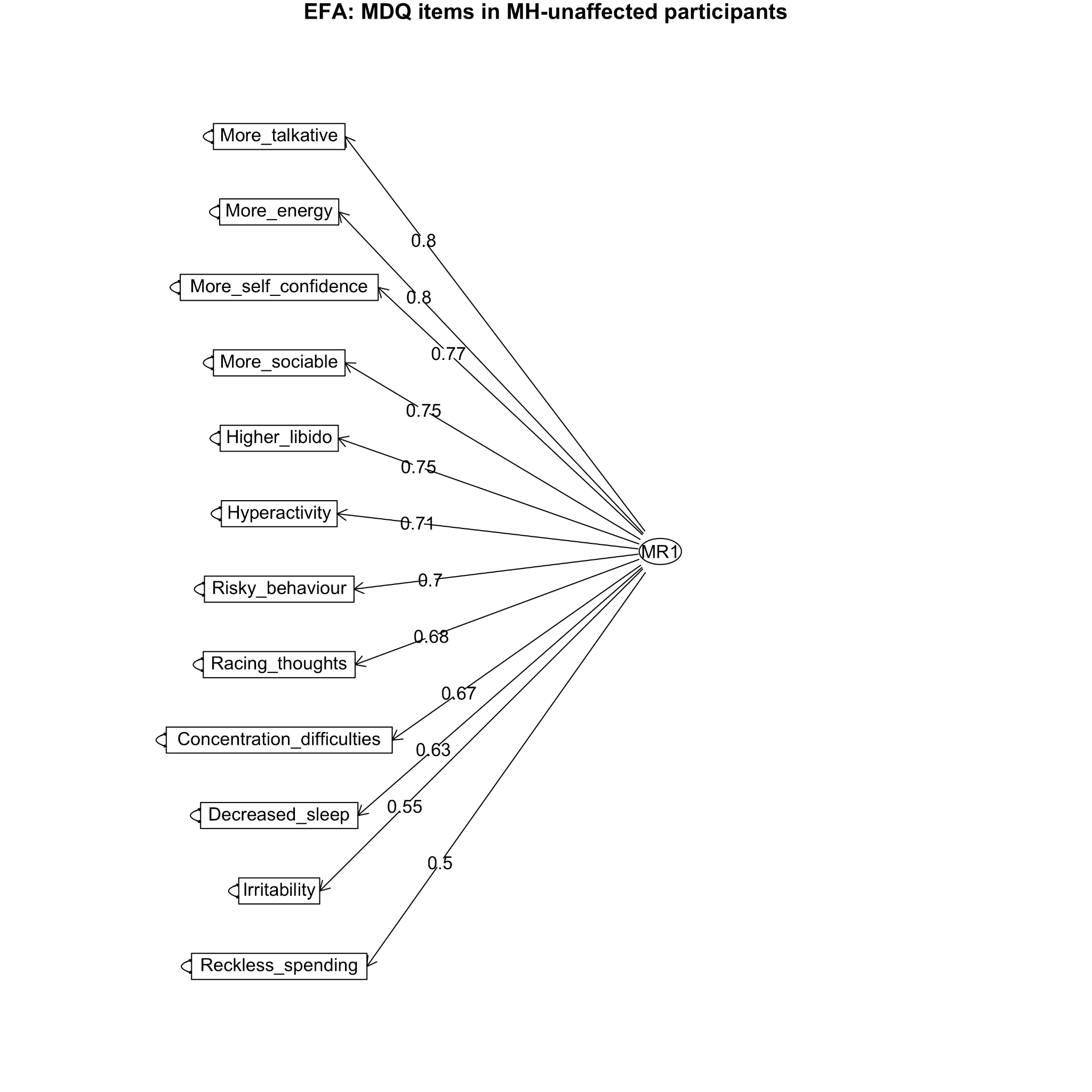
**

**Figure S13. Exploratory factor analysis (EFA): two factor solution of 12 lifetime Mood Disorder Questionnaire (MDQ) items in unaffected participants.**

*EFA was performed with the psych R package. Oblimin rotation method was used to allow the latent factors to correlate with each other and the factoring method was “minimum residuals”.*

**
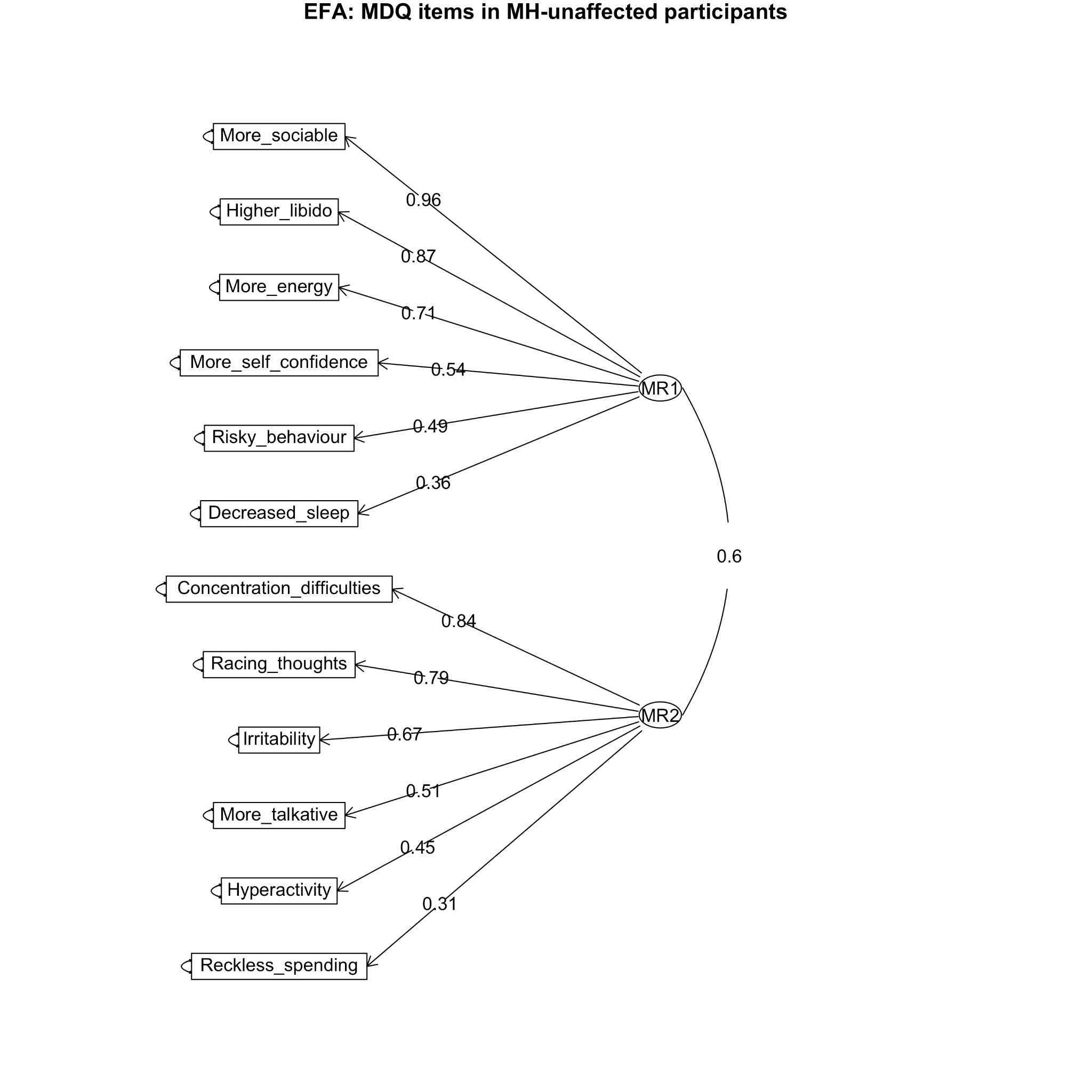
**

**Figure S14. Exploratory factor analysis (EFA): three factor solution of 12 lifetime Mood Disorder Questionnaire (MDQ) items in unaffected participants.**

*EFA was performed with the psych R package. Oblimin rotation method was used to allow the latent factors to correlate with each other and the factoring method was “minimum residuals”.*

**
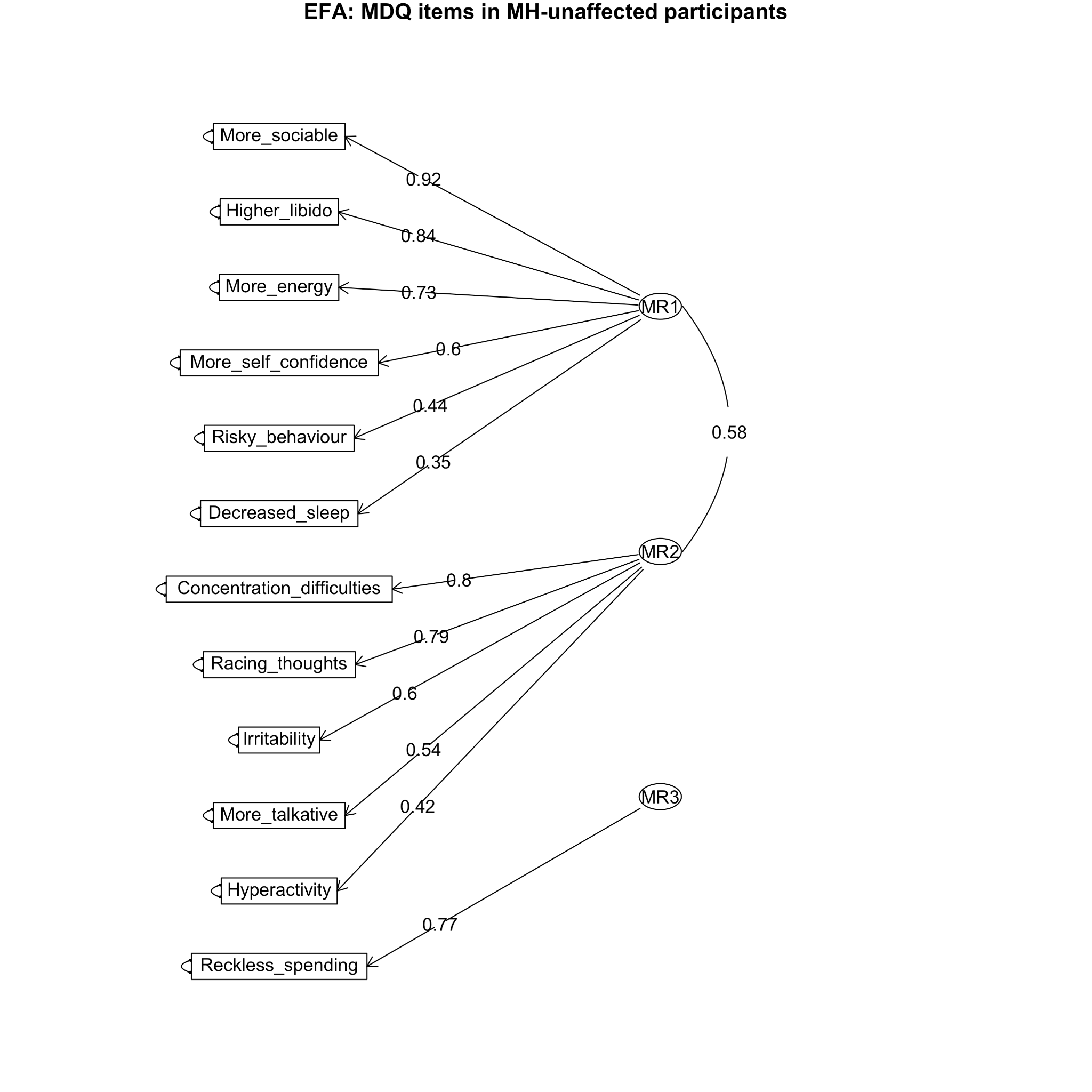
**

**Figure S15. Exploratory factor analysis (EFA): four factor solution of 12 lifetime Mood Disorder Questionnaire (MDQ) items in unaffected participants.**

*EFA was performed with the psych R package. Oblimin rotation method was used to allow the latent factors to correlate with each other and the factoring method was “minimum residuals”.*

**
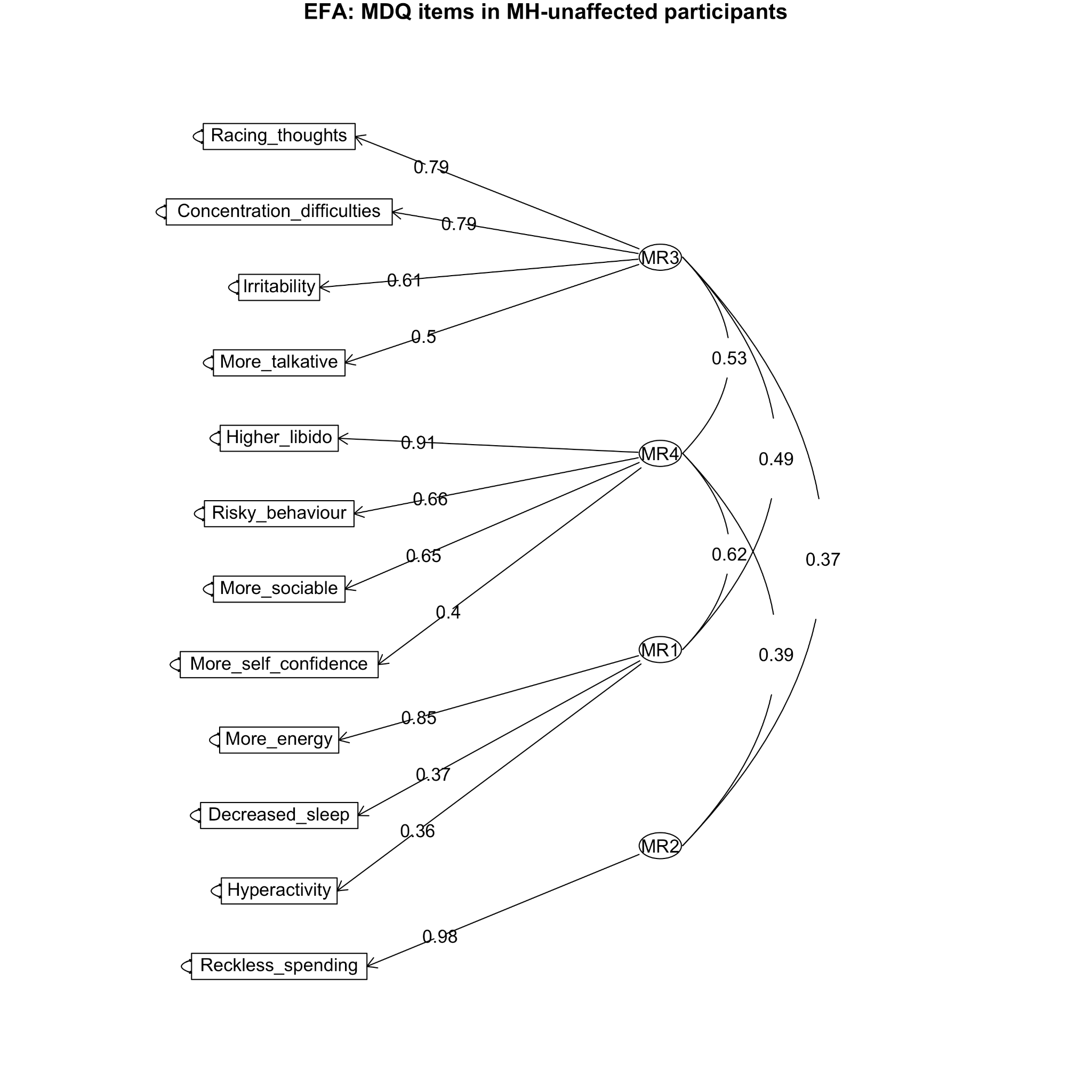
**

**Figure S16. Raw factor scores from factor analysis of 12 concurrent manic symptoms measured by the Mood Disorder Questionnaire (MDQ) in affected participants.**

*The item “more active” was removed due to a correlation of 0.87 with “more energy”. Factor scores were computed, based on the best-fitting model identified in EFA, with the lavaan R package following confirmatory factor analysis (CFA).*

**
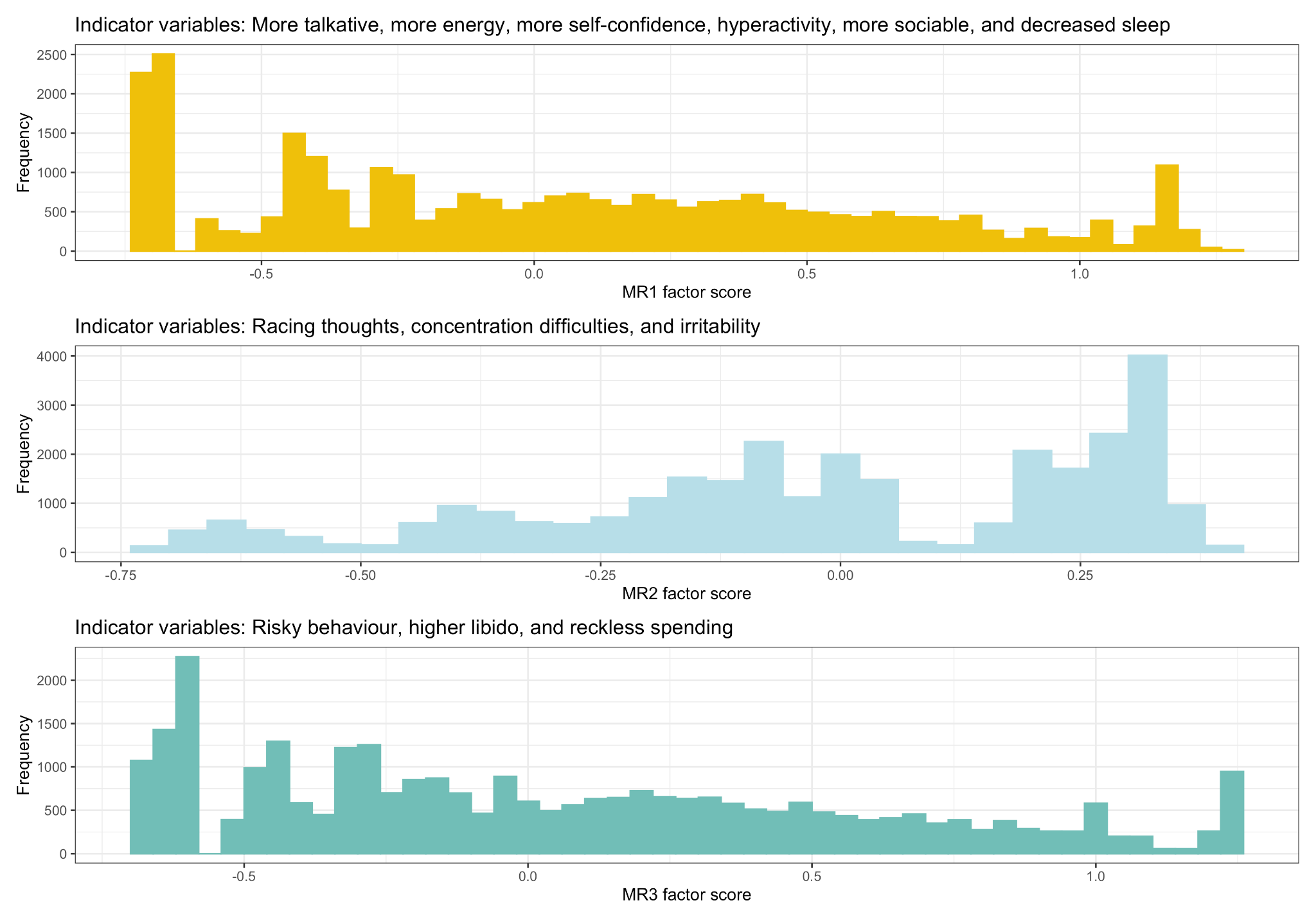
**

**Figure S17. Raw factor scores from factor analysis of 12 lifetime manic symptoms measured by the Mood Disorder Questionnaire (MDQ) in affected participants.**

*The item “more active” was removed due to a correlation of 0.9 with “more energy”. Factor scores were computed, based on the best-fitting model identified in EFA, with the lavaan R package following confirmatory factor analysis (CFA).*

**
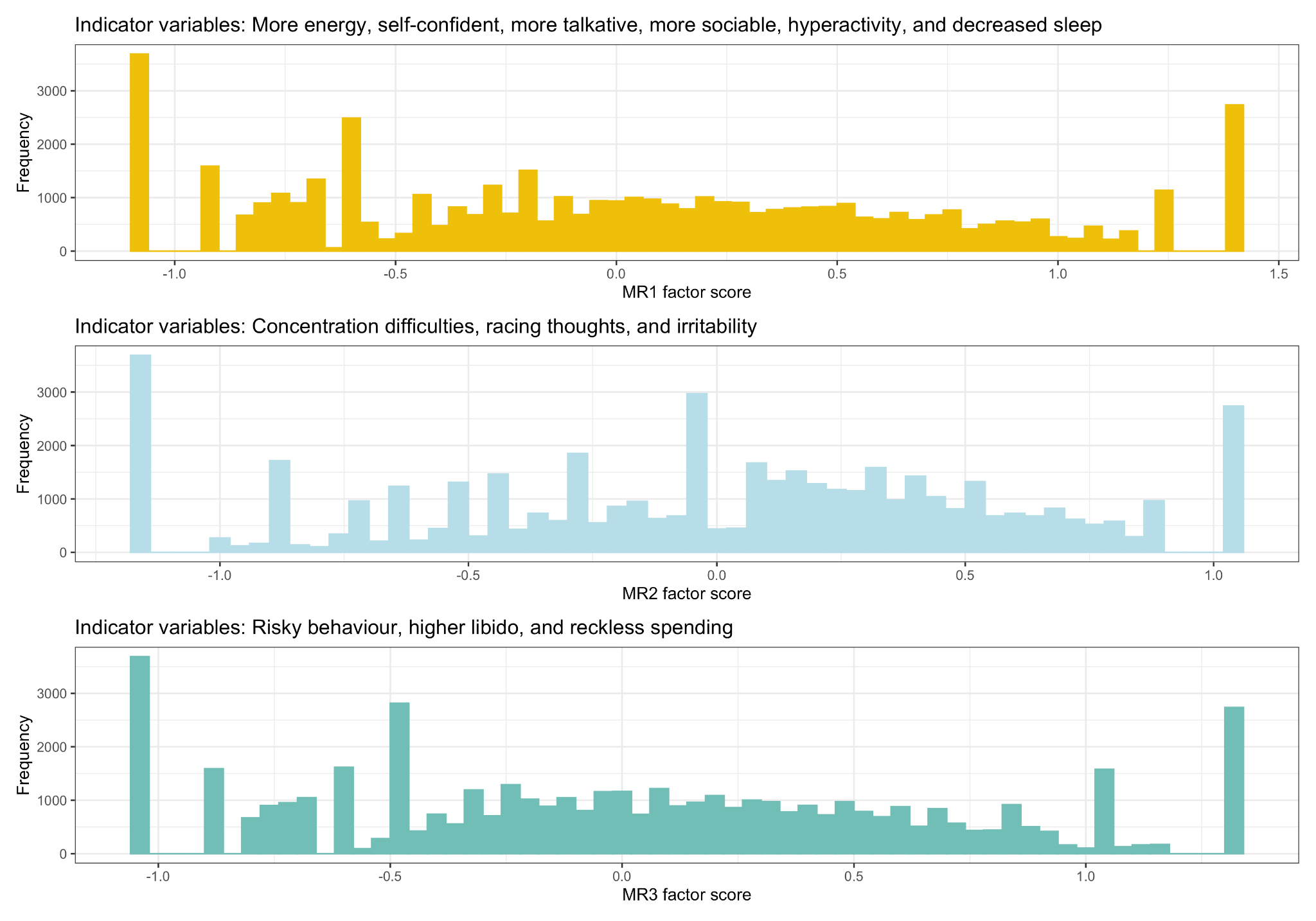
**

**Figure S18. Principal component analysis (PCA) plots.**

*Top: Principal component analysis (PCA) plot of Genetic Links to Anxiety and Depression (GLAD) Study participants Bottom: PCA plot of GLAD Study participants and COVID-19 Psychiatry and Neurological Genetics (COPING) Study participants*

**
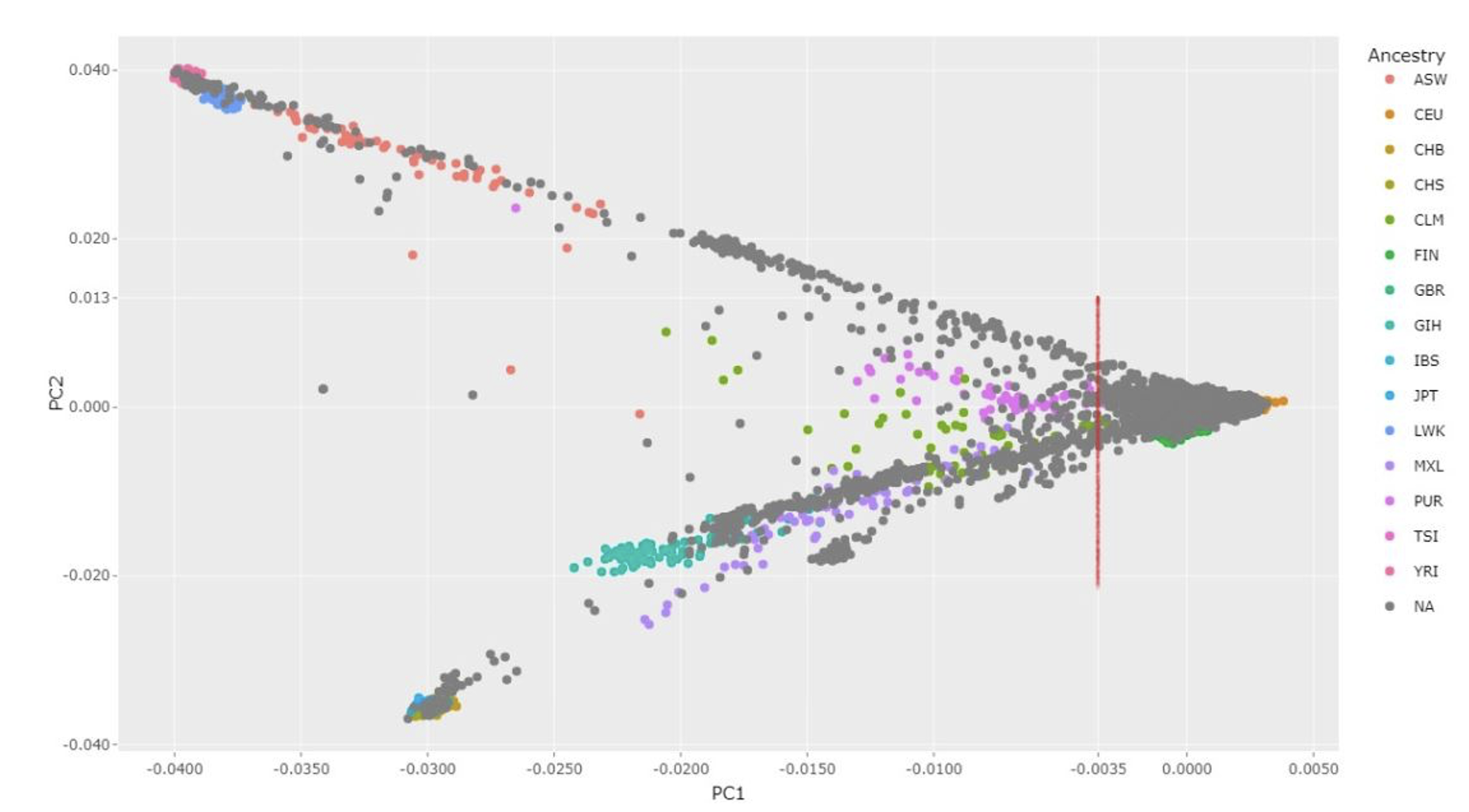
**

**
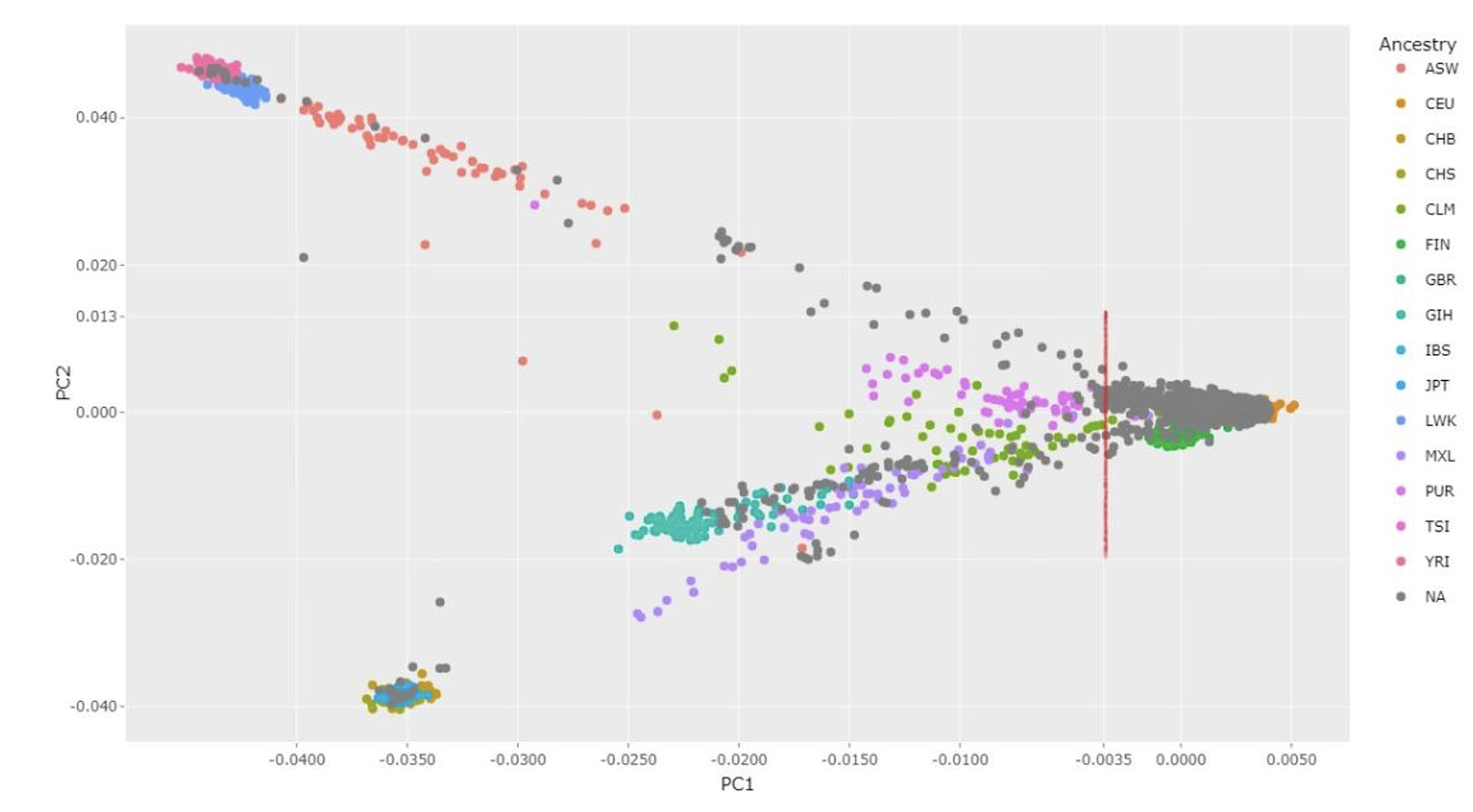
**

*Note: ASW (African Ancestry in SW USA), CEU (Europeans, from Utah), CHB (Northern Han Chinese from Beijing), CHS (Southern Han Chinese, from Shanghai), CLM (Colombian in Medellín, Colombia), FIN (Finnish in Finland), GBR (Western Europeans from Britain), IBS (Southern Europeans from Spain), JPT (Japanese in Tokyo, Japan), LWK (Luhya from Webuye, Kenya), MXL (Mexican ancestry in Los Angeles, CA, USA), PUR (Puerto Rican in Puerto Rico), TSI (Southern Europeans from Tuscany in Italy), YRI (Yoruba in Ibadan, Nigeria), GIH (Gujarati Indians in Houston, Texas, USA), NA (GLADv2 or COPING NBRv1)*

**Figure S19. Quantile-quantile (QQ) plot and Manhattan plot of genome-wide association study (GWAS) results of the concurrent manic symptom sum score measured by the Mood Disorder Questionnaire (MDQ) in affected participants of European ancestry (N=11,568).**

*GWAS was performed with REGENIE covarying for the first ten ancestry principal components and genotyping batch. Manhattan and QQ plots were produced using FUMA.*

**
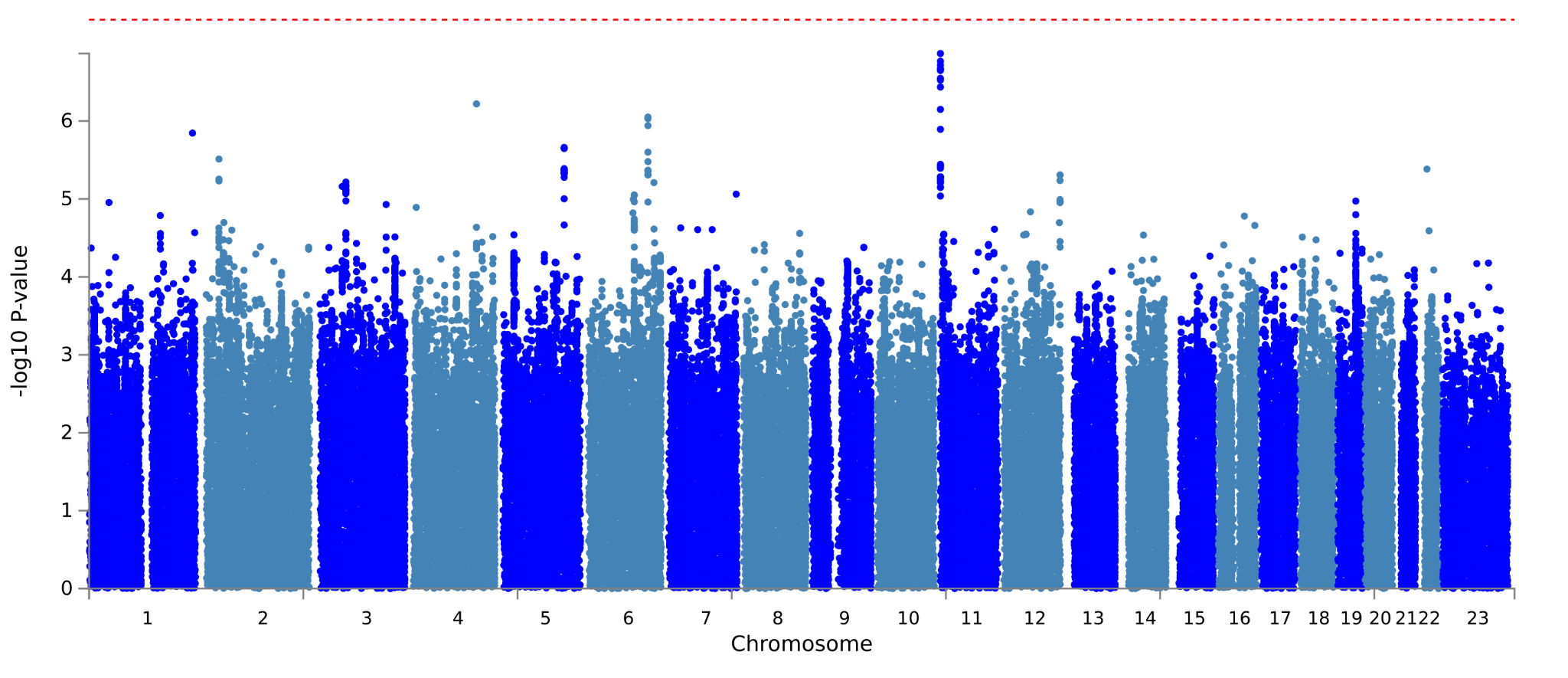
**

**
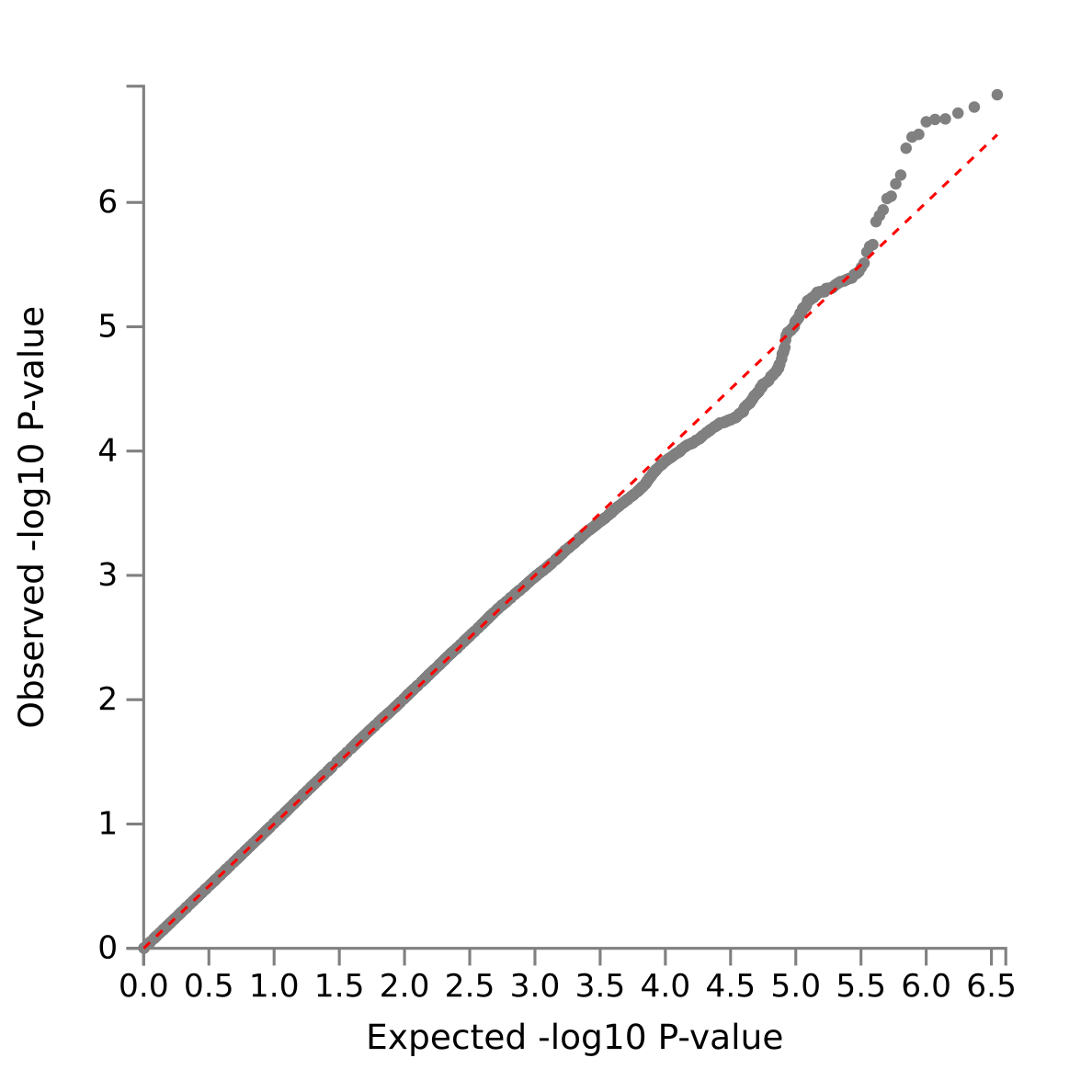
**

**Figure S20. Quantile-quantile (QQ) plot and Manhattan plot of genome-wide association study (GWAS) results of concurrent energy/activity factor measured by the Mood Disorder Questionnaire (MDQ) in affected participants of European ancestry (N=11,568).** *GWAS was performed with REGENIE covarying for the first ten ancestry principal components and genotyping batch. Manhattan and QQ plots were produced using FUMA.*

**
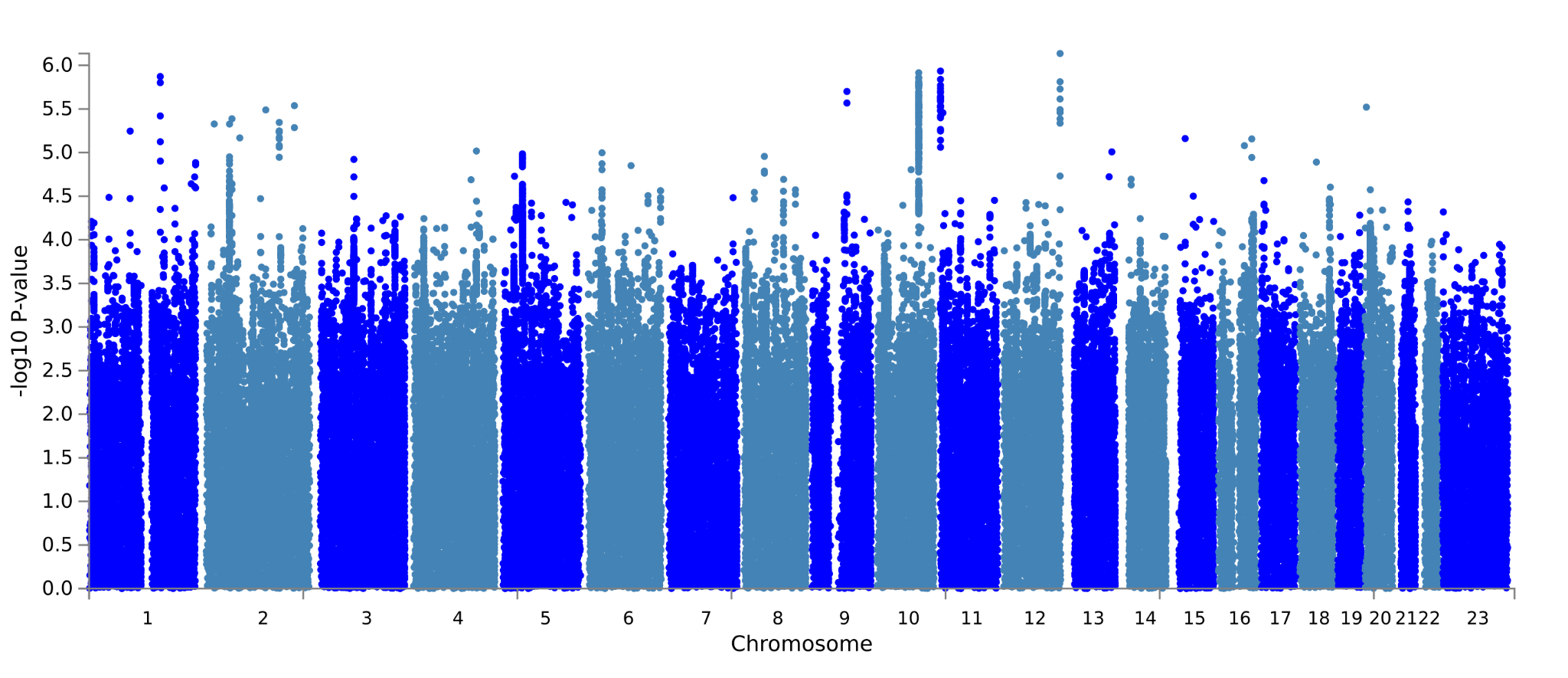
**

**
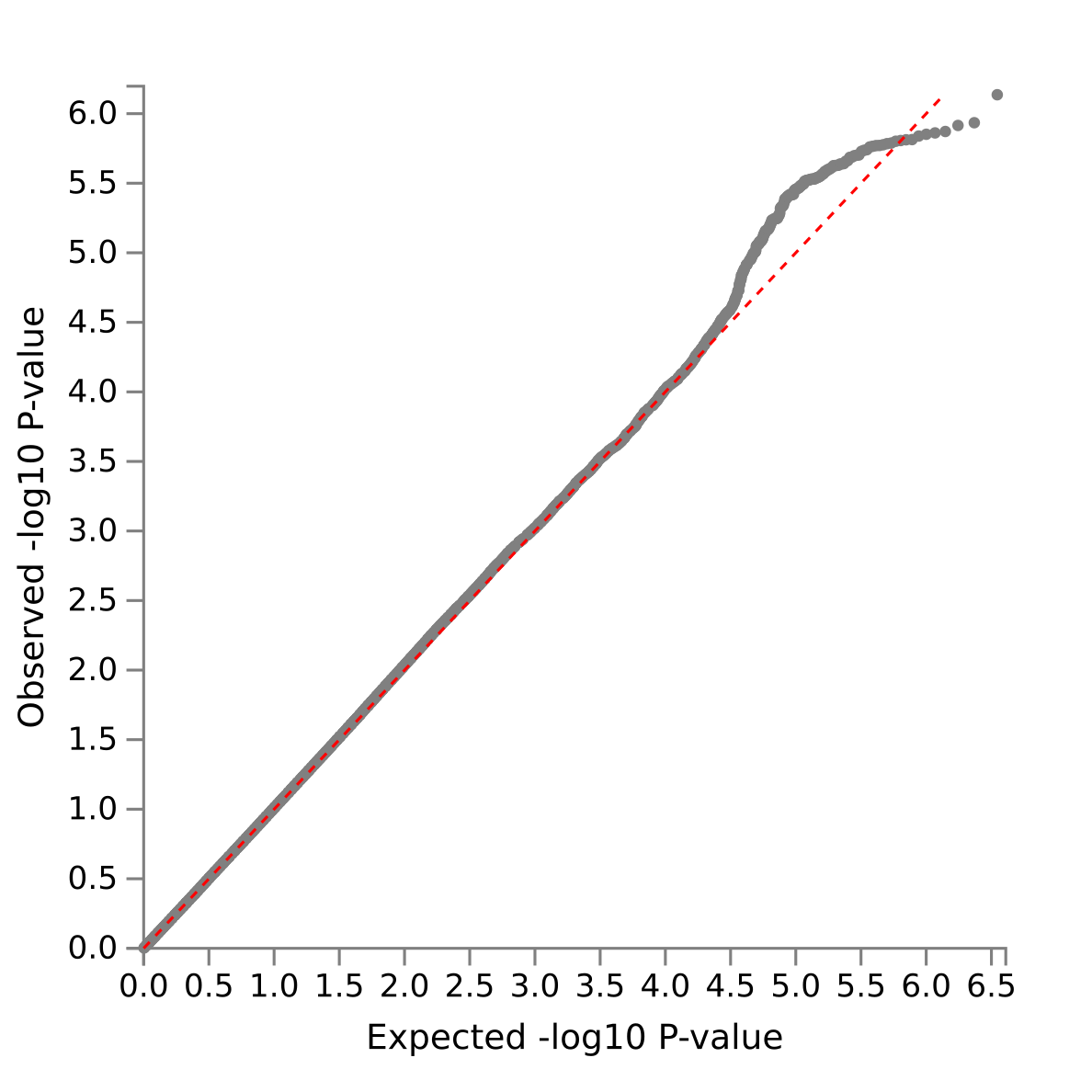
**

**Figure S21. Quantile-quantile (QQ) plot and Manhattan plot of the genome-wide association study (GWAS) results of concurrent cognitive factor measured by the Mood Disorder Questionnaire (MDQ) in affected participants of European ancestry (N=11,568).** *GWAS was performed with REGENIE covarying for the first ten ancestry principal components and genotyping batch. Manhattan and QQ plots were produced using FUMA.*

**
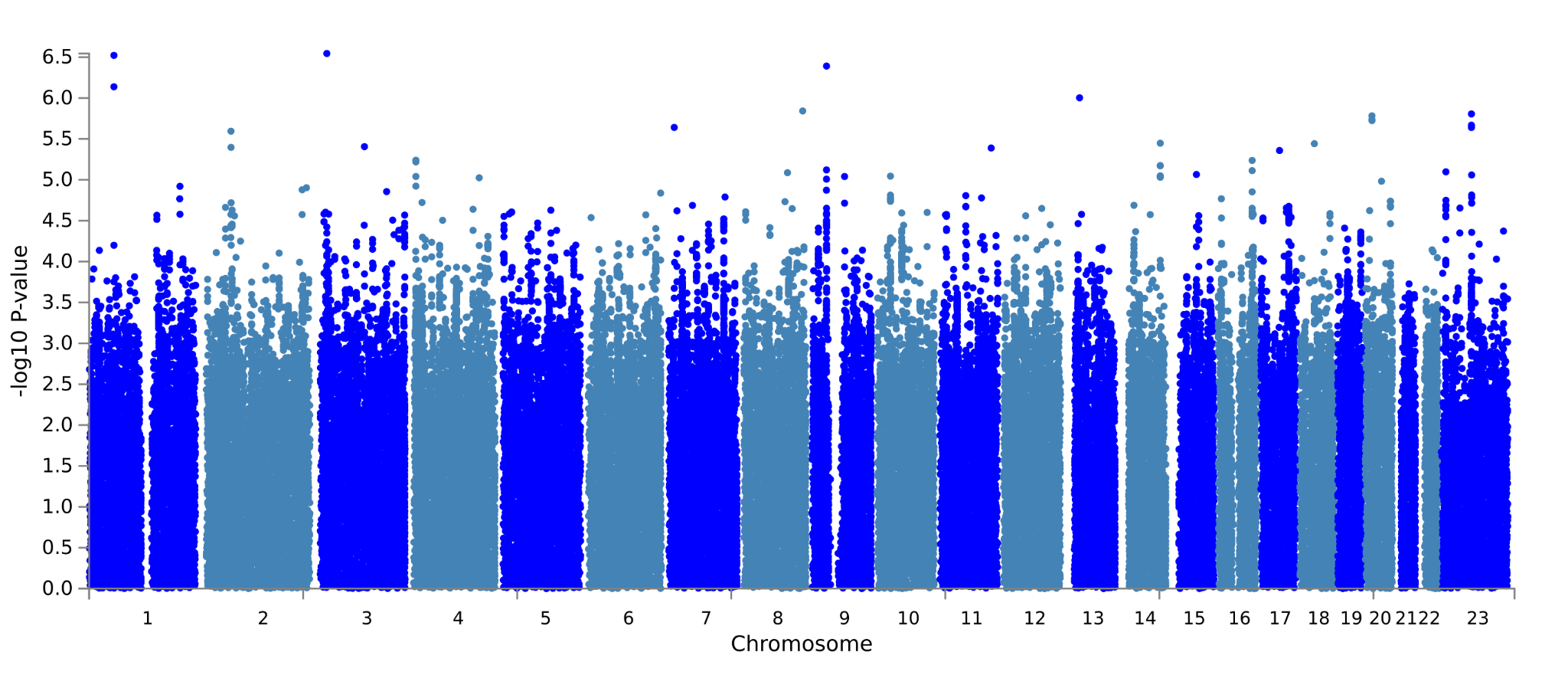
**

**
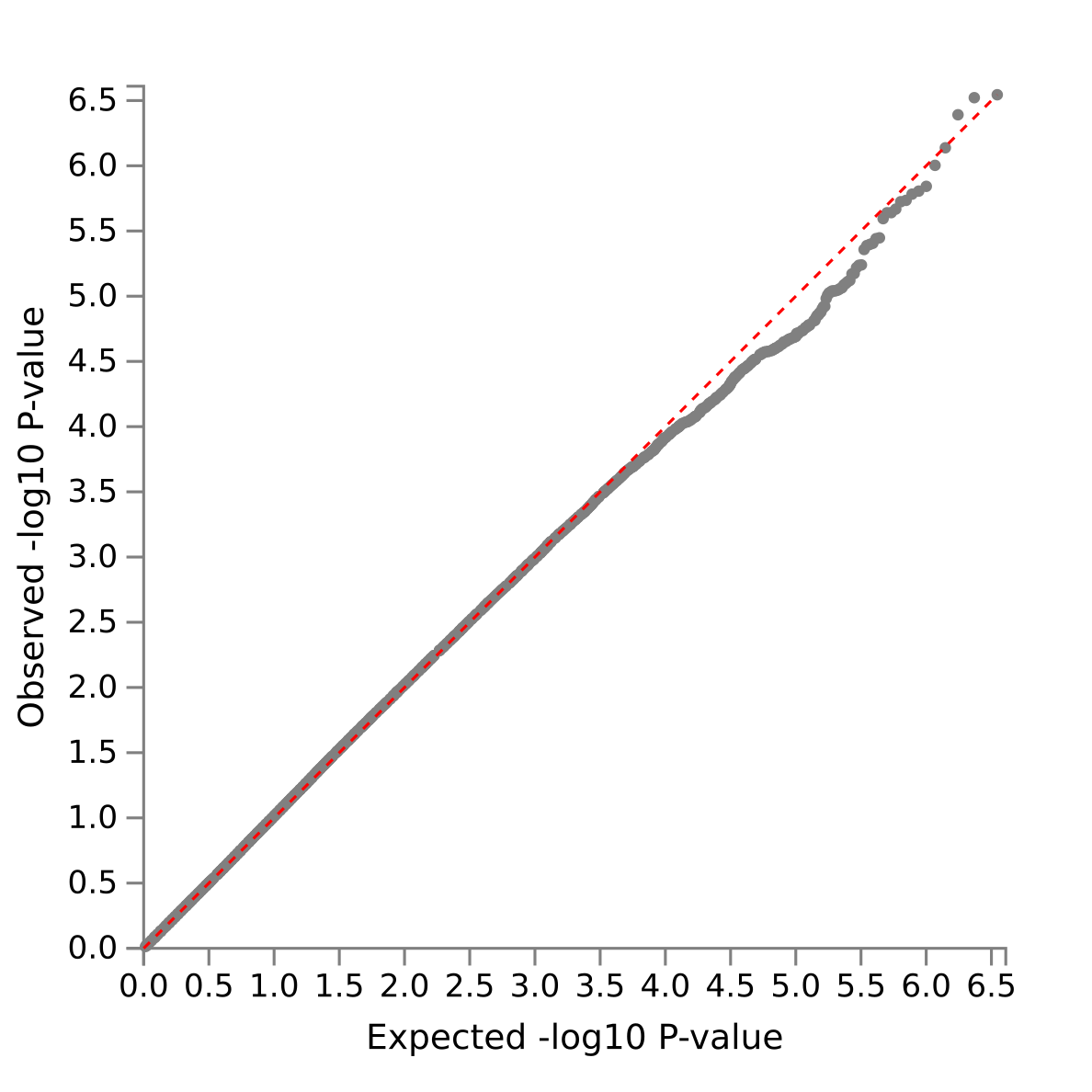
**

**Figure S22. Quantile-quantile (QQ) plot and Manhattan plot of genome-wide association study (GWAS) results of the concurrent impulsivity factor measured by the Mood Disorder Questionnaire (MDQ) in affected participants of European ancestry (N=11,568).** *GWAS was performed with REGENIE covarying for the first ten ancestry principal components and genotyping batch. Manhattan and QQ plots were produced using FUMA.*

**
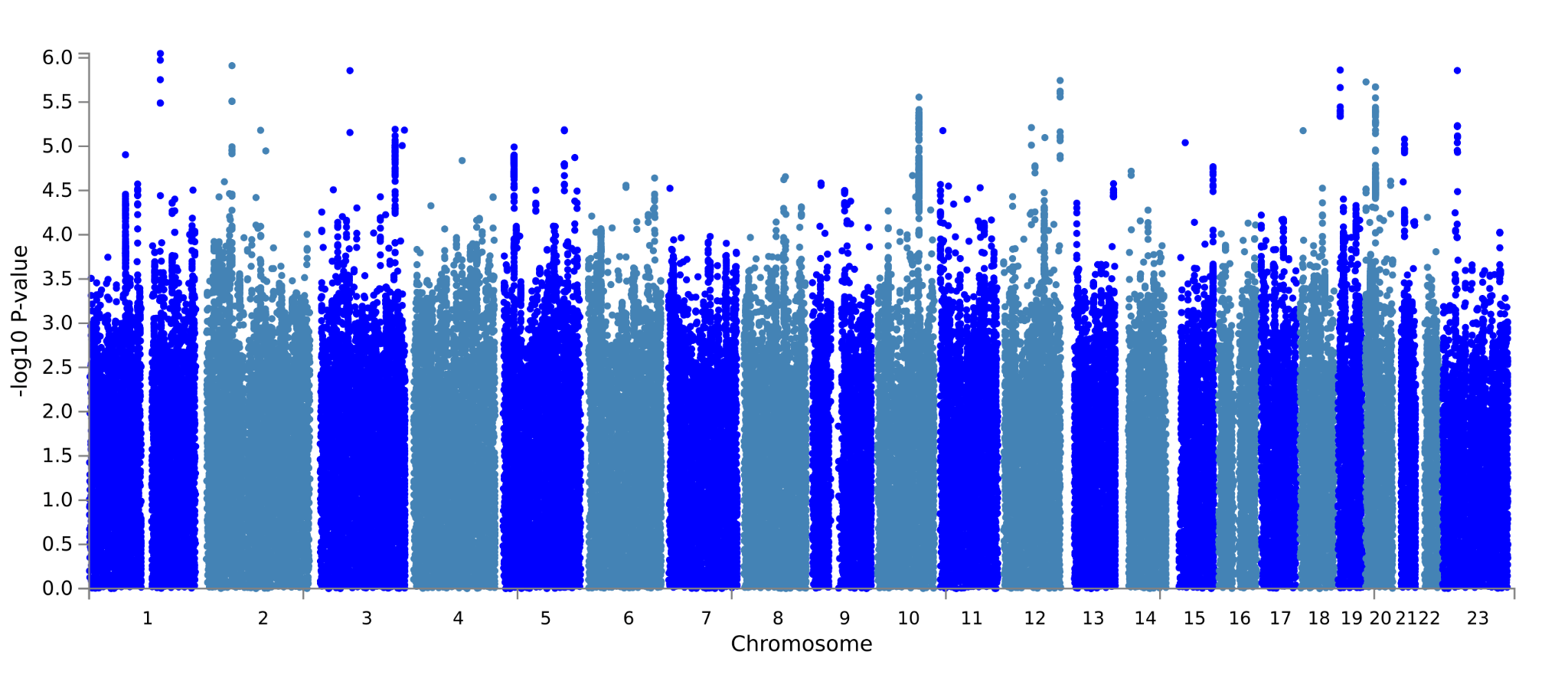
**

**
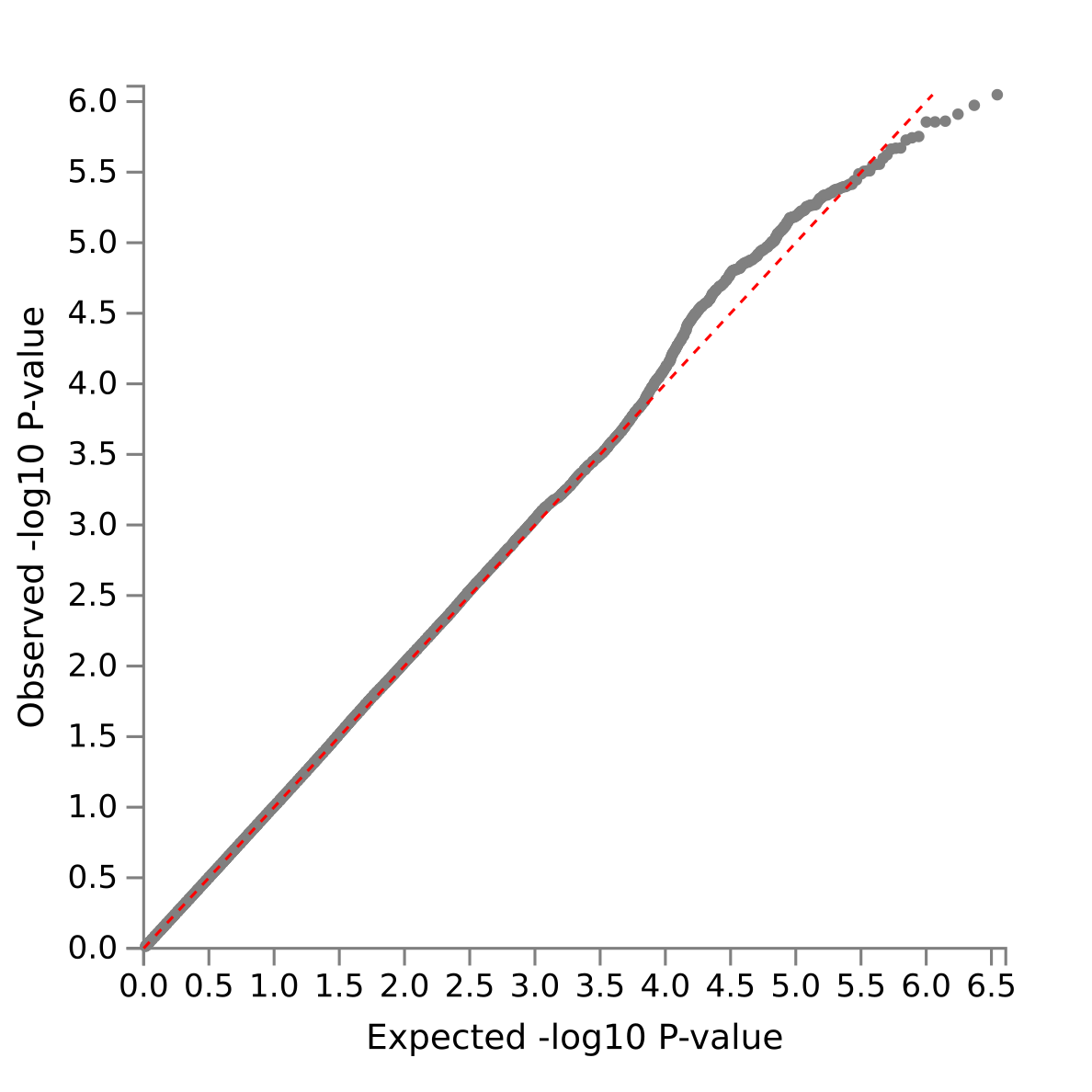
**

**Figure S23. Quantile-quantile (QQ) plot and Manhattan plot of genome-wide association study (GWAS) results of the lifetime manic symptom sum score measured by the Mood Disorder Questionnaire (MDQ) in affected participants of European ancestry (N=19,859).** *GWAS was performed with REGENIE covarying for the first ten ancestry principal components and genotyping batch. Manhattan and QQ plots were produced using FUMA.*


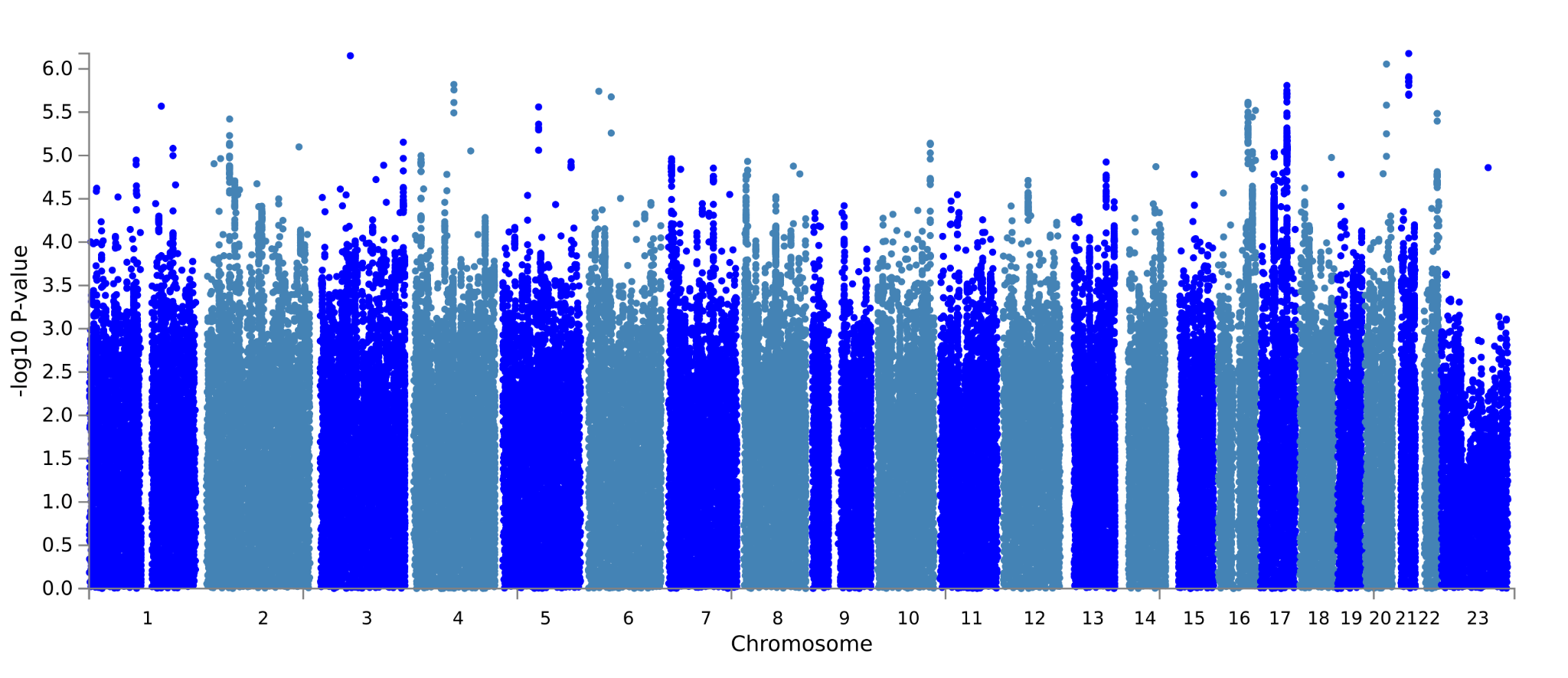


**
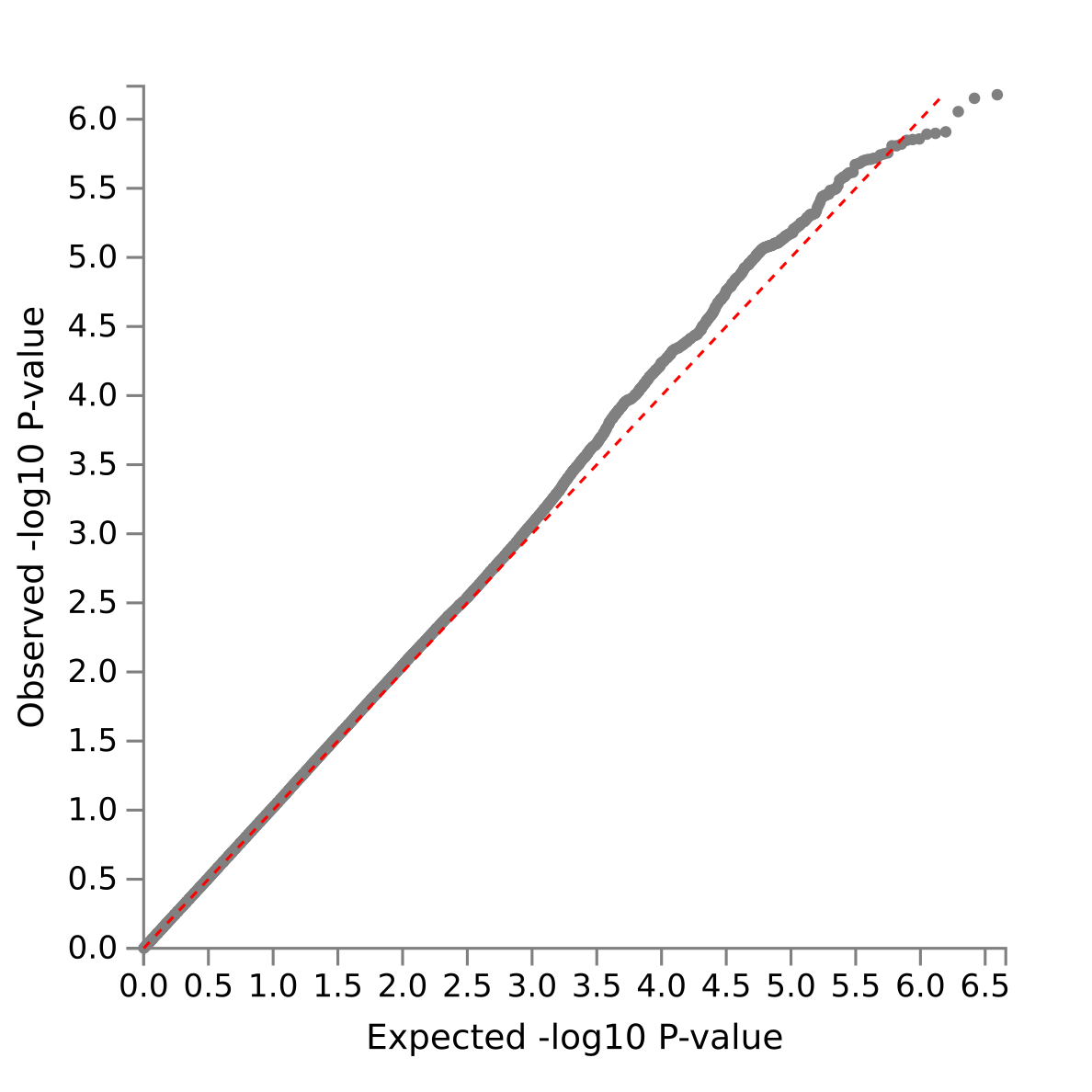
**

**Figure S24. Quantile-quantile (QQ) plot and Manhattan plot of genome-wide association study (GWAS) results of the lifetime energy/activity factor measured by the Mood Disorder Questionnaire (MDQ) in affected participants of European ancestry (N=19,859).** *GWAS was performed with REGENIE covarying for the first ten ancestry principal components and genotyping batch. Manhattan and QQ plots were produced using FUMA.*

**
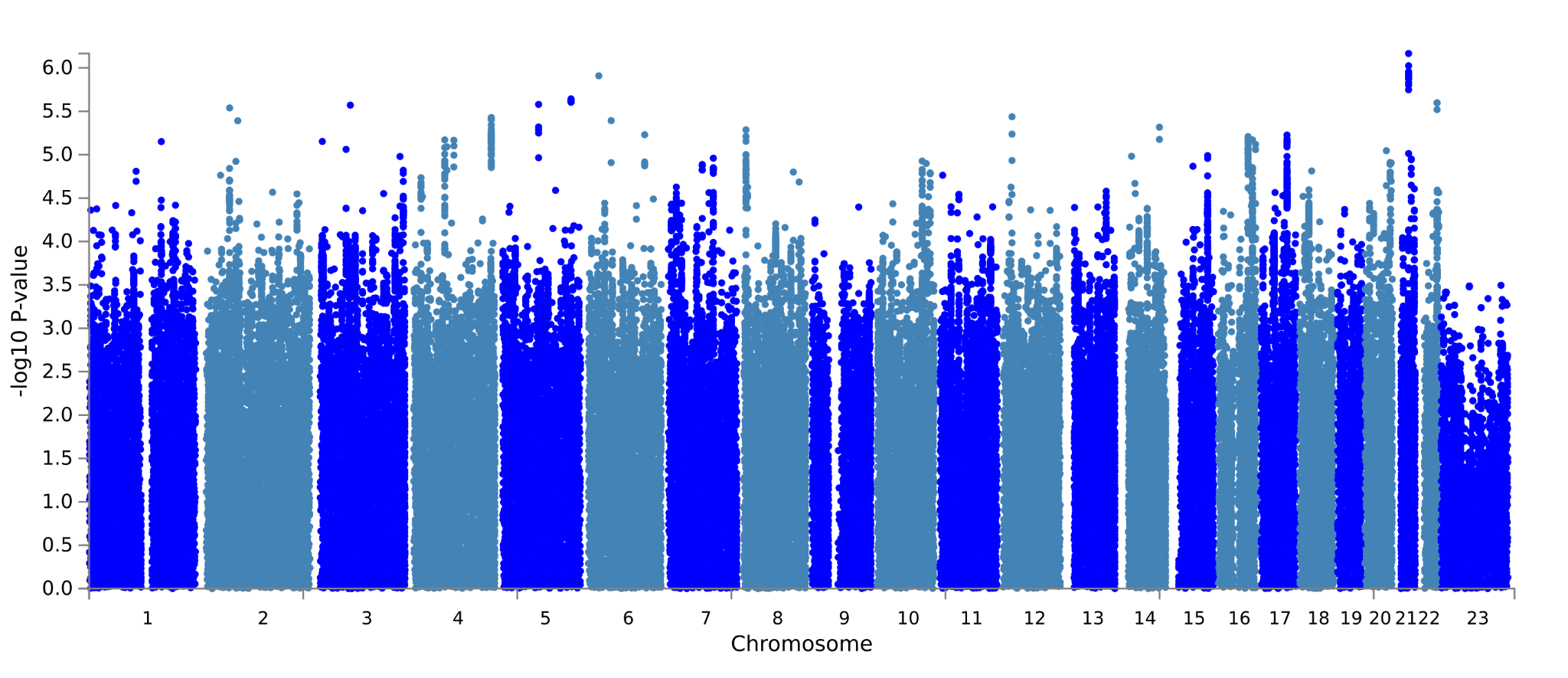
**

**
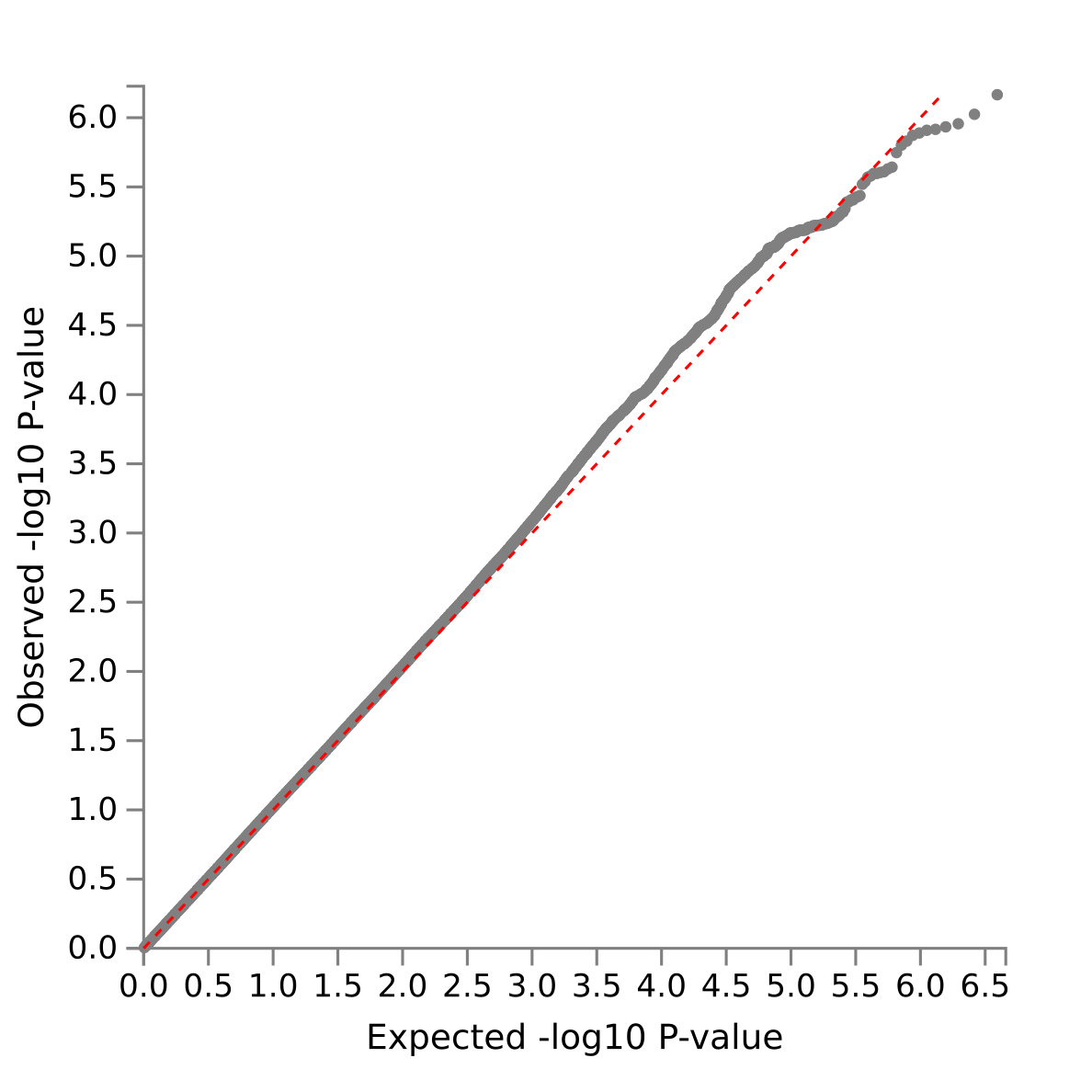
**

**Figure S25. Quantile-quantile (QQ) plot and Manhattan plot of genome-wide association study (GWAS) results of the lifetime cognitive factor measured by the Mood Disorder Questionnaire (MDQ) in affected participants of European ancestry (N=19,859).**

*GWAS was performed with REGENIE covarying for the first ten ancestry principal components and genotyping batch. Manhattan and QQ plots were produced using FUMA.*

**
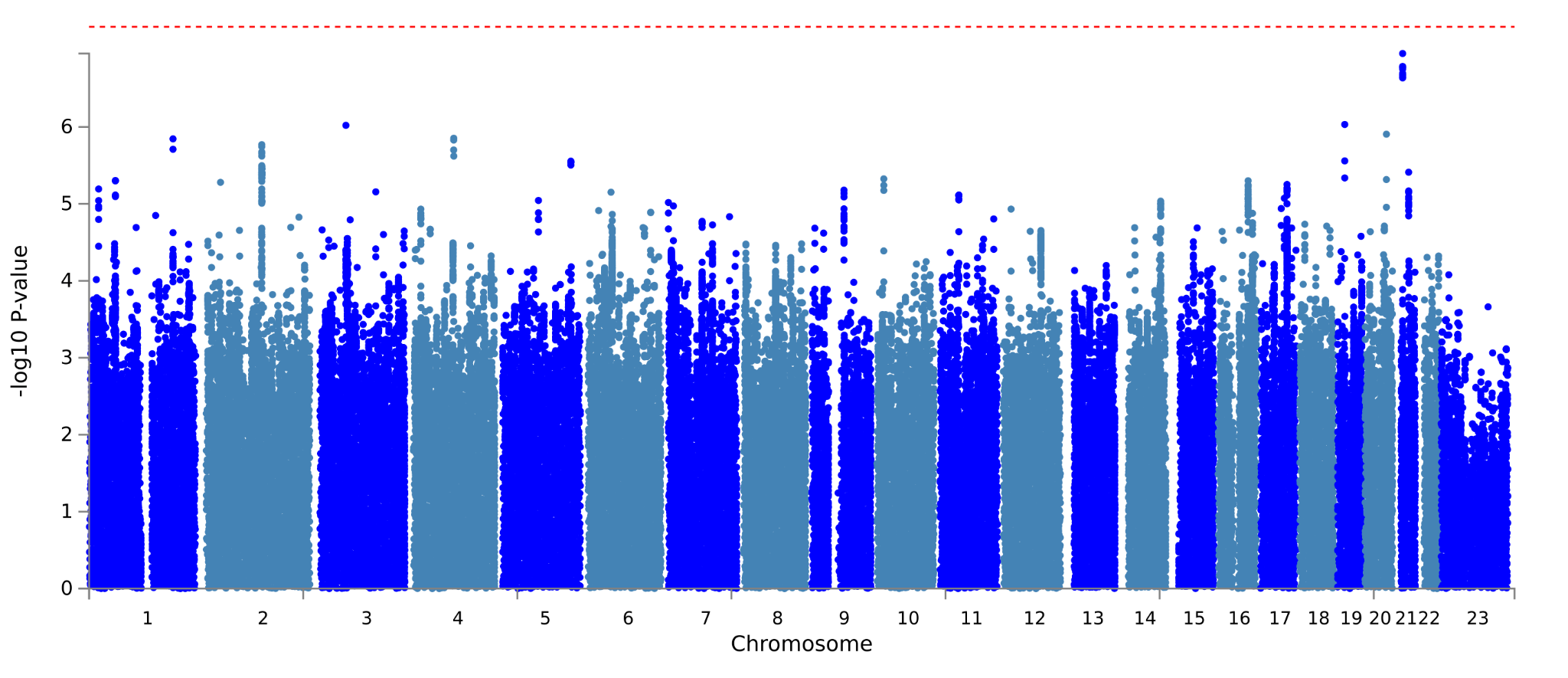
**

**
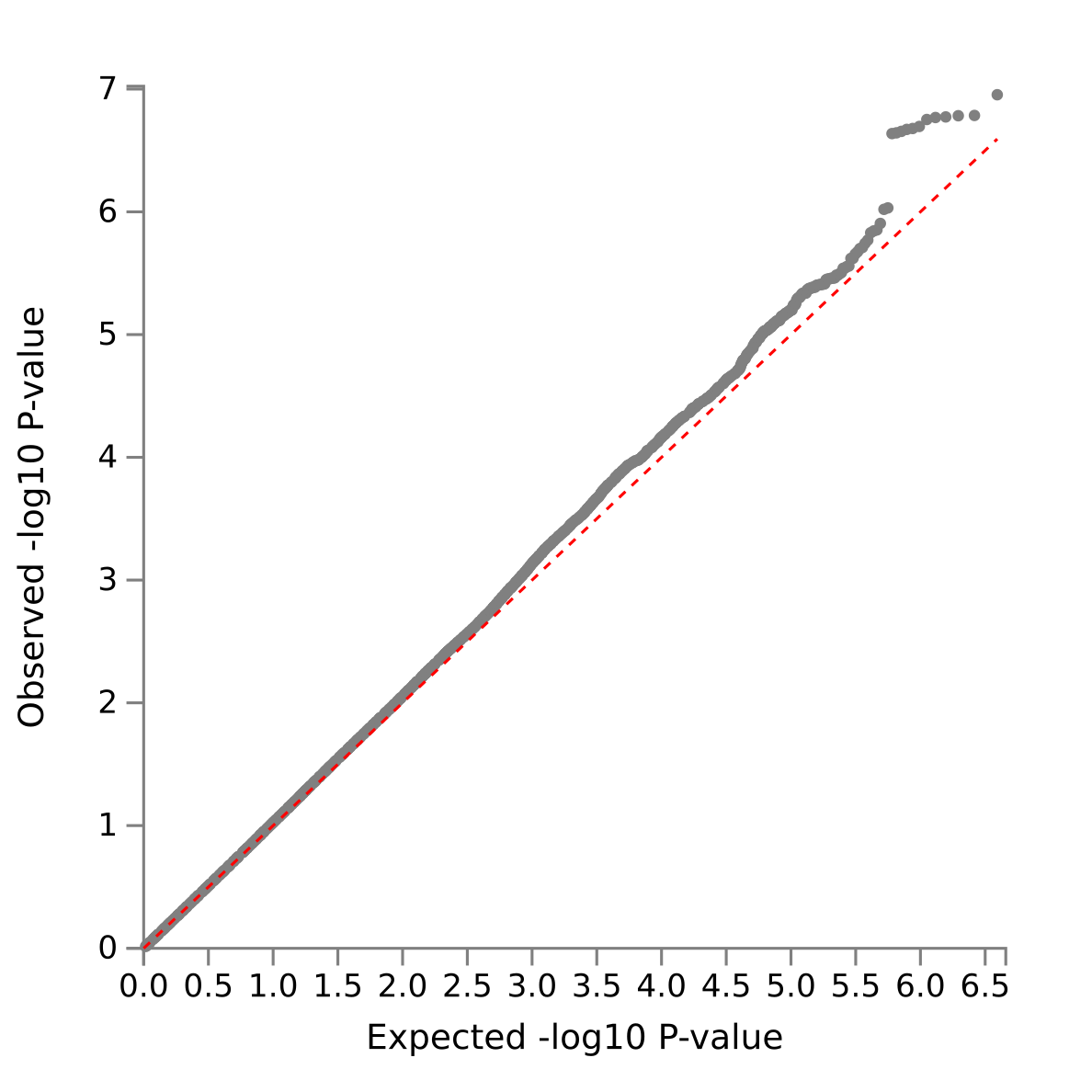
**

**Figure S26. Quantile-quantile (QQ) plot and Manhattan plot of genome-wide association study (GWAS) results of the lifetime impulsivity factor measured by the Mood Disorder Questionnaire (MDQ) in affected participants of European ancestry (N=19,859).**

*GWAS was performed with REGENIE covarying for the first ten ancestry principal components and genotyping batch. Manhattan and QQ plots were produced using FUMA.*

**
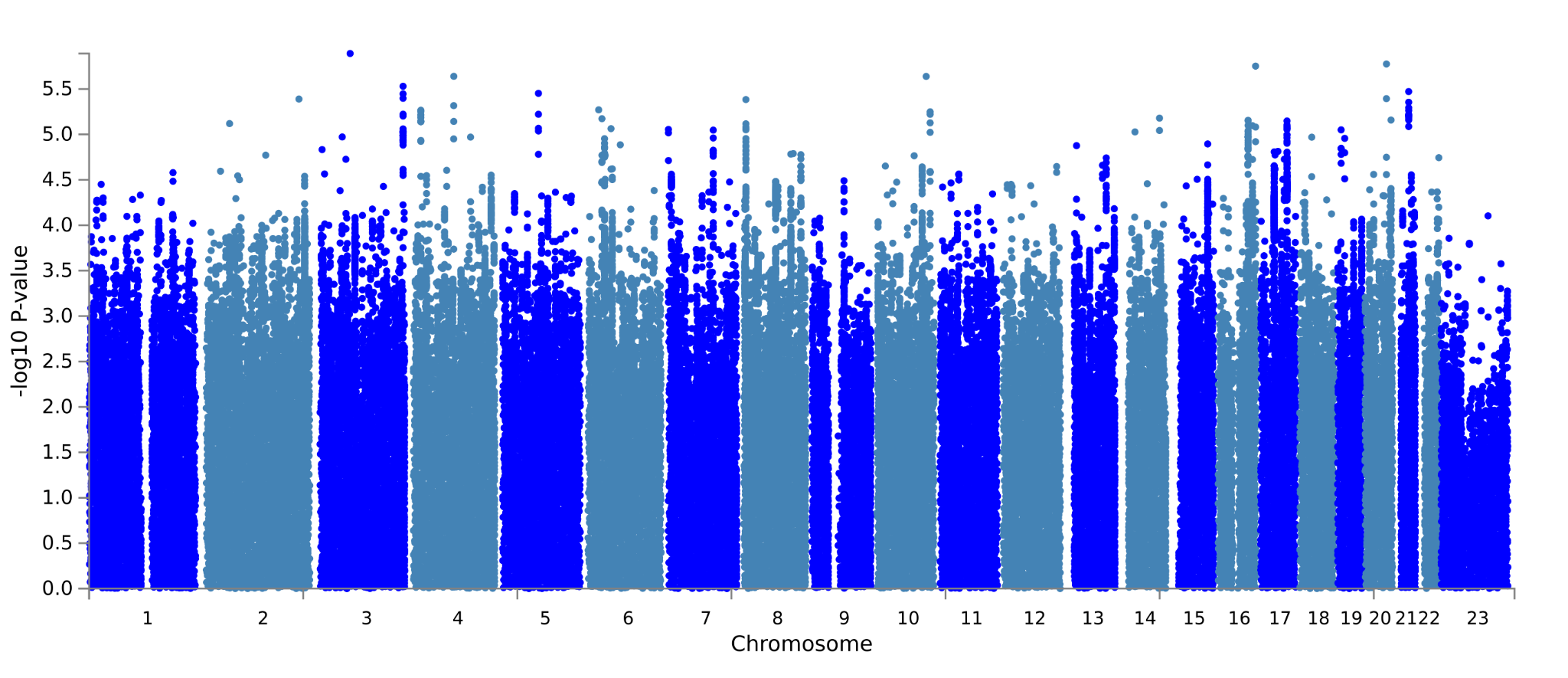
**

**
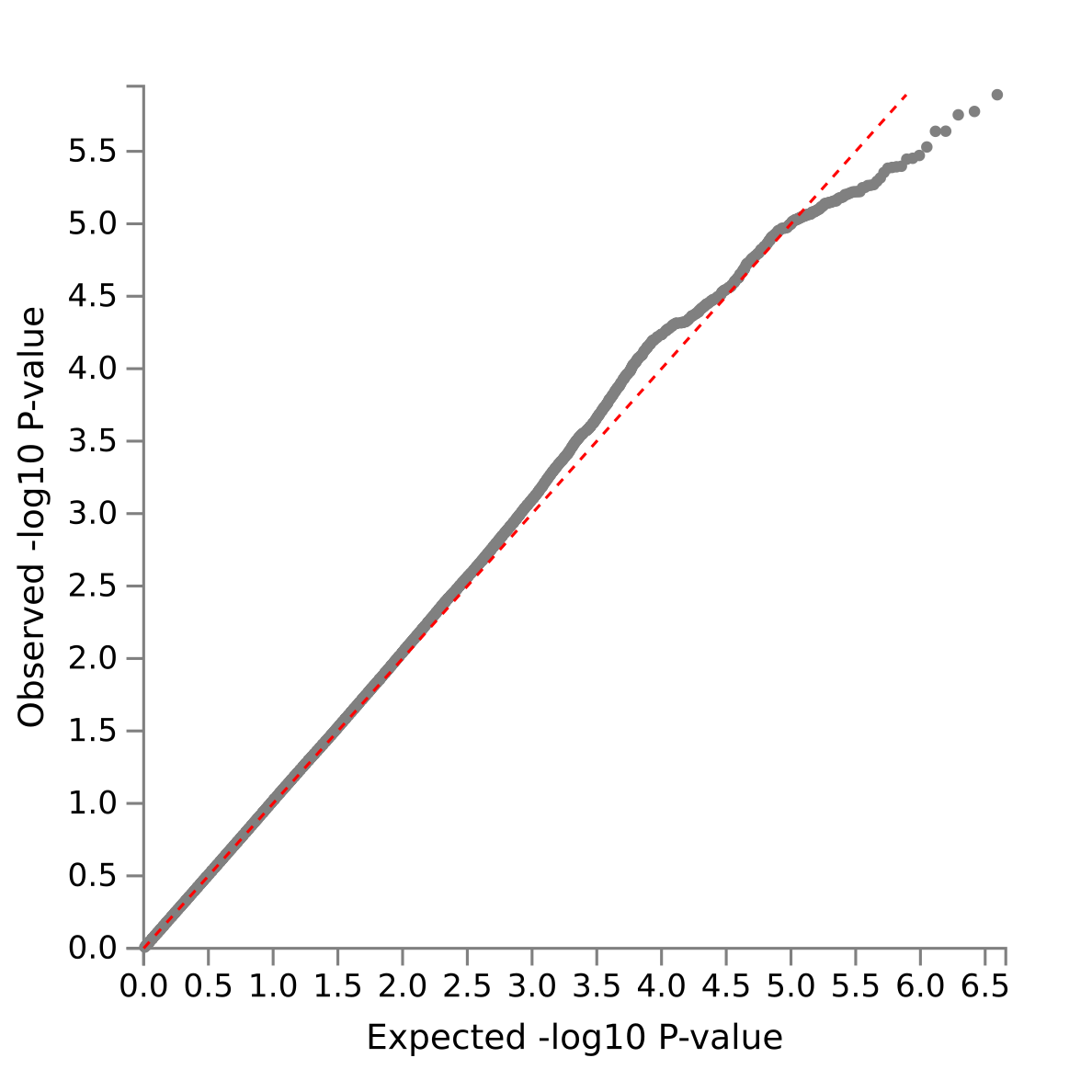
**

**Figure S27. Scatter plot of affected participants’ lifetime manic symptoms measured by the Mood Disorder Questionnaire (MDQ) and current posttraumatic stress disorder (PTSD) symptoms measured by the 6 item PTSD Checklist (PCL-6). Lifetime manic symptoms were scored 0-12 and current PTSD symptoms were scored 6-30.**

**
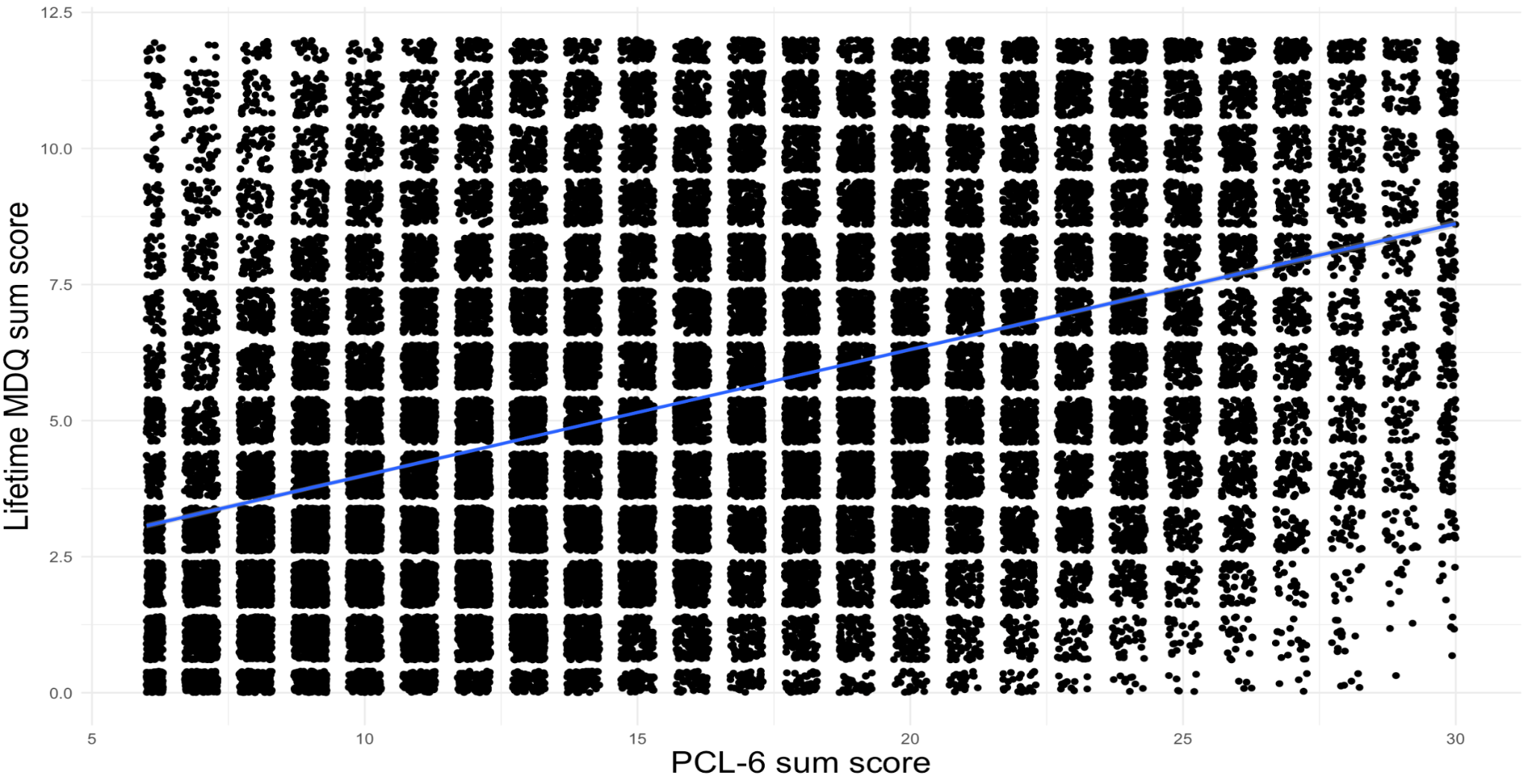
**

**Figure S28. Flow-chart detailing how Genetic Links to Anxiety and Depression (GLAD) Study and COVID-19 Psychiatry and Neurological Genetics Study participants from the NIHR Bioresource (COPING NBR) were categorised as either “affected” or “unaffected’ by major depressive disorder (MDD) and/or any anxiety disorder. MDQ=Mood Disorder Questionnaire.**


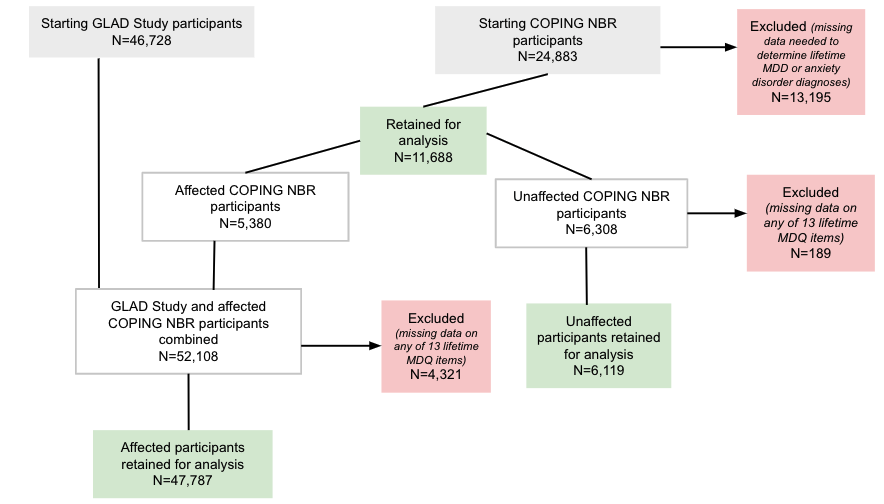

Supplement: Supplementary file 1 — Figure S1. Correlations between concurrent Mood Disorder Questionnaire (MDQ) items in individuals affected by major depressive disorder (MDD) and/or an anxiety disorder. Figure S2. Correlations between lifetime Mood Disorder Questionnaire (MDQ) items in individuals affected by major depressive disorder (MDD) and/or an anxiety disorder. Figure S3. Correlations between lifetime Mood Disorder Questionnaire (MDQ) items in individuals unaffected by major depressive disorder (MDD) and/or an anxiety disorder. Figure S4. Exploratory factor analysis (EFA): one factor solution of 12 concurrent Mood Disorder Questionnaire (MDQ) items in affected participants. Figure S5. Exploratory factor analysis (EFA): two factor solution of 12 concurrent Mood Disorder Questionnaire (MDQ) items in affected participants. Figure S6. Exploratory factor analysis (EFA): three factor solution of 12 concurrent Mood Disorder Questionnaire (MDQ) items in affected participants. Figure S7. Exploratory factor analysis (EFA): four factor solution of 12 concurrent Mood Disorder Questionnaire (MDQ) items in affected participants. Figure S8. Exploratory factor analysis (EFA): one factor solution of 12 lifetime Mood Disorder Questionnaire (MDQ) items in affected participants. Figure S9. Exploratory factor analysis (EFA): two factor solution of 12 lifetime Mood Disorder Questionnaire (MDQ) items in affected participants. Figure S10. Exploratory factor analysis (EFA): three factor solution of 12 lifetime Mood Disorder Questionnaire (MDQ) items in affected participants. Figure S11. Exploratory factor analysis (EFA): four factor solution of 12 lifetime Mood Disorder Questionnaire (MDQ) items in affected participants. Figure S12. Exploratory factor analysis (EFA): one factor solution of 12 lifetime Mood Disorder Questionnaire (MDQ) items in unaffected participants. Figure S13. Exploratory factor analysis (EFA): two factor solution of 12 lifetime Mood Disorder Questionnaire (MDQ) items in unaffected participants. [file AJMG-192-147-s003.docx]
