# Supplementary material for: Genetic examination of the Mood Disorder Questionnaire and its relationship with bipolar disorder
Source: Am J Med Genet B Neuropsychiatr Genet. 2023 May 13;192(7-8):147–60. doi: 10.1002/ajmg.b.32938 (PMC10952822; doi:10.1002/ajmg.b.32938)
Supplement: Supplementary file 2 — Data S1. Supporting Information. [file AJMG-192-147-s001.docx]

**Genetic examination of the Mood Disorder Questionnaire and its relationship with bipolar disorder**

**1 Supplementary methods**

**1.1 Major depressive disorder and anxiety disorder diagnostic criteria**

COPING NBR participants who met symptom-based diagnostic criteria for major depressive disorder (MDD) and/or any anxiety disorder were combined with GLAD participants to create a cohort of “affected” participants. The category of “any anxiety disorder” included generalised anxiety disorder (GAD), specific phobia, social phobia, panic disorder, and agoraphobia. COPING NBR participants who did not meet criteria for MDD and any anxiety disorder were categorised as unaffected participants. These criteria were based upon the MDD, GAD, specific phobia, social anxiety disorder, panic disorder, and agoraphobia modules from an adapted version of the short form Composite International Diagnostic Interview (CIDI-SF) in the COPING baseline survey. Diagnostic algorithms were written in R to categorise COPING NBR participants as having a lifetime symptom-based diagnosis for these disorders if their responses on the CIDI-SF corresponded to Diagnostic Statistical Manual V (DSM-V) criteria. Further detail of these symptom-based diagnoses have been described elsewhere [(Davies *et al.*, 2022)](https://paperpile.com/c/inSqYe/2mF1o).

**1.2 Self-reported bipolar diagnosis**

GLAD participants have two opportunities to self-report a diagnosis of bipolar disorder. The first is in the Mental Health Disorders (MHD) section and the second is in the MDQ section of the survey. Participants who self-reported that they had received a diagnosis of bipolar disorder by a professional in one of the two questions but had missing data in the other were categorised as a bipolar disorder case. Participants who answered “No” to one question but had missing data on the other question, had answers that did not match, or had missing data on both of these questions were excluded from analyses. NBR participants have one opportunity to self-report a diagnosis of bipolar disorder in the MHD section of the COPING survey. Participants who had missing data for this question were excluded from analyses.

**1.3 Mood Disorder Questionnaire screener**

A positive screen in the MDQ requires that seven or more items be endorsed, that at least several of the items co-occurred, and that the symptoms caused at least moderate impairment [(Hirschfeld *et al.*, 2000, 2003)](https://paperpile.com/c/inSqYe/o9MS+g2Fq).

**1.4 Statistical analyses**

***1.4.1 Exploratory and confirmatory factor analyses***

Exploratory factor analysis (EFA) is a classic latent variable technique which finds latent variables based on the correlation structure of the manifest input variables (here, the MDQ items). EFA is distinguished from confirmatory factor analysis (CFA) in that in CFA the model is determined by the researcher based on an underlying theory or from a model identified in the EFA (Mair, 2018).

In our paper, each sample of participants was randomly split into two (without replacement) using the “rsample” R package; 70% for EFA and 30% for CFA. Before splitting the sample, the nearZeroVar R package was used to diagnose variables that have one unique value (i.e., are zero variance predictors) or are near zero variance predictors.

Polychoric correlation matrices were then computed for all items using the “polycor” R package; the answers to the MDQ were binary and tetrachoric correlations are a special case of polychoric correlations. To check for multicollinearity of the items, the determinant of the matrix was computed. If the determinant was greater than 0.00001 then the matrices were inspected for highly correlated items and were removed accordingly. To test for singularity, the matrices were inspected for values <0.3. Any items that correlated <0.3 with all other items were removed from the analysis. The ordinal alpha statistic, the Kaiser-Meyer-Olkin (KMO) statistic [(Kaiser, 1974)](https://paperpile.com/c/inSqYe/O2E7N) and Bartlett’s Test of Sphericity [(Bartlett, 1950)](https://paperpile.com/c/inSqYe/9LHdS) were computed to assess whether the data were appropriate for exploratory factor analysis (EFA). Parallel analysis [(Horn, 1965)](https://paperpile.com/c/inSqYe/W725Z), Very Simple Structure (VSS) [(Revelle and Rocklin, 1979)](https://paperpile.com/c/inSqYe/kKX3p), and Velicer’s Minimum Average Partial (MAP) criterion [(Velicer, 1976)](https://paperpile.com/c/inSqYe/Lp1rh) were estimated to gain an initial idea of the number of factors suited to the data.

The EFA was performed in the 70% of the sample using the minimum residuals method in the “psych” R package. Factors were allowed to correlate using oblimin rotation. First, we performed EFA specifying a one factor solution to investigate whether all the items loaded onto one factor and therefore represent a single latent construct. We then continued performing EFA, by adding one more factor at a time, until the minimum number of items loading onto a factor reached one.

A number of fit criteria were used to assess which factor model fit the data best: the root mean square error of approximation (RMSEA) <0.5 (good fit) or 0.06-0.08 (fair fit), Tucker–Lewis Index (TLI) ≥0.90, root mean square residuals (RMSR) as small as possible (preferably <0.08) (Mair, 2018), and small Bayesian Information Criterion (BIC) relative to other factor solutions. Items were retained in a factor if the loading was ≥0.3 and greater than all loadings on other factors. Where multiple models showed comparable fit statistics, the model that encompassed the highest number of items was selected and models where the minimum number of items per factor was greater than three were preferred.

To validate the EFA-derived model, CFA was conducted on the remaining 30% of the sample using the “lavaan” R package. A number of fit statistics were interpreted: the TLI ≥0.9, Standardised Root Mean Square of the Residuals (RSMR) as small as possible, a smaller BIC compared to other factor solutions and Comparative Fit Index (CFI) ≥0.9. The CFA was then applied to the whole sample using the lavaan R package to provide overview fit statistics.

Factor scores for each factor in the best-fitting model were computed for every participant to gain individual values for participants which represent their “placement” on each factor. Factor scores were computed with the “lav_predict” function from the “lavaan” R package using the Empirical Bayes Modal (EBM) method. The factor scores were then transformed using a rank-based inverse normal transformation (INT) using the “RNOmni” R package and standardised using base R functions. Prior to transformation, ties in the data were broken using the “surveillance” R package.

**1.5 Genetic analyses**

***1.5.1 Genotyping, imputation and quality control***

*1.5.1.1 Genotyping*

Quality assurance measures were calculated by ThermoFisher: samples with a dish QC value ≥0.82 (capturing the resolution of true signal from background noise on the genotyping array) and an initial call rate ≥0.97 were retained. Variants were recommended for inclusion if they were genotyped with high resolution (classified as "PolyHighResolution", "NoMinorHom", or "MonoHighResolution" by ThermoFisher). Data passing quality assurance was transferred to the Social, Genetic, and Developmental Psychiatry Centre at King's College London for further quality control, adapted from previous pipelines [(Coleman *et al.*, 2016)](https://paperpile.com/c/inSqYe/ajgAw).

Data for GLAD and COPING NBR were processed separately following the same pipeline. An initial set of quality control was performed to determine sample ancestry. This consisted of excluding variants with a minor allele frequency (MAF) <0.01, variants and individuals with a call rate <95%, and variants with Hardy-Weinberg p<10^-10^. Additional checks were performed on individuals to exclude outliers for sex discrepancies, heterozygosity, and relatedness. Samples were merged with data from Phase 3 of the 1000 Genomes project and principal component analyses were performed on genome-wide genotype data. Samples clustering with known individuals from European ancestries in the 1000 Genomes project formed the majority of the genotyped GLAD and COPING NBR cohorts (96% and 98% respectively; **figure S18**) and so further analyses were restricted only to these participants. Quality control was repeated, on raw data restricted to European ancestry participants. This comprised the same measures as above.

*1.5.1.2 Imputation*

For GLAD and COPING NBR separately, high quality genotype data was lifted to build 38 of the human genome and imputed to TopMed freeze 8, using version 1.5.7 of the dedicated imputation server provided by the University of Michigan [(Taliun *et al.*, 2021)](https://paperpile.com/c/inSqYe/Pse9f) with prior phasing using EAGLE2 [(Loh *et al.*, 2016)](https://paperpile.com/c/inSqYe/ueCYF). Following imputation, data (in variant call format [VCF] files) was restricted to variants with MAF ≥ 0.001 and imputation R^2^ ≥ 0.3. Post-imputation VCFs were updated to include sex information and rsIDs, which were collected from the Single Nucleotide Polymorphism Database, build 153.

*1.5.1.3 GLAD & NBR merge*

Data from GLAD and COPING NBR were merged post-imputation using bcftools, and converted to PLINK2 pfile format, retaining genotype dosage information [(Chang *et al.*, 2015)](https://paperpile.com/c/inSqYe/J1g8o). Only bi-allelic SNPs were retained in the resulting merged pfiles. Post-merge, the data was filtered with a MAF threshold of 0.01 and a variant missingness of 0.02. Duplicate samples, related individuals with pihat > 0.1875, and samples with mismatched sex were also excluded.

**1.6 Post-hoc phenotypic correlation between manic symptoms and PTSD symptoms**

Based on the finding that the lifetime manic symptom sum score, which represents the total number of lifetime manic symptoms that a participant reported, was most genetically correlated with PTSD (**figure 3**), we calculated the phenotypic correlation between the lifetime manic symptom sum score and current PTSD symptoms in affected participants. PTSD symptoms were based on answers to the 6-item PTSD checklist (PCL-6), scored 6-30. As per the MDQ, GLAD participants answered the PCL-6 in the GLAD sign-up questionnaire and COPING participants answered the PCL-6 in the COPING baseline questionnaire. Participants were asked six questions relating to their experience of PTSD symptoms:

1. *Repeated, disturbing memories, thoughts, or images of a stressful experience?*
2. *Feeling very upset when something reminded you of a stressful experience?*
3. *Avoiding activities or situations because they reminded you of a stressful situation?*
4. *Feeling distant or cut off from other people?*
5. *Feeling irritable or having angry outbursts?*
6. *Difficulty concentrating?*

Participants could answer with *“Not at all”*, *“A little bit”*, *“Moderately”*, *“Quite a bit”*, and *“Extremely”* based on how they were feeling over the past month. These were coded numerically 1-6. Answers were then summed to create a sum score ranging 6-30 with lower scores representing lower levels of PTSD symptoms and higher scores representing higher levels of PTSD symptoms. Only participants with complete data on all six questions were included in the sum score. We used R to calculate Pearson’s correlation between the lifetime manic symptom sum score and the current PTSD symptom sum score.

**2 Supplementary results**

**2.1 Factor analyses**

***2.1.1 Concurrent manic symptoms in participants affected by MDD and/or anxiety***

As mentioned in the main paper, the item “concurrent more active” was removed from the factor analysis due to having a correlation of 0.87 with “concurrent more energy” (“concurrent more active” was removed due to lower endorsement rate of the two items) (**table 1**). This left 12 concurrent MDQ items remaining. In order to avoid including participants who (now) only endorsed one out of the 12 concurrent MDQ items, we removed 82 participants. These were participants who initially had a concurrent manic symptom sum score of 2 and now only had a score of 1 (because “concurrent more active” had been removed from the score). This left a final N of 29,899. None of the remaining items were correlated <0.3 so no further items were removed. None of the items had near zero variance which showed that the data was suitable to be randomly split into an EFA and CFA sample. After the sample was split, the determinant of the matrix of the EFA sample was 0.003 which suggested that multicollinearity was not a problem and therefore no further items were removed (**table S2**). The ordinal alpha statistic, Kaiser-Meier-Olkin (KMO) statistic and Bartlett’s test of sphericity p-value also demonstrated that the data were suitable for factor analysis (**table S2**).

After performing EFA in 70% of the sample (N=20,929), the one factor model showed that concurrent irritability, racing thoughts, and concentration problems did not load onto the factor. A decision was made to keep these items in the factor analysis because the correlation matrix suggested they would form their own factor and to keep comparability with the lifetime MDQ items factor analysis. The three factor solution was selected as the final model because it showed the best fit statistics while retaining at least three items per factor (**figure 2; table S3**). All fit statistics demonstrated that the three factor model was a good fit for the data (**table S4a**). Factor one, two and three included six, three, and three items respectively. We named the three factors according to their loaded items: *energy/activity, cognitive, and impulsivity* (**figure 2;** **figure S6**). The *energy/activity* and *impulsivity factors* correlated with each other at 0.54. As expected, concurrent “irritability”, “racing thoughts”, and “concentration problems” formed their own factor. This factor (*concurrent* *cognitive*) did not correlate with the other two factors (**figure 2; figure S7; table S4b**) which reflected the absence of correlations between “concentration problems”, “racing thoughts”, and “irritability” and the other MDQ items (**figure S1**). It is important to note that “irritability” had a fairly weak loading onto the cognitive factor (**figure S6**). The model was confirmed in CFA on the remaining 30% of the sample (N=8,970) and showed good fit statistics (**table S5**). The three factor model was then applied to the full sample (N=29,899) to provide overall model fit statistics (**table S5**).

***2.1.2 Lifetime manic symptoms in participants affected by MDD and/or anxiety***

As mentioned in the main paper, the item “more active” was removed from the factor analysis due to having a correlation of 0.90 with “more energy”. None of the remaining items were correlated <0.3 so no further items were removed. None of the items had near zero variance which showed that the data was suitable to be randomly split into an EFA and CFA sample. After splitting the sample, the determinant of the matrix in the EFA sample was 0.0002 which suggested that multicollinearity was not a problem and therefore no further items were removed (**table S1**). The ordinal alpha statistic, KMO statistic and Bartlett’s test of sphericity p-value also demonstrated that the data were suitable for factor analysis (**table S1**).

After performing EFA in 70% of the sample (N=33,450), the three factor solution was selected as the final model because it showed the best fit statistics while retaining at least three items per factor (**figure 2; table S7**). Factor one, two and three included six, three, and three items respectively. We named the three factors according to the loaded items: *energy/activity, cognitive and impulsivity*. These factors perfectly mirrored those identified in the concurrent MDQ items factor analysis, with the exception that all factors correlated with each other (*r≥* 0.55) (**table S7b**). CFA on the remaining 30% of the sample (N=14,337) confirmed that the three factor model fit the data very well (**table S8**). CFA was then applied to the full sample (N=47,787) to provide overall model fit statistics (**table S8**).

***2.1.3 Lifetime manic symptoms in participants unaffected by MDD and/or anxiety***

As mentioned in the main paper, the item “more active” was removed from the factor analysis due to having a correlation of 0.90 with “more energy”. None of the remaining items were correlated <0.3 so no further items were removed. Five of the items had near zero variance (the items “hyperactivity”, “more talkative”, “more sociable”, “risky behaviour”, and “reckless spending”) which showed that the data may not have been suitable to be randomly split into an EFA and CFA sample. Therefore, after splitting the sample, the frequencies of these five items were compared between the EFA and CFA sample. We were satisfied that the frequencies were comparable and we continued with the factor analysis. The determinant of the matrix in the EFA sample was 0.0004 which suggested that multicollinearity was not a problem and therefore no further items were removed. The ordinal alpha statistic, KMO statistic and Bartlett’s test of sphericity p-value also demonstrated that the data were suitable for factor analysis (**table S2**).

After performing EFA in 70% of the sample (N=4,283), none of the factor solutions showed adequate fit (**table S10**). Therefore, no solution was carried forward to CFA.

**2.2 Post-hoc phenotypic correlation between manic symptoms and posttraumatic stress disorder symptoms**

Based on the unexpected finding that the lifetime manic symptom sum score was most strongly genetically correlated with PTSD (**figure 3; table S15**), we calculated the phenotypic correlation between lifetime manic symptom sum score and current PTSD symptoms in affected participants. PTSD symptoms were based on answers to the 6-item PTSD checklist (PCL-6) and were scored 6-30. The phenotypic correlation was far lower than the genetic correlation (*r*_ph_=0.41, p<2x10^-16^). See supplementary methods for more details of the PCL-6 and **figure** **S27** for a scatter plot of the two measures.

**3 Supplementary discussion**

**3.1 Genetics of manic symptom subgroups**

We hypothesised that the genetics of the symptom subgroups identified in the factor analyses would differentially genetically correlate with other psychiatric and behavioural traits. We did not make any *a priori* assumptions about which subgroups would correlate with which traits. The concurrent symptom subgroups had no significant genetic correlations with other traits but *concurrent* *energy/activity* and *concurrent* *impulsivity* significantly genetically correlated with each other. Neither of these subgroups genetically correlated with *concurrent cognitive* (**table S12**). This mirrors the pattern of phenotypic correlations between the factors found in our study (**figure 2**). The *concurrent cognitive* symptom subgroup was also not genetically correlated with the overall concurrent manic symptom sum score. This, combined with the phenotypic results, suggest that the MDQ items within this subgroup do not capture the same trait as the other MDQ items when measured as concurrent. This suggests that the concurrent MDQ lacks internal consistency and is therefore unreliable.

This lack of internal consistency may explain why the concurrent manic symptom phenotypes were not significantly genetically correlated with any of the external traits in our study (**table S14**). Compared to the lifetime MDQ items, the GWASs of the concurrent MDQ items already had reduced statistical power (smaller N [11,568 vs. 19,859] and smaller variance [range 2-12 items vs. 0-12 items]). This was likely exacerbated by the three concurrent *cognitive symptoms* being included in the composite score despite, as learned from our findings, operationalising a fundamentally different trait to the other nine items. This may also explain the non-significant heritability of the sum score.

The results with the lifetime manic symptom subgroups, where we *did* find significant genetic correlations with other traits, confirms that our hypothesis (that manic symptoms would show significant positive genetic correlations with bipolar disorder) was not supported. The symptom subgroups showed broadly similar *r_g_* estimates with the traits to each other as well as the overall sum score and none differed from each other significantly. This is reflected by the finding that the genetic correlations between the lifetime manic symptom subgroups all hovered around 1 (**table S13**). These estimates far exceed the phenotypic correlations found in the factor analysis (**figure S6**).

**Supplementary references**

[Bartlett, M.S. (1950) ‘Tests of significance in factor analysis’, *British journal of psychology* , 3, pp. 77–85.](http://paperpile.com/b/inSqYe/9LHdS)

[Chang, C.C. *et al.* (2015) ‘Second-generation PLINK: rising to the challenge of larger and richer datasets’, *GigaScience*, 4, p. 7.](http://paperpile.com/b/inSqYe/J1g8o)

[Coleman, J.R.I. *et al.* (2016) ‘Quality control, imputation and analysis of genome-wide genotyping data from the Illumina HumanCoreExome microarray’, *Briefings in functional genomics*, 15(4), pp. 298–304.](http://paperpile.com/b/inSqYe/ajgAw)

[Davies, M.R. *et al.* (2022) ‘Comparison of symptom-based versus self-reported diagnostic measures of anxiety and depression disorders in the GLAD and COPING cohorts’, *Journal of anxiety disorders*, 85, p. 102491.](http://paperpile.com/b/inSqYe/2mF1o)

[Hirschfeld, R.M. *et al.* (2000) ‘Development and validation of a screening instrument for bipolar spectrum disorder: the Mood Disorder Questionnaire’, *The American journal of psychiatry*, 157(11), pp. 1873–1875.](http://paperpile.com/b/inSqYe/o9MS)

[Hirschfeld, R.M.A. *et al.* (2003) ‘Validity of the mood disorder questionnaire: a general population study’, *The American journal of psychiatry*, 160(1), pp. 178–180.](http://paperpile.com/b/inSqYe/g2Fq)

[Horn, J.L. (1965) ‘A RATIONALE AND TEST FOR THE NUMBER OF FACTORS IN FACTOR ANALYSIS’, *Psychometrika*, 30, pp. 179–185.](http://paperpile.com/b/inSqYe/W725Z)

[Kaiser, H.F. (1974) ‘An index of factorial simplicity’, *Psychometrika*, 39(1), pp. 31–36.](http://paperpile.com/b/inSqYe/O2E7N)

[Loh, P.-R. *et al.* (2016) ‘Reference-based phasing using the Haplotype Reference Consortium panel’, *Nature genetics*, 48(11), pp. 1443–1448.](http://paperpile.com/b/inSqYe/ueCYF)

[Revelle, W. and Rocklin, T. (1979) ‘Very Simple Structure: An Alternative Procedure For Estimating The Optimal Number Of Interpretable Factors’, *Multivariate behavioral research*, 14(4), pp. 403–414.](http://paperpile.com/b/inSqYe/kKX3p)

[Taliun, D. *et al.* (2021) ‘Sequencing of 53,831 diverse genomes from the NHLBI TOPMed Program’, *Nature*, 590(7845), pp. 290–299.](http://paperpile.com/b/inSqYe/Pse9f)

[Velicer, W.F. (1976) ‘Determining the number of components from the matrix of partial correlations’, *Psychometrika*, 41(3), pp. 321–327.](http://paperpile.com/b/inSqYe/Lp1rh)

Mair, Patrick 2018. Modern Psychometrics with R, *Springer*
